# N‐Doped Nonalternant Molecular Bowl/Saddle Hybrids

**DOI:** 10.1002/anie.202516881

**Published:** 2025-09-30

**Authors:** Shuhai Qiu, Kai Chen, Ziqi Deng, Xuan Jin, Zuoyu Li, Guogang Liu, Li Zhang, Wei Jiang, Teng‐Teng Chen, Junzhi Liu, Zhaohui Wang

**Affiliations:** ^1^ Department of Chemistry The University of Hong Kong Pokfulam Road Hong Kong China; ^2^ School of Chemical Sciences University of Chinese Academy of Sciences Beijing 100049 China; ^3^ Department of Chemistry The Hong Kong University of Science and Technology Hong Kong China; ^4^ Key Laboratory for Advanced Materials and Joint International Research Laboratory of Precision Chemistry and Molecular Engineering Feringa Nobel Prize Scientist Joint Research Center Frontiers Science Center for Materiobiology and Dynamic Chemistry Institute of Fine Chemicals School of Chemistry and Molecular Engineering East China University of Science and Technology Shanghai 200237 China; ^5^ Key Laboratory of Organic Optoelectronics and Molecular Engineering Department of Chemistry Tsinghua University Beijing 100084 China; ^6^ State Key Laboratory of Synthetic Chemistry HKU‐CAS Joint Laboratory on New Materials and Shanghai‐Hong Kong Joint Laboratory on Chemical Synthesis The University of Hong Kong Pokfulam Road Hong Kong China; ^7^ Materials Innovation Institute for Life Sciences and Energy (MILES) HKU‐SIRI Shenzhen China

**Keywords:** Bowl chirality, Molecular bowl, Molecular saddle, N‐doping, Nonalternant hydrocarbons

## Abstract

Herein, we report the straightforward synthesis and properties of N‐doped molecular bowl/saddle hybrids with nonalternant topologies via successive palladium‐catalyzed annulations. Structural evolutions from twisted, bowl‐shaped to bowl/saddle‐hybridized structures are involved during the core‐expansion process, as confirmed by X‐ray crystallographic analyses. Notably, this resultant bowl/saddle‐hybridized molecular carbon shows unique molecular dynamics, as revealed by ^1^H NMR, chiral HPLC analyses and theoretical calculations. These molecules exhibit interesting photophysical properties, including moderate fluorescence quantum yields and narrowband emissions. Steady‐state and transient spectroscopy reveal the different photophysical properties in these molecular bowl and bowl/saddle hybrids. Moreover, the molecular bowl/saddle hybrid exhibits the shape‐adaptive character, and allow for multiple bindings with fullerene guests in a stoichiometry ratio of 1:3, which is seldomly observed in most nonplanar molecular carbons. This study not only presents an efficient strategy to construct a new family of N‐doped nonalternant molecular bowl/saddle hybrids, but also provides insights into molecular design of topological nanocarbons with potential applications of chiral materials and organic electronics.

## Graphical abstract

N‐Doped molecular bowl/saddle hybrids were developed via core‐expansion synthetic strategy. Owing to the structural features containing both positive and negative curvatures, these molecules exhibit unique molecular dynamics, photophysical properties and multiple guest bindings.

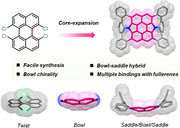

## Introduction

**Figure 1:**
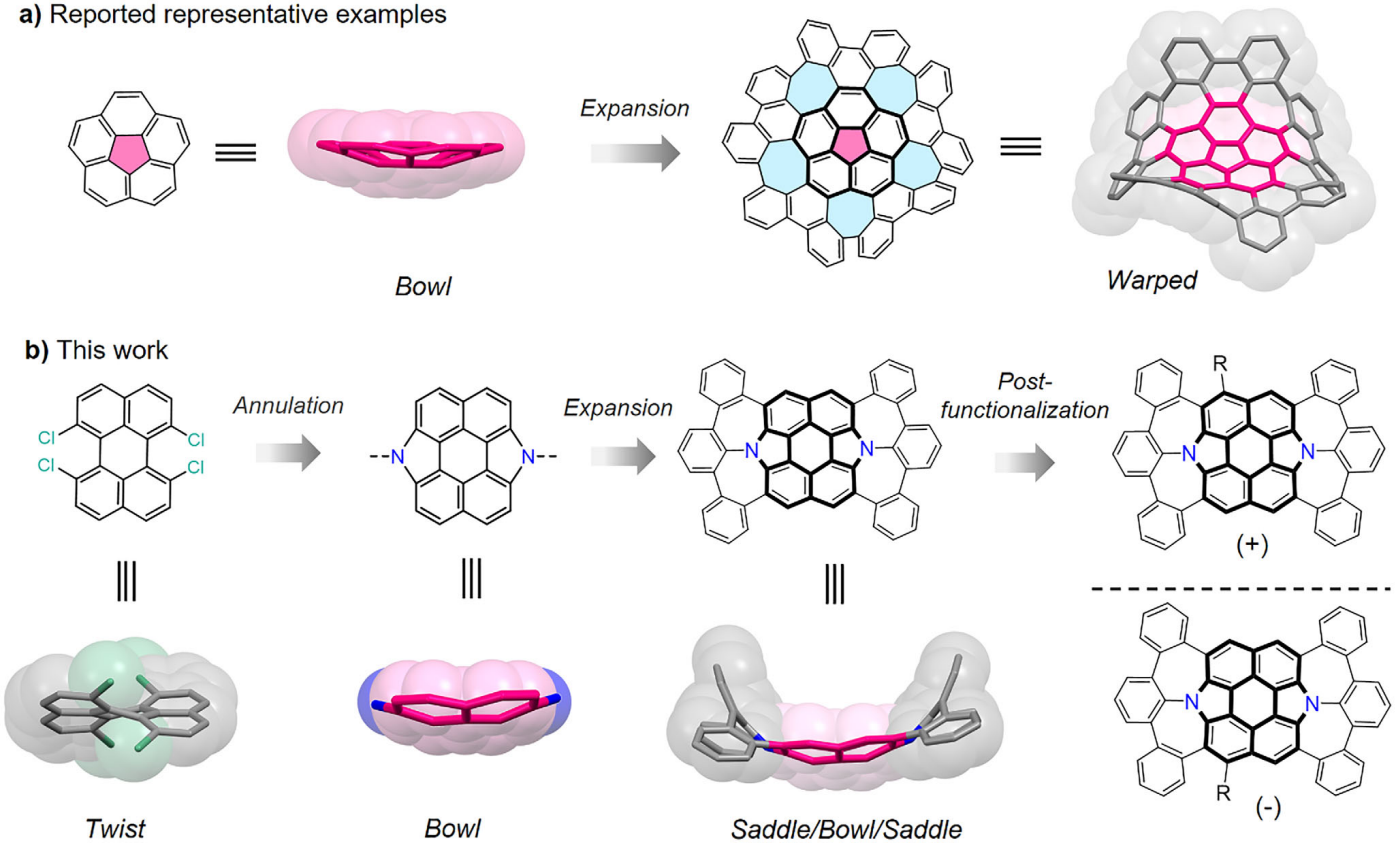
a) Reported representative examples of curved molecular carbons containing a central corannulene unit and five embedded heptagons, synthesized from bowl‐shaped corannulene scaffold. b) Synthetic strategy for N‐doped chiral molecular bowl/saddle hybrids in this work.

Topological molecular carbons have sparked wide research interests due to their unique geometric structures, electronic properties, and diverse applications in past decades.^[^ ^1^, ^2^, ^3^, ^4^, ^5^ ^]^ Among them, molecular carbons with curved π‐surfaces, such as fullerenes,^[^ ^6^ ^]^ molecular bowls,^[^ ^7^, ^8^ ^]^ and saddles,^[^ ^9^, ^10^ ^]^ are of particular interest on account of intriguing properties, including aromaticity,^[^ ^11^, ^12^ ^]^ molecular dynamics,^[^ ^13^, ^14^ ^]^ chirality,^[^ ^15^, ^16^ ^]^ and host–guest behaviors.^[^ ^17^, ^18^, ^19^, ^20^ ^]^ In general, their topological structures could be described by the Gaussian curvature ***K***, which is defined as the product of the principal curvatures κ_1_ and κ_2_, the maximum and minimum values among all the curvatures at a given point of a curved surface. For instance, incorporating pentagons in the sp^2^ hexagonal framework induces positive curvature with ***K *** > 0, and the prototypical examples include bowl‐shaped corannulenes^[^ ^21^, ^22^ ^]^ and sumanenes,^[^ ^7^ ^]^ which are the minimum subunits of C_60_ and higher fullerenes. In contrast, introducing rings greater than hexagons into polyaromatics results in negative curvature with ***K *** < 0, forming saddle‐shaped structures as the cutouts of Schwarzite carbon.^[^ ^23^, ^24^ ^]^ Hitherto, with the development of synthetic methodologies, large amounts of molecular bowls^[^ ^25^, ^26^, ^27^, ^28^, ^29^, ^30^, ^31^ ^]^ and saddles^[^ ^10^, ^32^, ^33^, ^34^, ^35^, ^36^, ^37^, ^38^, ^39^, ^40^, ^41^ ^]^ containing heteroatoms and in diverse molecular sizes have been reported. In addition, rationally arranging pentagons with heptagons in the hexagonal skeleton is an effective strategy for developing novel topological molecular carbons.^[^ ^42^, ^43^, ^44^ ^]^ For example, the grossly warped corannulene‐based nanographenes (Figure 1a) showing intriguing molecular dynamics and chiroptical properties have been reported by Itami,^[^ ^45^ ^]^ Zhang^[^ ^46^ ^]^ and coworkers. Curved nanographenes integrating bowl‐shaped subunits with one heptagon or octagon ring were also developed by Martín^[^ ^47^ ^]^ and Zhang,^[^ ^48^ ^]^ respectively. In spite of these impressive pioneering works, studies on molecular carbons containing both positive and negative curvatures remain limited due to the difficulty in harnessing strain generated by curvatures, and there is large space to explore topological π‐systems containing both positive and negative curvatures.

The early‐stage synthetic approaches toward well‐defined curved molecular carbons focus on harsh reaction conditions, such as flash vacuum pyrolysis^[^ ^49^, ^50^ ^]^ and on‐surface synthesis,^[^ ^51^ ^]^ in order to overcome the inner strain. In contrast, solution‐phase synthesis, such as Scholl reaction, has been proved to be one of the most powerful methodologies to access these curved molecular architectures.^[^ ^52^, ^53^ ^]^ Nevertheless, for the nitrogen (N)‐doped molecular carbons, there are only limited examples using Scholl reaction due to the poor stability of electron‐rich N atoms in the presence of acids or oxidants.^[^ ^54^, ^55^, ^56^ ^]^ Instead, palladium (Pd)‐catalyzed annulation seems to be more efficient, and various N‐doped curved π‐systems have been successfully prepared.^[^ ^57^, ^58^, ^59^ ^]^ Inspired by the intriguing molecular topology as well as well‐developed synthetic methodologies of curved molecular carbons, we envision that it is plausible to construct N‐doped nonalternant molecular carbons integrating both positive and negative curvatures in the same molecular skeleton through rational molecular design.

In this work, we report the synthesis and characterization of N‐doped molecular bowl/saddle hybrids via core‐expansion synthetic strategy (Figure 1b). Starting from twisted tetrachloroperylene, N‐doped molecular bowl featuring the segments of N‐doped C_70_ was synthesized by Pd‐catalyzed double annulations. Further using Pd‐catalyzed direct arylations, two saddle subunits containing N‐doped heptalenes were constructed fusing on the bowl‐shaped core, giving molecular bowl/saddle hybrids. In addition, post‐functionalization on the *peri*‐positions endows them with chirality as confirmed by chiral high‐performance liquid chromatography (HPLC) analysis. X‐ray crystallographic analysis demonstrated the diverse molecular geometries of these non‐planar molecular carbons. Kinetic studies on conformational dynamics of the molecular bowl/saddle hybrid clearly revealed both the bowl‐to‐bowl and saddle‐to‐saddle inversion processes via ^1^H nuclear magnetic resonance (NMR) and chiral HPLC analyses. Spectroscopic results indicated these compounds exhibit intriguing photophysical properties and oxidation behaviors. Moreover, this molecular bowl/saddle hybrid showed adaptive geometries and high‐order guest binding to capture three fullerene molecules at the curvatures, which is seldomly reported in nonplanar molecular carbons.

## Results and Discussion

### Synthesis

**Scheme 1:**
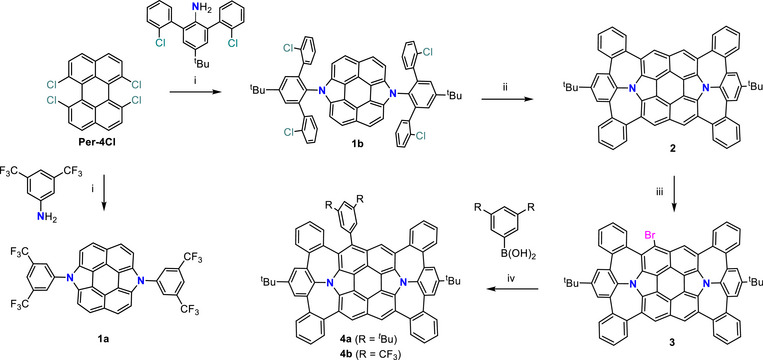
Synthesis of N‐doped nonalternant nanocarbons **1a**/**1b**, **2** and **4a**/**4b**. Reagents and conditions: (i) for **1a**: Pd(P*^t^* Bu_3_)_2_, NaO*^t^* Bu, *o*‐xylene, 120 °C, 24 h, 30%; for **1b**: Pd(P*^t^* Bu_3_)_2_, NaO*^t^* Bu, toluene, 110 °C, 24 h. (ii) Pd(OAc)_2_, PMe*^t^* Bu_2_•HBF_4_, DBU, DMAc, 170 °C, 12 h, 23% in two steps from **Per‐4Cl**. (iii) NBS, chloroform, 0 °C, 3 h, 45%. (iv) Pd(PPh_3_)_4_, K_2_CO_3_, toluene/ethanol/H_2_O, 110 °C, 12 h, 95% for **4a**; 92% for **4b**.

Our previous work has demonstrated that Pd‐catalyzed Buchwald‐Hartwig C‐N coupling reaction of 2,2′‐dihalo‐1,1′‐biphenyl analogs with aniline could effectively construct N‐annulated pentagons.^[^ ^60^, ^61^ ^]^ Thus, we expected that tetrachloroperylene **Per‐4Cl**, prepared from debromination of 3,4,9,10‐tetrabromo‐1,6,7,12‐tetrachloroperylene (see Supporting Information), could serve as ideal starting materials to synthesize double N‐annulated perylene derivatives **1**. By carefully screening the reaction conditions, Buchwald–Hartwig coupling reaction of **Per‐4Cl** with 3,5‐bis(trifluoromethyl)aniline using bis(tri‐*tert*‐butylphosphine)palladium and potassium *tert‐*butoxide in *o*‐xylene at 120 °C for 24 h provided **1a** in 30% isolated yield (Scheme 1). Nevertheless, there are two obstacles in the synthesis of **1b**: one is the large steric hindrance of *ortho‐*chlorophenyl groups, and the other one is the regioselectivity of Buchwald–Hartwig reaction since there are two types of chloro substituents. To our delight, this reaction went smoothly at a lower temperature of 110 °C in toluene although trace amounts of dechlorinated side‐products were formed. Owing to the difficulty in separating dechlorinated species, the mixture was directly used in the subsequent Pd‐catalyzed quadruple C─H/C─Cl coupling reaction, which successfully afforded compound **2** in a 23% yield over two steps. Bromination of **2** with 1 equiv. of N‐bromosuccinimide (NBS) afforded **3** in 45% yield, and subsequent Suzuki coupling reaction with phenylboronic acid gave compound **4a**/**4b** in 95%/92% yields. Notably, compound **4** is chiral owing to the existence of bowl chirality.^[^ ^62^ ^]^ Compounds **1**, **2,** and **4** show good solubility in toluene and are stable under ambient conditions. The molecular structures of compounds **Per‐4Cl**, **1a** and **2** were unambiguously confirmed by X‐ray crystallographic analysis (vide infra),^[^ ^63^ ^]^ NMR spectroscopy, and mass spectrometry (Supporting Information).

### X‐ray Crystallography and Electronic Structure

**Figure 2:**
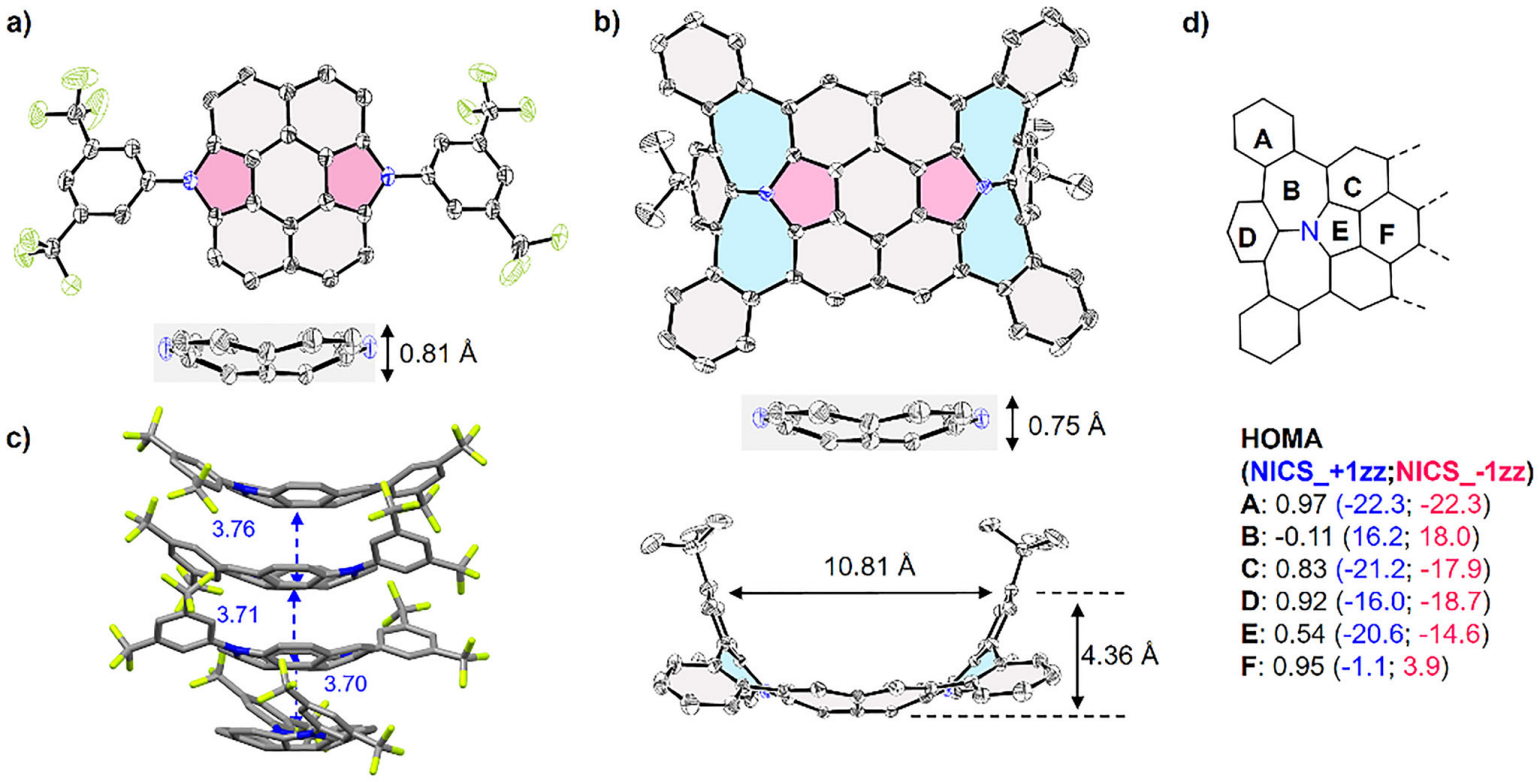
X‐ray structures of a) **1a** and b) **2**. Hydrogen atoms are omitted for clarity. c) Intermolecular interactions of **1a**. d) Calculated HOMA and NICS(1)zz values of **2**. The NICS(1)zz values calculated at 1 Å above the concave surface is in blue color, and those at 1 Å above the convex surface is in red color.

Single crystals suitable for X‐ray diffraction analysis were grown from slow diffusion of methanol into a solution of **Per‐4Cl** in chloroform. Single crystals of **1a** were grown from slow diffusion of acetonitrile into a solution in dichloromethane. Because of the steric hindrance of chloro substituents at the bay‐positions, **Per‐4Cl** exhibits a highly twisted geometry with a dihedral angle of 36.0° at bay positions (Figure S16). Notably, the central ring shows a large torsion of 29.6°, which is comparable to other twisted perylene derivatives.^[^ ^64^, ^65^ ^]^ In the molecular packing, a pair of enantiomers (*P,P* and *M,M*) are observed in each cell with π‐π interactions. Effective dispersion forces between the protons at *peri‐*positions of perylene with the distance of 2.39 Å result in highly ordered 1D chains (Figure S17). X‐ray structure of **1a** reveals a bowl‐shaped geometry with a depth of 0.81 Å (Figure 2a), which is slightly shallower than that of corannulene (0.86 Å) and pure carbon analog (0.90 Å).^[^ ^26^ ^]^ Interestingly, different form the staggered packing mode of carbonaceous congeners,^[^ ^26^ ^]^ **1a** self‐assembled into 1D columns with the distance of ca. 3.7 Å between the central rings along the *b*‐axis (Figure 2c), which is crucial for the potential applications of organic ferroelectrics.^[^ ^66^ ^]^ The adjacent four molecules are rotated by 80°, 55°, and 78°, respectively, to alleviate steric hindrance. The intermolecular interactions are computationally visualized by independent gradient model based on Hirshfeld partition (IGMH) analyses (Figure S21), which indicate that there are significant noncovalent attractive interactions (green surface) among these molecules. Besides, these molecular columns array in antiparallel orientations (Figure S18), forming highly ordered 2D packing by virtue of intermolecular H‐bonding between fluorine and hydrogen atoms.

Slow diffusion of methanol into a solution of **2** in dichloromethane gave single crystals with suitable quality. X‐ray crystallographic analysis reveals the highly curved geometry of **2** as a bowl/saddle hybrid (Figure 2b). Notably, the N‐annulated perylene subunit becomes shallowed by 0.06 Å compared to **1a** upon fusions of two heptalene units. The distance between the tips of the peripheral rings, denoted as “tip−tip parabola”, is 10.8 Å, and the depth, defined as the distance from the peripheral tip to the plane of the central ring, is 4.4 Å, which is among the deepest saddle‐shaped nanographenes.^[^ ^35^, ^40^, ^67^ ^]^ In the solid state, two molecules interlock with the *tert‐*butyl groups occupying the space above the saddle via CH‐π interactions (Figure S19), which is reminiscent of aromatic saddles reported by Miao.^[^ ^68^ ^]^

The aromaticity of these molecules is evaluated by the harmonic oscillator model of aromaticity (HOMA) values based on bond length analyses of the crystal structures. Rings A, C, and D of **2** show strong aromatic character with the values from 0.83 to 0.97 (Figure 2d), while N‐doped seven‐membered ring B exhibits nonaromatic feature with the value of −0.11, and N‐doped pentagon F shows moderate aromatic character with the value of 0.54. These results are in line with the calculated nuclear independent chemical shift (NICS) values and anisotropy of the induced current density (ACID) plots (Figure S25). Notably, rings B, C, E, and F display more negative values in the concave side than those in the convex side. Three‐dimensional isochemical shielding surface (ICSS, Figure S22) calculations revealed that the shielding regions (blue area) largely locate on the concave space of **2**, and the deshielding regions (red area) lie along the peripheral edge. Aromaticity analyses of **1a**, including HOMA values, NICS, ACID, and ICSS calculations, gave similar results to those of **2** (Table S5, Figures S22,S24). These results suggest that the shielding effect of curved nanographenes is stronger in concave space.

### Molecular Dynamics

**Figure 3:**
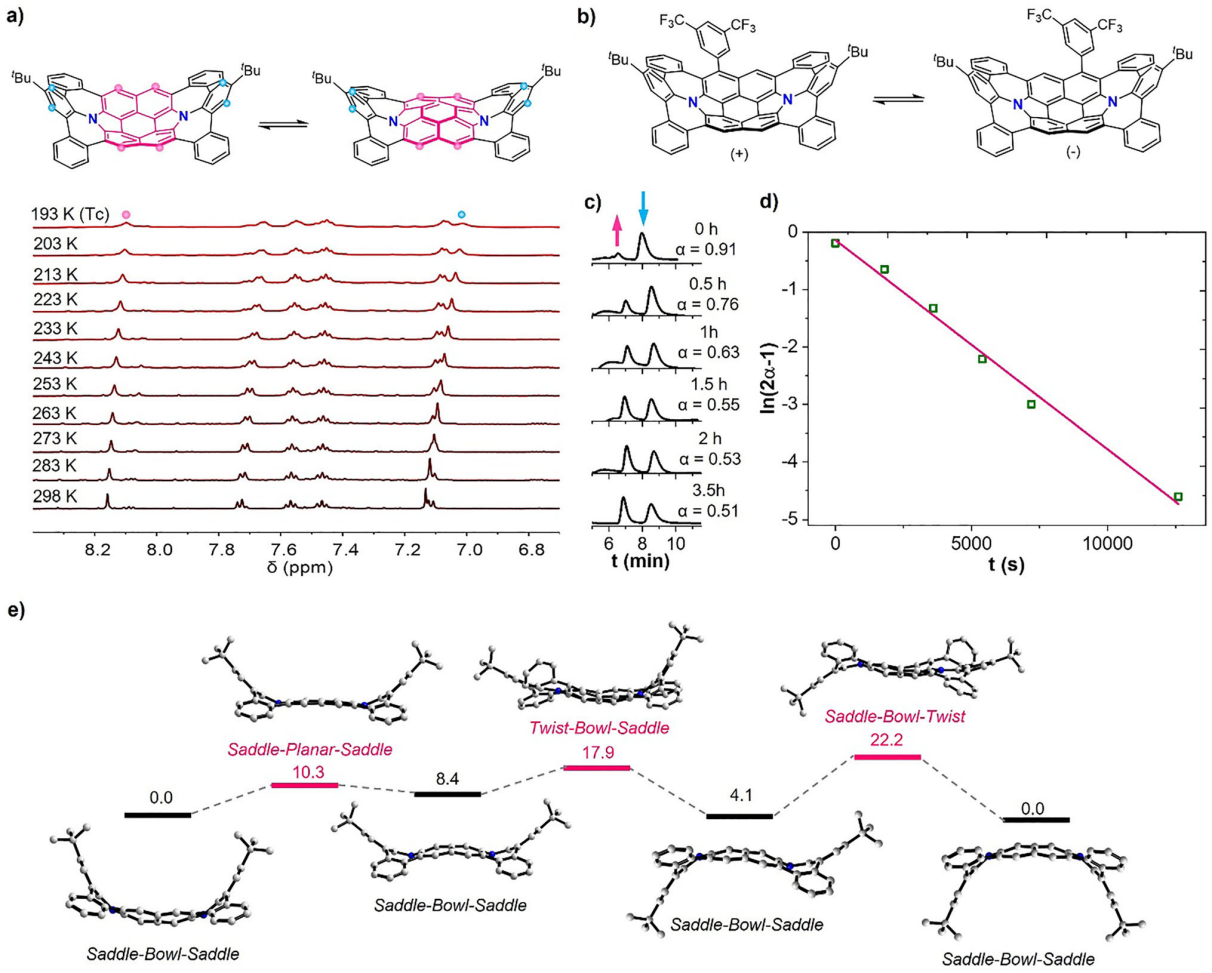
a) VT ^1^H NMR spectra (aromatic region, 500 MHz) of **2** measured in CD_2_Cl_2_, which revealed the bowl‐to‐bowl inversion process of the N‐annulated perylene subunit of **2**. b) The racemization of chiral **4b**. c) Chiral HPLC analyses of enantiopure **4b** with analytic ID column after specific time at room temperature in dichloromethane. α is the conversion ratio of the second fraction. Eluent: *n*‐hexane/dichloromethane = 85:15, v/v; flow rate: 1.0 mL/min; detected by absorption at 254 nm. d) Plot of HPLC‐based conversion ratio (α) of enantiopure **4b** at time t. e) The plausible inversion pathway of **2** with relative Gibbs free energy (kcal mol^−1^) calculated at the B3LYP/6–31G(d) level of theory.

The bowl‐to‐bowl inversion process of **1a** was investigated by variable‐temperature (VT) ^1^H NMR analysis (Figure S7). As the temperature gradually decreased to the instrumental temperature limit of 183 K, the proton signals in the aromatic region became broadened due to the slowing down of the inversion process. Correspondingly, the bowl‐to‐bowl inversion barrier was determined to be ca. 9.2 kcal mol^−1^. This value is slightly larger than the theoretical one of 5.6 kcal mol^−1^ (Figure S26). Similarly, the proton signals of **2** became broadened as the temperature cooled down from 298 to 193 K (Figure 3a). According to the changes of the proton signal at 8.17 ppm, the interconversion rate *k* is estimated to be 33 s^−1^ at 193 K, corresponding to an energy barrier of 9.8 kcal mol^−1^, which is in agreement with the calculated value of the bowl‐to‐bowl inversion (10.3 kcal mol^−1^, Figure 3e). To elucidate the energy barrier of the inversion process of the bowl/saddle‐hybridized structure (Figure 3b), chiral resolution of **4a**/**4b** was investigated by chiral HPLC. Two signals corresponding to two enantiomers of both **4a**/**4b** could be observed at different retention time (Figures S9, S10). Nevertheless, racemization occurred simultaneously at room temperature due to the low energy barrier. Thus, the racemization barrier was estimated by chiral HPLC analysis on the time‐dependent growth of the optically pure enantiomer **4b** at room temperature (Figures 3c,d S11).^[^ ^69^ ^]^ The half‐life time was determined as 63 mins together with the rate constant of 1.82 × 10^−4^ s^−1^. Correspondingly, the Gibbs free energy of activation for racemization was determined to be 22.4 kcal mol^−1^ according to the Eyring equation. This value is significantly higher than that of corannulene (11.5 kcal mol^−1^),^[^ ^70^ ^]^ but lower than that of π‐expanded derivatives (35.7 kcal mol^−1^).^[^ ^46^ ^]^ To further investigate the molecular dynamics of the bowl/saddle hybrid, the plausible inversion pathway of **2** was also predicted by theoretical calculations (Figure 3e), which revealed energies barriers of 10.3, 17.9, and 22.2 kcal mol^−1^, respectively, for bowl‐to‐bowl inversion and saddle‐to‐saddle inversion, which match well with the experimental results. Moreover, the S‐shaped isomer has a higher energy by 4.1 kcal mol^−1^ than that of the saddle‐shaped one, which generally has the lowest energy for bowl‐shaped dimers.^[^ ^71^, ^72^ ^]^

### Photophysical Properties

**Figure 4:**
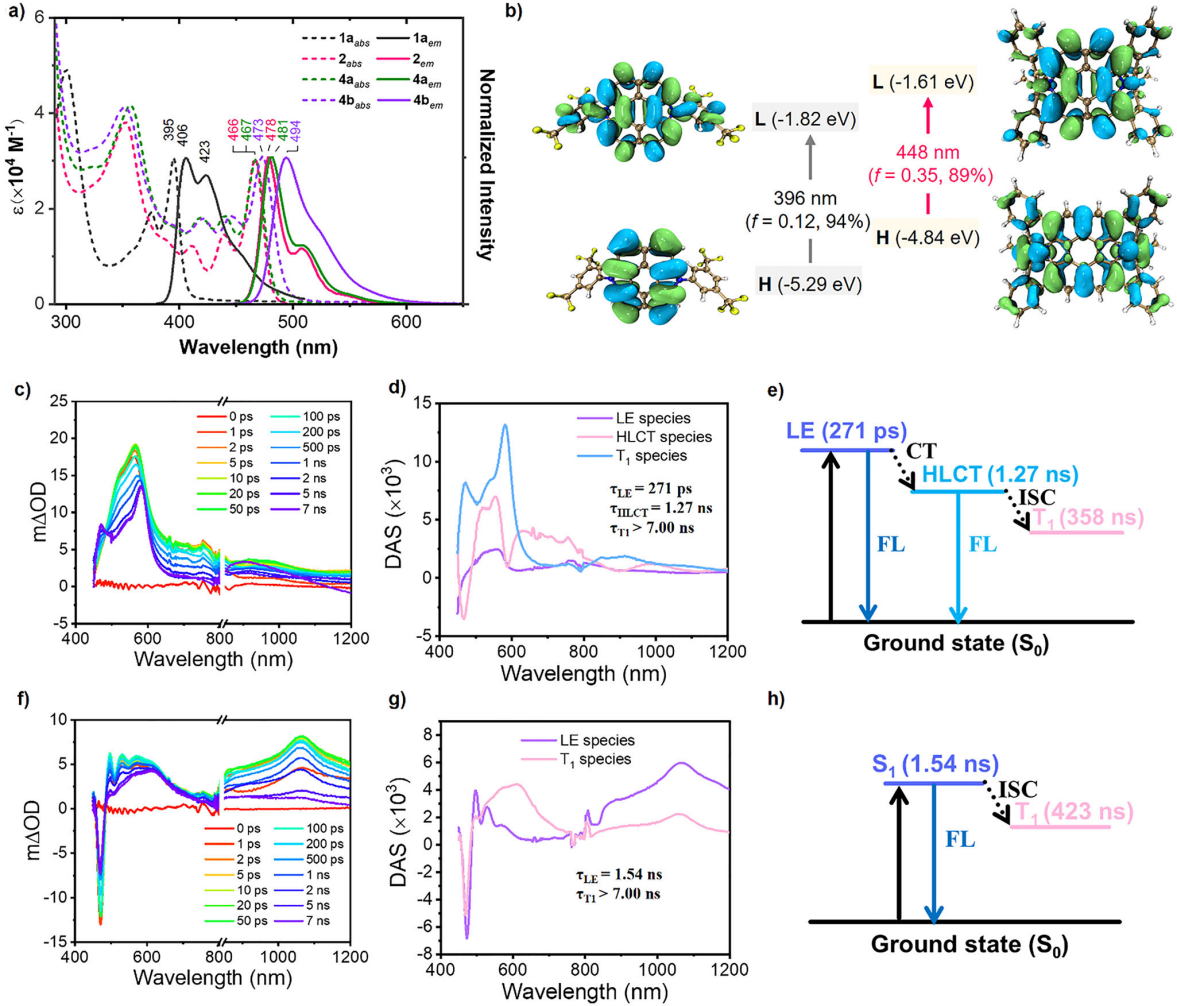
a) Absorption and emission spectra of **1a**, **2**, **4a,** and **4b** measured in dichloromethane. The concentration is 10 µM. b) Molecular frontier orbitals and electronic transitions of **1a** and **2** calculated at the B3LYP/6–31(d) level. c) fs‐TA spectra from 0 ps to 7 ns, d) DAS feature of the transient species by global analysis, and e) photophysical process for **1a** in toluene. f) fs‐TA spectra from 0 ps to 7 ns, g) DAS of the transient species by global analysis, and h) photophysical process for **2** in toluene.

To investigate the electronic properties of these bowl‐shaped or bowl/saddle‐hybrid molecules, UV–vis absorption and photoluminescence were performed in CH_2_Cl_2_ under ambient conditions (Figure 4a). Both **1a** and **2** exhibit well‐defined absorptions with the maximums at 395 and 466 nm, respectively, which can be attributed to the HOMO‐LUMO transitions (Figure 4b, Table S6, S7). The HOMOs of **1a** mainly localize on the doubly N‐annulated perylene skeleton, and the LUMOs further extend to the substituents on the N atoms due to the electron affinity of the 3,5‐bis(trifluoromethyl)phenyl groups, indicating the hybridized local and charge transfer (HLCT) feature of **1a**. For compound **2**, both the HOMOs and LUMOs distribute on the whole molecular skeleton, suggesting a local excited (LE) state feature of **2**. Correspondingly, the optical energy gaps are determined to be 3.02 eV for **1a**, and 2.54 eV for **2**. Introducing one 3,5‐di*‐tert*‐butylphenyl or 3,5‐bis(trifluoromethyl)phenyl group in the *peri‐*position induces slight redshifts of the absorption maximums for **4a** and **4b**. Moreover, these compounds are emissive in solution. **1a** shows a blue emission at 406 nm with a fluorescence quantum yield (*Φ*_F_) of 15%. The fluorescence of **2**, **4a,** and **4b** is redshifted to 478, 481, and 494 nm with the moderate *Φ*_F_ values of 39%, 43%, and 48%, respectively. Notably, **2** and **4a** exhibited ultranarrow emissions with full width at half maximum (FWHM) of 20 nm, which is comparable to boron‐ and/or nitrogen‐doped fluorophores.^[^ ^73^, ^74^, ^75^ ^]^ Moreover, the emissions of compound **1a** and **2** were measured in the solution with different polarity (Figure S4). Compound **2** displays nearly identical emissions in different solvents, while compound **1a** shows pronounced changes on emissions in the solvents with high polarity. Specifically, the emission peak at 420 nm is slightly redshifted to 430 nm, while the one at 406 nm remains unchanged with the decrease of intensity, indicating that the two emission peaks of compound **1a** are originated from the LE state and HLCT state. These results are further supported by the analysis on HOMOs and LUMOs (vide supra).

To explore the multiple‐peak emission feature, time‐correlated single photon counting (TCSPC) technique was used to investigate the lifetime of each emission peaks of **1a** and **2** in toluene (Figures S1, S2). Two emissive excited species were detected at longer wavelengths for **1a**, which suggests it simultaneously has both the LE and HLCT emission features. As for **2**, the emission lifetime showed close values at different wavelengths, indicating the emission originates from the different vibrational energy levels of the S_1_ excited state.

The different emission behaviors between **1a** and **2** in toluene solution were further investigated by the femtosecond/nanosecond transient absorption (fs/ns‐TA) spectroscopy upon 375 nm excitation. The spectra of **1a** showed a main excited‐state absorption (ESA) peak at 565 nm associated with a shoulder peak at 530 nm in several picoseconds (Figure 4c). This ESA signal was gradually redshifted to 582 nm, and a new peak at 470 nm appeared afterward. In NIR region, the ESA peak at 960 nm first decreased, and then increased and shifted to 920 nm. Three decay‐associated spectra (DAS) species were obtained from the global analysis, with the lifetimes *τ*_1_ = 271 ps, *τ*_2 _ = 1.27 ns, and *τ*_3 _> 7.00 ns. As shown in Figure 4d, the negative stimulated emission (SE) peak exhibited a redshift from the first to the second species, indicating transformation from the LE state species to HLCT state species. The undecayed third species was further investigated by ns‐TA spectroscopy, and the lifetime of the long‐lived species was found to be 358 ns, which can be assigned to the triplet species (Figure S3a,b). In the early stage of fs‐TA spectra for **2**, the wavy feature could be observed in the visible region, which results from the overlap between ESA bands and SE peaks at 478, 508, and 550 nm, while a peak at 1080 nm was shown in NIR region (Figure 4f). Subsequently, the wavy band gradually shifted to 610 nm, and the 1080 nm peak almost decayed to the baseline. Global analysis based on the parallel model resulted in two DAS species (Figure 4g), with the time constants of 1.54, and >7.00 ns. The negative SE signal for the first species can match well with the emission peak for **2**, so it can be attributed to the LE state species. The long‐lived triplet state for **2** was confirmed by the ns‐TA spectra, and its lifetime was 423 ns (Figure S3c,d). The transient spectroscopy results unveil that, **1a** shows a simultaneous decay of LE and HLCT species, which resulted in the double‐peak emission, whereas **2** only presents one emissive LE specie.

### Electrochemical Properties and Chemical Oxidations

**Figure 5:**
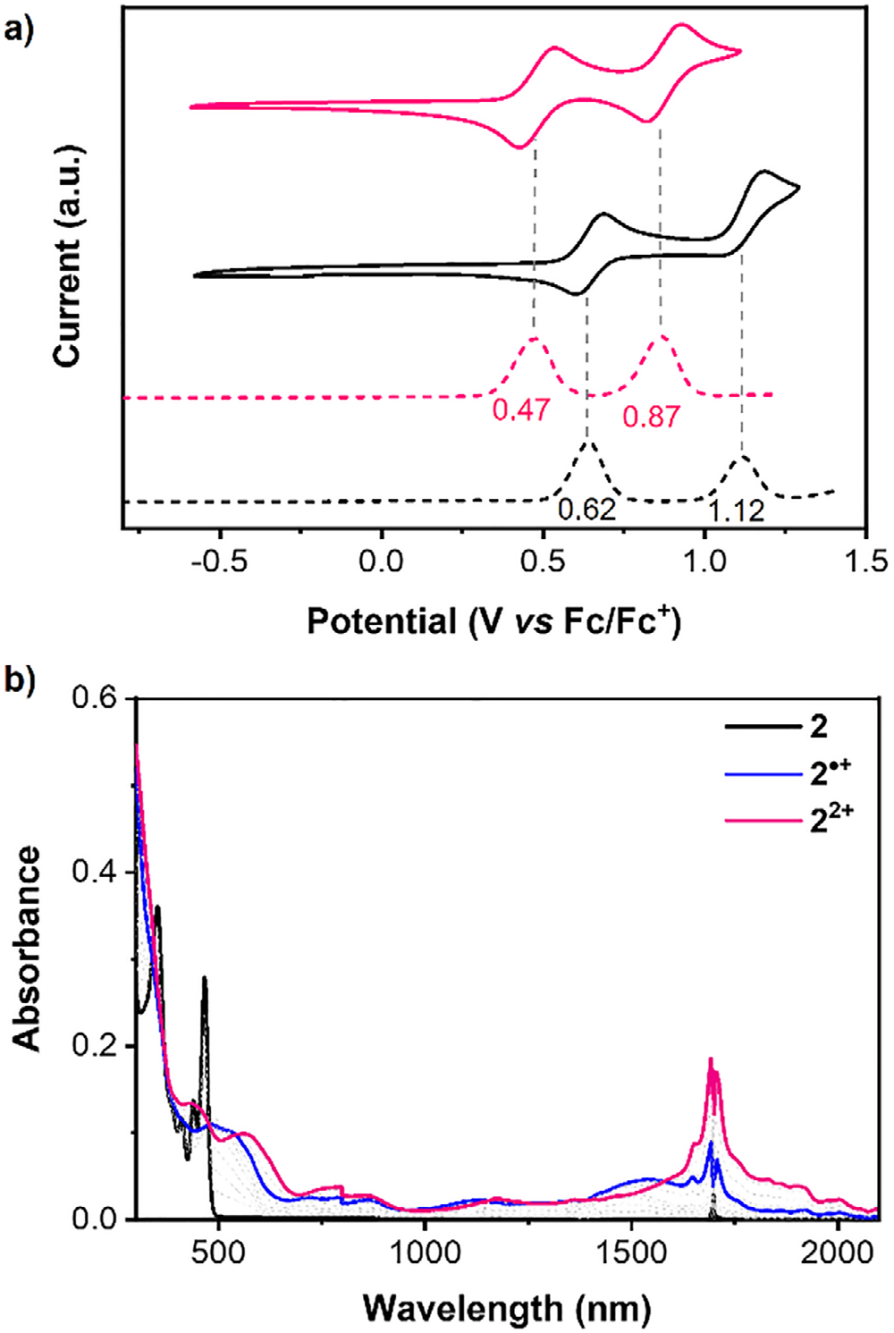
a) Cyclic voltammogram and differential pulse voltammogram of **1a** (black) and **2** (red) measured in DCM with 0.1 M *n*‐Bu_4_N•PF_6_ as supporting electrolyte. The scan rate is 100 mV/s for CV. b) UV–vis‐NIR absorption spectrum of **2** upon chemical titration with NO•SbF_6_ in dry DCM.

Cyclic voltammetry and differential pulse voltammetry measurements (Figure 5a) revealed one reversible oxidation wave with the half‐wave potential *E*_1/2_^ox^ at 0.62 V, and one quasi‐reversible oxidation wave at 1.12 V (versus Fc/Fc^+^) for **1a**, and two reversible oxidation waves at 0.47 and 0.87 V (versus Fc/Fc^+^) for **2**. The well‐defined redox reversibility implies the good stability of the oxidized species, which provides opportunities to study their optical properties. The HOMO energy levels of **1a** and **2** were determined to be −5.38 and −5.21 eV, respectively, based on the onset potential, which indicate the electron‐donating character of both compounds.

To give insight of the radical cation and dication species, chemical oxidations of **1a** and **2** were performed by careful titration with nitrosonium hexafluoroantimonate (NO•SbF_6_, Figures 5b, S5) in dichloromethane. By gradually increasing the amount of the oxidant, **1a^•+^** was formed as confirmed by electron paramagnetic resonance (EPR) spectroscopy (Figure S6), and exhibited a broad absorption with the maximum at 453 nm in the range of 370–560 nm. Further oxidation was not carried out due to the limited reduction potential of the oxidant. In contrast, the radical cation and dication of **2** show broad long‐wavelength absorption in the NIR region extending to 2100 nm. The aromaticity of **1a^2+^** and **2^2+^** was theoretically investigated by NICS values and ACID plots (Table S5, Figures S24, S25). The peripheral benzene and five‐membered rings of **1a^2+^** display large positive NICS(1)zz values, indicating strong antiaromatic character. In addition, the AICD plot reveals counterclockwise (paratropic) ring current flow along the periphery, which is consistent with the reported results for the dicyclopentaperylene derivatives.^[^ ^26^ ^]^ For **2^2+^**, the peripheral benzene rings remain aromatic as that of the neutral structure, while the N‐annulated perylene core becomes to be antiaromatic, and the seven‐membered rings convert to be nonaromatic. These results are further supported by calculated ACID plots.

### Supramolecular Interactions with Fullerenes

**Figure 6:**
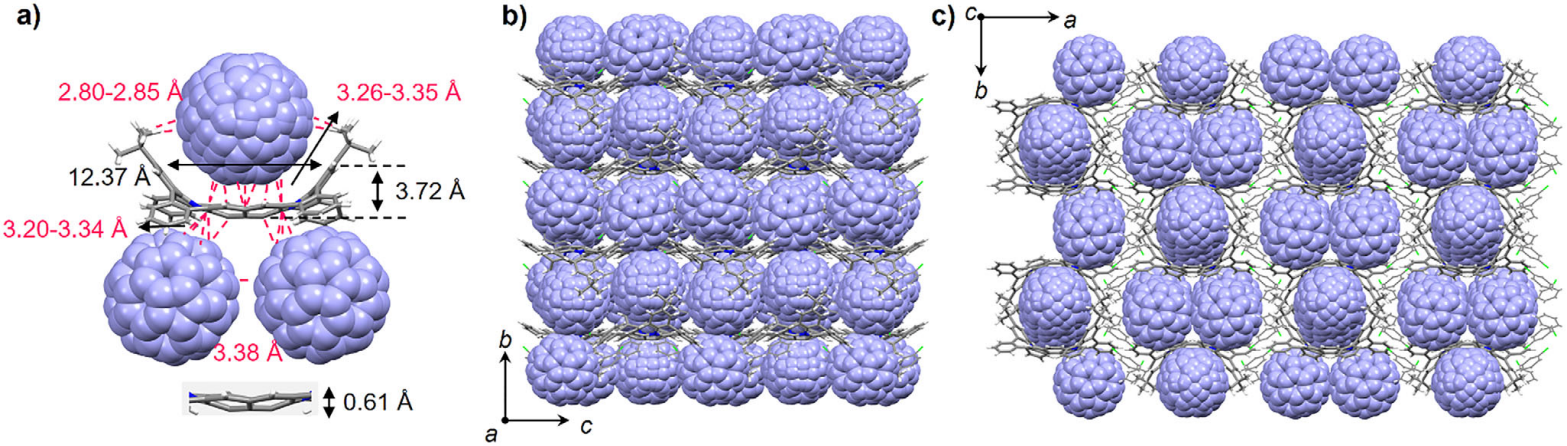
a) X‐ray structure of the complex **2@C_60_**. Supramolecular assembly of **2** and C_60_ molecules along b) *a* and c) *c* axis. Solvent molecules are also shown.

By virtue of the curved π‐surface that holds potential in recognition of fullerenes via concave–convex interactions, the supramolecular behaviors of **2** with fullerene C_60_ and C_70_ were investigated in both solution and solid state. Naturally, a stoichiometry ratio of 1:3 for **2** and fullerene molecules is expected since there are three concaves, that are one bowl‐shaped subunit and two saddles, in **2**. It is worth to note that although quantitative analyses on the key parameters, including stoichiometry ratio and binding constant, have been well developed in supramolecular systems of the H/G = 1:2, 1:1, or 2:1 models by using spectroscopic techniques, such as NMR, UV–vis absorptions, and fluorescence,^[^ ^76^ ^]^ the investigation of the supramolecular interactions of multiple host–guest complexes (H/G = 1:n, *n* ≥ 3) remains difficult.^[^ ^77^, ^78^, ^79^ ^]^ Titration of **2** with fullerene C_60_ or C_70_ in toluene was conducted by monitoring the emission intensity changes at 478 and 505 nm (Figure S12), and the data were well‐fitted to a 1:1 binding model. The binding constant (*K*_a_) for the complex of **2** and C_60_ was determined to be 1.54 × 10^4^ M^−1^, while the *K*_a_ value for **2** and C_70_ (*K*_a_ = 4.10 × 10^4^ M^−1^) was pronouncedly increased probably due to the larger π‐surface and higher electron affinity of C_70_ over C_60_. The supramolecular interactions with fullerene were further investigated by the crystal structure of the complex. Slow diffusion of methanol into the chlorobenzene solution successfully gave single crystals of the complex **2@C_60_**. The crystal structure revealed a 1:3 stoichiometry of **2** and C_60_ molecules (Figure 6a), in which one C_60_ molecule locates above the concave surface of the N‐annulated perylene core, and the other two C_60_ molecules interact with the saddle surfaces of the N‐doped dibenzoheptalene subunits. The shortest distances between **2** and three C60 molecules are in the range of 2.97 to 3.35 Å, indicating effective π‐π interactions. Distinct geometric changes of **2** were observed upon complexation. The width of the “tip–tip parabola” was elongated by 1.56 Å, and the depths of both the bowl subunit and the whole saddle skeleton became shallowed by 0.14 and 0.64 Å, respectively, compared to unassembled **2** molecules (vide supra). These results indicate that **2** could act as a conformation‐adaptive host for molecular recognitions. Notably, though the supramolecular chemistry with fullerenes has been well developed for nonplanar π‐systems, such as carbon nanohoops,^[^ ^80^ ^]^ nanobelts,^[^ ^81^, ^82^ ^]^ bowls,^[^ ^30^, ^83^ ^]^ saddles^[^ ^35^, ^40^ ^]^ and cages,^[^ ^84^, ^85^ ^]^ and adaptive molecular carbons allowing for multiple guest bindings (≥3), especially for small size molecules like **2** with a contorted framework of 56 sp^2^ carbon atoms and two nitrogen atoms, remain rarely reported. Moreover, the molecular arrangements reveal alternating stacks of compound **2** and C_60_ in a 1:3 ratio, which assemble into a continuous 1D structure stabilized by effective intermolecular π‐π interactions between **2** and C_60_ (Figure S20). These arrays assembled into highly ordered lamellar structures with C_60_ molecules encapsulated in the cavities via tight π‐π interactions (Figure 6b,c). Consequently, the resultant assembled structures might enable effective charge transfer, which is crucial for applications in organic electronics.^[^ ^40^, ^86^, ^87^ ^]^

## Conclusion

In summary, N‐doped molecular bowl/saddle hybrids with nonalternant topologies were synthesized by an effective core‐expansion strategy via successive Pd‐catalyzed annulations from twisted tetrachloroperylene. X‐ray structures revealed the bowl‐shaped geometry for the N‐annulated perylene intermediate **1**, and the bowl/saddle hybrid structure for **2**. Notably, the resultant hybrid exhibited unique molecular dynamics including both bowl‐to‐bowl and saddle‐to‐saddle inversions, which have been detailed studied by VT ^1^H NMR and chiral HPLC analyses, as well as supported by theoretical calculations. Spectroscopic measurements suggested these molecules exhibited narrow emissions (FWHM = 20 nm for **2** and **4a**) with moderate *Φ*_F_ values. The electron‐rich character and the good redox reversibility allowed chemical oxidation to the formation of radical cation and dication species, which showed broad absorption extending into NIR regions. Moreover, this bowl/saddle hybrid could adaptively interact with fullerenes and multiply bind up to three fullerene guests by virtue of the rich curved π‐surfaces, and assembled into lamellar 3D networks. Our study provides rare examples of curved molecular carbons integrating both bowl‐ and saddle‐shaped subunits, and paves way to molecular design for chiral applications and organic electronics in the future.

## Conflict of Interests

The authors declare no conflict of interest.

## Supporting information



Supporting Information

Supporting Information

## Data Availability

The data that support the findings of this study are available in the Supporting Information of this article.
