# Supplementary material for: N‐Doped Nonalternant Molecular Bowl/Saddle Hybrids
Source: Angew Chem Int Ed Engl. 2025 Sep 30;64(48):e202516881. doi: 10.1002/anie.202516881 (PMC12643336; doi:10.1002/anie.202516881)
Supplement: Supplementary file 1 — Supporting Information [file ANIE-64-e202516881-s002.docx]

Supporting Information

N-Doped Nonalternant Molecular Bowl/Saddle Hybrids

Shuhai Qiu,^[a]^ Kai Chen,^[b]^ Ziqi Deng,^[c]^ Xuan Jin,^[d]^ Zuoyu Li,^[e]^ Guogang Liu,^[d]^ Li Zhang,^[d]^ Wei Jiang,^[b]^ Teng-Teng Chen,^[c]^ Junzhi Liu,*^[a,f,g]^ and Zhaohui Wang*^[e]^

^[a]^Department of Chemistry, The University of Hong Kong, Pokfulam Road, Hong Kong, China

^[b]^School of Chemical Sciences, University of Chinese Academy of Sciences, Beijing 100049, China

^[c]^Department of Chemistry, The Hong Kong University of Science and Technology, Hong Kong, China

^[d]^Key Laboratory for Advanced Materials and Joint International Research Laboratory of Precision Chemistry and Molecular Engineering, Feringa Nobel Prize Scientist Joint Research Center, Frontiers Science Center for Materiobiology and Dynamic Chemistry, Institute of Fine Chemicals, School of Chemistry and Molecular Engineering, East China University of Science and Technology, Shanghai 200237, China

^[e]^Key Laboratory of Organic Optoelectronics and Molecular Engineering, Department of Chemistry, Tsinghua University, Beijing 100084, China

^[f]^State Key Laboratory of Synthetic Chemistry, HKU-CAS Joint Laboratory on New Materials and Shanghai-Hong Kong Joint Laboratory on Chemical Synthesis, The University of Hong Kong, Pokfulam Road, Hong Kong, China.

^[g]^Materials Innovation Institute for Life Sciences and Energy (MILES), HKU-SIRI, Shenzhen, China.

E-mail: wangzhaohui@tsinghua.edu.cn; juliu@hku.hk

**Table of contents**

[**1.** **Materials and Measurements** S2](#_Toc207110966)

[**2.** **Synthetic Details** S4](#_Toc207110967)

[**3.** **Photophysical Properties** S8](#_Toc207110968)

[**4.** **Variable Temperature ^1^H NMR Spectra** S12](#_Toc207110969)

[**5.** **Chiral HPLC Chromatogram** S14](#_Toc207110970)

[**6.** **Host-guest Interactions with Fullerenes** S18](#_Toc207110971)

[**7.** **X-ray Crystal Structures** S19](#_Toc207110972)

[**8.** **Theoretical Calculations** S26](#_Toc207110973)

[**9.** **^1^H and ^13^C NMR Spectra** S33](#_Toc207110974)

[**10.** **Mass Spectra** S42](#_Toc207110975)

[**11.** **References** S45](#_Toc207110976)

1. **Materials and Measurements**

Solvents were purified and dried by standard methods prior to use. All commercially available reagents were used without further purification unless otherwise noted. Column chromatography was generally performed on silica gel (200 - 300 mesh) and reactions were monitored by thin layer chromatography (TLC) using silica gel GF254 plates with UV light to visualize the course of reaction. The ^1^H, ^13^C and 2D NMR data were recorded on a 500 MHz or 600 MHz spectrometer using DMSO-*d6*, CD_2_Cl_2_ as solvent. All chemical shifts are quoted in ppm, relative to tetramethylsilane, using residual solvent peak as a reference standard. The following abbreviations were used to explain the multiplicities: s = singlet, d = doublet, m = multiplet. Mass spectra were obtained on a Bruker Q-Tof Maxis II mass spectrometer and MALDI-TOF MS system, Bruker ultrafleXtreme. The UV-*vis* spectroscopy was performed on the Agilent Cary 60. The photoluminescence spectra, quantum yields and fluorescence lifetime were conducted on Edinburgh FLS1000 (Edinburgh Instruments Ltd, UK). Cyclic voltammetry (CV) measurements were carried out on a CHI660E (CH Instruments, USA) in a three-electrode cell in an anhydrous dichloromethane (DCM) solution of tetrabutylammonium hexafluorophosphate (*n-*Bu_4_NPF_6_, 0.1 M) with a scan rate of 100 mV/s at room temperature. Electron paramagnetic resonance (EPR) spectra were measured on a Bruker ESP EMX-8/2.7 spectrometer. All potentials were further calibrated against ferrocene/ferrocenium (Fc/Fc^+^). Single crystals data collections were performed on a Bruker D8 Venture with a CuKα (*λ* = 1.5406 Å) or GaKα (λ = 1.34139) X-ray source. All calculations were performed using the SHELXL and the crystal structure crystallographic software package.

The fs-TA measurements were conducted using the Helios pump-probe transient absorption spectrometer system (Ultrafast Systems, USA) with the femtosecond laser from the Astrella USP regenerative amplified Ti:sapphire laser system (Coherent, USA). The 800-nm laser light with 34-fs pulse width was subsequently split into two beams, one as the pump beam and another one as the probe beam. The pump beam passed through an Apollo-T optical parametric amplifier (Ultrafast Systems, USA) to generate the 370-nm pump beam, whereas the probe beam passed through a sapphire crystal or a YAG crystal to generate a white-light continuum (450–800 nm) or a NIR continuum (900-1500 nm). The time-delayed probe beam was controlled by the optical delay rail with a maximum temporal delay at 8 ns. Two beams pass through the samples and the signals were then collected by the detector. The sample solutions with a concentration of 1×10^-4^ M in toluene were measured in a 2-mm path-length quartz cuvette at room temperature. The spectrometric data were recorded two times for each sample in a 3D wavelength-time-absorbance matrix. The subtraction of background, the subtraction of scattering light, and chirp correction were done for all the data before analysis. The principal components were ensured by singular value decomposition (SVD), and then the global fitting was carried out with the selected principal components and exponential function using the sequential kinetics scheme based on the fs-TA spectra by Glotaran 1.5.1.

The ns-TA spectra were measured by the HARPIA-TA-FP pump-probe transient absorption system (Light Conversion, Lithuania). The 1030-nm laser was generated by the PHAROS modular-design femtosecond laser system and passed through the ORPHEUS collinear OPA system (Light Conversion, Lithuania) to generate the 370-nm pump beam. The time-delayed broadband nanosecond probe beam was generated by a electronically triggered photonic crystal fiber (PCF) laser system. Two beams pass through the samples and the signals were then collected by the detector. The sample solutions with a concentration of 1×10^-4^ M in toluene were measured in a 2-mm path-length quartz cuvette at room temperature. The spectrometric data were recorded three times for each sample in a 3D wavelength-time-absorbance matrix. The subtraction of background and the subtraction of scattering light were done for all the data before analysis. The single-wavelength kinetic fitting was conducted using a double-exponential function by Carpetview 3.0.1.

Data-fitting and association constant values were obtained based on fluorescence titration using the BindFit program developed by Prof. Pall Thordarson of UNSW.^S1-S2^ This program is freely available online via the following link: http://supramolecular.org.

1. **Synthetic Details**

**Synthesis of Per-4Cl**

A Schlenk flask was charged with **Per-4Cl-4Br**^S3^ (710 mg, 1 mmol), Pd(OAc)_2_ (34 mg, 0.15 mmol, 15 mol%), PPh_3_ (79 mg, 0.3 mmol, 20 mol%), K_2_CO_3_ (1.1 g, 8 mmol, 8 eq) and *n*-butanol (10 mL) under argon. The mixture was heated to 120 ºC with vigorous stirring for 24 h. The mixture was cooled and poured into methanol, and the precipitate was collected by vacuum filtration, washed with methanol, dried, and purified by flash column chromatography on silica gel, eluted with petroleum ether to afford **Per-4Cl** as yellow solid (254 mg, 65% yield). ^1^H NMR (600 MHz, CD_2_Cl_2_): *δ* 7.83 (d, *J* = 8.6 Hz, 4H), 7.62 (d, *J* = 8.6 Hz, 4H). ^13^C NMR (150 MHz, CD_2_Cl_2_): *δ* 134.3, 133.4, 129.7, 129.1, 128.4, 125.5. MS (MALDI-TOF, 100%): m/z calcd (%) for C_20_H_8_Cl_4_: 389.9347, found: 389.9673.

**Synthesis of** **5'-(*tert-*butyl)-2,2''-dichloro-[1,1':3',1''-terphenyl]-2'-amine**

To a solution of 2,6-dibromo-4-(*tert-*butyl)aniline (921 mg, 3.0 mmol, 1 eq), 2-chlorophenylboronic acid (1.2 g, 7.5 mmol, 2.5 eq), K_2_CO_3_ (2.5 g, 18.0 mmol, 6 eq) in dioxane (30 mL) and water (10 mL), bis(diphenylphosphino)ferrocene)palladium(II) dichloride (Pd(dppf)_2_Cl_2_, 220 mg, 10 mol%) was added under *N*_2_ atmosphere. The mixture was heated at 105 °C overnight. Upon completion, the solution was filtrated and the filtrate was evaporated to dryness. The residue was purified by the column chromatography on silica gel (petroleum ether/CH_2_Cl_2_ = 2/1) to afford compound 5'-(*tert-*butyl)-2,2''-dichloro-[1,1':3',1''-terphenyl]-2'-amine (1045 mg) as a white solid in 94% yield. ^1^H NMR (500 MHz, DMSO-*d6*): *δ* 7.62 - 7.55 (m, 2H), 7.46 - 7.38 (m, 6H), 6.98 (s, 2H), 3.75 (d, *J* = 11.5 Hz, 2H), 1.25 (s, 9H). ^13^C NMR (125 MHz, DMSO-*d6*): *δ* 139.5, 139.4, 138.3, 138.2, 138.2, 138.1, 133.0, 132.9, 132.2, 132.1, 129.8, 129.8, 129.2, 127.6, 127.6, 126.7, 126.6, 124.1, 123.9, 33.6, 31.4. MS (MALDI-TOF, 100%): m/z calcd (%) for C_22_H_21_Cl_2_N: 369.1046, found: 369.1561.

**Synthesis of 1a**

To a mixture of **Per-4Cl** (200 mg, 0.5 mmol, 1 eq), Pd(P*^t^*Bu_3_)_2_ (50 mg, 20mol%) and NaO*^t^*Bu (288 mg, 3.0 mmol, 6 eq), 3,5-bis(trifluoromethyl)aniline ( 240 mg, 1.0 mmol, 2.1 eq) and *o*-xylene (7 mL) was added under *N*_2_ atmosphere. The mixture was heated at 120 °C for 24 h. Upon completion, the solution was filtrated and the filtrate was evaporated to dryness. The residue was purified by the column chromatography on silica gel (petroleum ether/CH_2_Cl_2_ = 3/1) to afford **1a** (108 mg) as a white solid in 30% yield. ^1^H NMR (500 MHz, CD_2_Cl_2_): *δ* 8.31 (s, 4H), 8.02 (d, *J* = 9.0 Hz, 4H), 7.98 (s, 2H), 7.80 (d, *J* = 9.0 Hz, 4H). ^19^F NMR (471 MHz, CD_2_Cl_2_): *δ* -63.25. ^13^C NMR (125 MHz, CD_2_Cl_2_): *δ* 142.0, 140.4, 134.3, 134.1, 133.9, 133.7, 130.3, 129.5, 128.7, 127.9, 127.4, 126.8, 126.4, 125.8, 124.6, 124.5, 124.5, 124.4, 124.4, 122.8, 120.9, 120.5, 120.4, 120.4, 114.1. MS (MALDI-TOF, 100%): m/z calcd (%) for C_36_H_14_F_12_N_2_: 702.0960, found: 702.1354.

**Synthesis of 2**

To a mixture of **Per-4Cl** (206 mg, 0.53 mmol, 1 eq), 5'-(tert-butyl)-2,2''-dichloro-[1,1':3',1''-terphenyl]-2'-amine (400 mg, 1.08 mmol, 2 eq), Pd(P*^t^*Bu_3_)_2_ (50 mg, 20mol%) and NaO*^t^*Bu (300 mg, 3.1 mmol, 6 eq), dry toluene (7.5 mL) was added under *N*_2_ atmosphere. The mixture was heated at 110 °C for 24 h. Upon completion, the solution was filtrated and the filtrate was evaporated to dryness. The residue was purified by the column chromatography on silica gel (petroleum ether/CH_2_Cl_2_ = 3/1) to afford **1b** as a reddish-yellow solid, which was directly used in the next step. To the aforementioned **1b** in dry dimethylacetamide (10 mL), Pd(OAc)_2_ (60 mg, 0.27 mmol), PMe*^t^*Bu_2_•HBF_4_ (140 mg, 0.54 mmol) and DBU (0.6 mL) were added under *N*_2_ atmosphere. The mixture was then heated at 170 ˚C for 12 h. Upon completion, the solution was added to water (50 mL), and the precipitate was collected and purified by the column chromatography on silica gel (*n*-hexane/CH_2_Cl_2_ = 2/1) to afford **2** as a yellow solid in a yield of 23% over two steps. ^1^H NMR (500 MHz, CD_2_Cl_2_): *δ* 8.16 (s, 4H), 7.73 (d, *J* = 7.6 Hz, 4H), 7.56 (t, *J* = 7.5 Hz, 4H), 7.46 (*t*, J = 7.6 Hz, 4H), 7.11 (d, *J* = 8.9 Hz, 8H), 1.17 (s, 18H). ^13^C NMR (125 MHz, CD_2_Cl_2_): *δ* 148.5, 143.9, 143.7, 142.3, 139.7, 134.7, 131.5, 131.2, 129.5, 129.4, 129.2, 128.8, 127.6, 127.6, 127.5, 122.9, 34.3, 31.3. MS (MALDI-TOF, 100%): m/z calcd (%) for C_64_H_42_N_2_: 838.3343, found: 838.4650.

**Synthesis of 3**

To a solution of **2** (74 mg, 0.09 mmol, 1 eq) in chloroform (10 mL), *N*-bromosuccinimide (17 mg, 0.1 mmol, 1.1 eq) were added in portions. The mixture was stirring in dark at 0 ˚C for 3 h. Upon completion, the solvent was evaporated to dryness. The residue was purified by the column chromatography on silica gel (*n*-hexane/CH_2_Cl_2_ = 3/1) to afford compound **3** (37 mg) as a yellow powder in 45% yield. ^1^H NMR (500 MHz, CD_2_Cl_2_): *δ* 8.32 (s, 1H), 8.17 (d, *J* = 10.4 Hz, 2H), 8.07 (d, *J* = 7.8 Hz, 1H), 7.79 (d, *J* = 7.5 Hz, 1H), 7.76 - 7.70 (m, 2H), 7.61 - 7.53 (m, 4H), 7.47 (dt, *J* = 17.3, 7.9 Hz, 4H), 7.31 - 7.28 (m, 1H), 7.13 (dd, *J* = 11.9, 4.2 Hz, 5H), 7.10 - 7.04 (m, 2H), 1.20 (d, *J* = 8.3 Hz, 18H). ^13^C NMR (150 MHz, CD_2_Cl_2_/CS_2_): *δ* 148.6, 148.3, 145.3, 144.2, 143.98, 143.92, 143.88, 143.6, 142.17, 142.15, 142.12, 139.6, 139.40, 139.37, 137.8, 134.76, 134.71, 134.68, 134.66, 132.8, 132.3, 131.8, 131.6, 131.31, 131.29, 130.75, 130.67, 129.9, 129.8, 129.64, 129.61, 129.59, 129.55, 129.4, 129.09, 129.06, 129.0, 128.93, 128.91, 128.3, 128.1, 127.87, 127.85, 127.54, 127.52, 127.46, 127.40, 127.38, 126.89, 126.6, 124.0, 123.8, 123.4, 117.2, 34.33, 34.30, 31.41, 31.38. MS (MALDI-TOF, 100%): m/z calcd (%) for C_64_H_41_BrN_2_: 918.2445, found: 918.4223.

**Synthesis of 4a/4b**

To a solution of **3** (92 mg, 0.1 mmol, 1 eq), (3,5-di-*tert*-butylphenyl)boronic acid (0.2 mmol, 2 eq) or (3,5-bis(trifluoromethyl)phenyl)boronic acid (0.2 mmol, 2 eq), and potassium carbonate (K_2_CO_3_, 55 mg, 0.4 mmol, 4 eq) in a mixture of toluene (10 mL), ethanol (1 mL) and water (1 mL), Pd(PPh_3_)_4_ (23 mg, 20 mol%) was added under *N*_2_ atmosphere. The mixture was heated at 110 °C for 12 h. Upon completion, the solution was evaporated to dryness. The residue was purified by the column chromatography on silica gel (*n*-hexane/CH_2_Cl_2_ = 5/1) to afford compound **4a** (97 mg) or **4b** (77 mg) as a yellow solid in 95% or 92% yield, respectively.

**4a**: ^1^H NMR (600 MHz, CD_2_Cl_2_): *δ* 8.22 (t, *J* = 1.7 Hz, 1H), 8.14 - 8.07 (m, 3H), 7.75 (dd, *J* = 7.6, 1.4 Hz, 1H), 7.69 (dd, *J* = 7.7, 1.4 Hz, 1H), 7.55 (dt, *J* = 7.5, 1.8 Hz, 3H), 7.47 - 7.40 (m, 3H), 7.36 (t, *J* = 1.9 Hz, 1H), 7.31 (s, 1H), 7.19 (dd, *J* = 9.7, 2.0 Hz, 2H), 7.10 (d, *J* = 2.6 Hz, 1H), 7.07 - 7.00 (m, 3H), 6.98 - 6.93 (m, 3H), 6.90 (dd, *J* = 7.8, 1.3 Hz, 1H), 6.85 (dt, *J* = 3.4, 2.1 Hz, 2H), 1.60 (s, 9H), 1.14 (s, 9H), 0.99 (s, 9H), 0.85 (s, 9H). ^13^C NMR (150 MHz, CD_2_Cl_2_): *δ* 151.4, 151.3, 148.3, 148.2, 146.6, 144.5, 144.3, 144.0, 143.8, 143.7, 142.6, 142.3, 142.2, 142.1, 139.8, 139.7, 139.6, 139.5, 139.4, 138.6, 134.6, 134.4, 134.4, 133.6, 133.0, 131.5, 131.4, 131.2, 131.0, 130.9, 130.1, 129.7, 129.6, 129.6, 129.4, 129.4, 129.3, 129.3, 129.2, 129.2, 129.2, 128.7, 128.7, 128.6, 128.3, 128.3, 127.9, 127.9, 127.9, 127.8, 127.7, 127.4, 127.3, 127.1, 126.9, 126.5, 124.1, 123.5, 123.0, 121.0, 35.6, 34.9, 34.1, 33.9, 32.1, 31.3, 31.2, 30.9. MS (MALDI-TOF): [M]^+^ calcd for C_78_H_62_N_2_: 1026.4908, found 1026.6709.

**4b**: ^1^H NMR (500 MHz, CD_2_Cl_2_): *δ* 8.87 (s, 1H), 8.18 (d, *J* = 4.8 Hz, 2H), 7.89 (s, 1H), 7.83 (s, 1H), 7.76 (d, *J* = 7.5 Hz, 1H), 7.72 (d, *J* = 7.6 Hz, 1H), 7.51 (ddt, *J* = 46.1, 23.8, 7.8 Hz, 8H), 7.28 (t, *J* = 4.9 Hz, 2H), 7.26 – 7.21 (m, 1H), 7.17 (d, *J* = 2.0 Hz, 1H), 7.08 (dt, *J* = 24.5, 6.8 Hz, 6H), 6.84 (d, *J* = 7.8 Hz, 1H), 1.24 (s, 9H), 1.14 (s, 9H). ^19^F NMR (471 MHz, CD_2_Cl_2_): *δ* -62.84, -63.76. ^13^C NMR (150 MHz, CD_2_Cl_2_): *δ* 148.6, 148.5, 145.5, 144.3, 144.2, 144.0, 143.9, 143.6, 143.0, 142.7, 142.2, 142.2, 142.1, 139.6, 139.3, 139.2, 137.5, 134.7, 134.6, 134.6, 134.5, 134.2, 134.2, 134.2, 133.6, 133.3, 133.0, 132.9, 132.9, 132.4, 132.3, 132.2, 132.1, 131.9, 131.6, 131.6, 131.3, 131.1, 130.8, 129.9, 129.7, 129.6, 129.6, 129.5, 129.5, 129.4, 129.4, 129.3, 129.0, 128.9, 128.9, 128.7, 128.3, 128.3, 128.1, 127.9, 127.7, 127.7, 127.6, 127.6, 127.5, 127.3, 125.8, 125.2, 124.6, 124.0, 123.4, 123.3, 122.8, 122.0, 121.3, 121.3, 121.2, 34.3, 34.2, 31.3, 31.2. MS (MALDI-TOF): [M]^+^ calcd for C_72_H_44_F_6_N_2_: 1050.3403, found 1050.5495.

1. **Photophysical Properties**


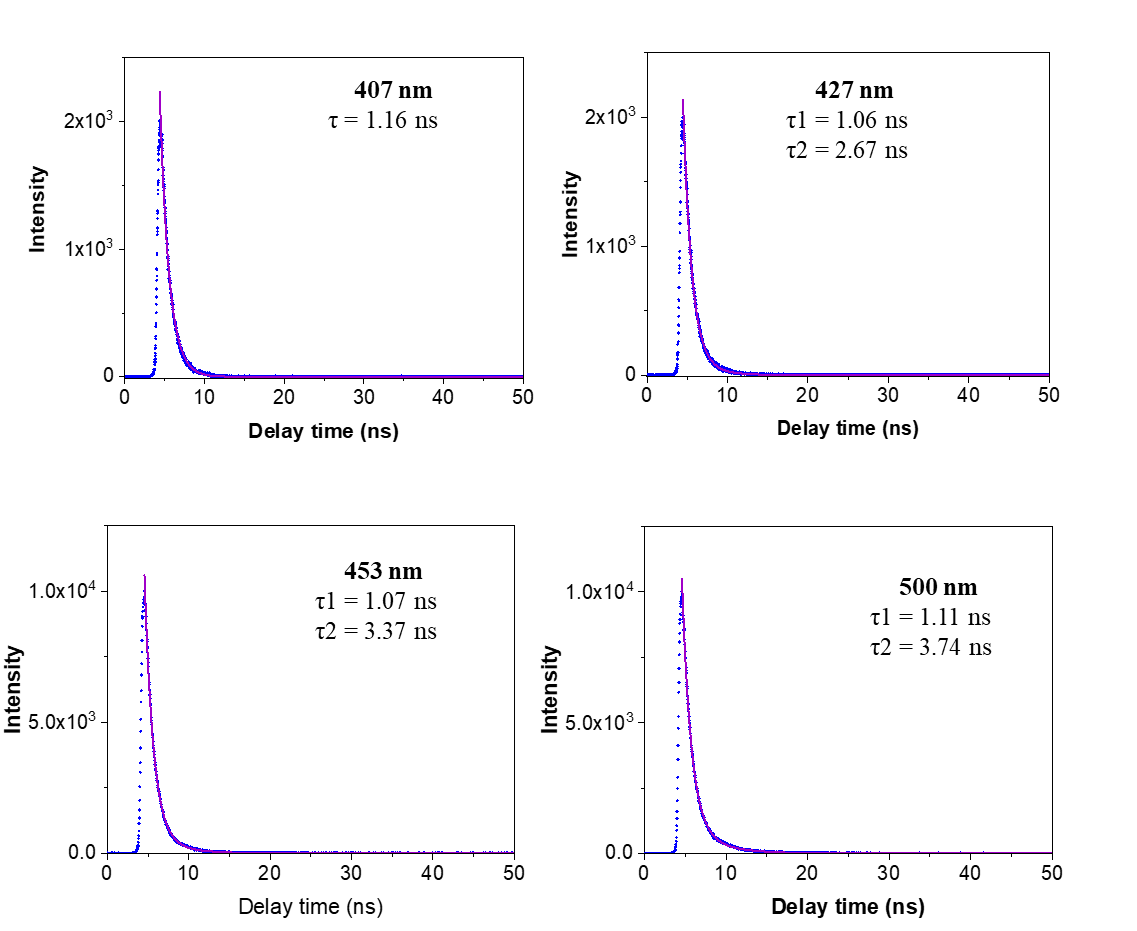


**Figure S1.** Femtosecond fluorescence transients (dots) and fitting lines of **1a** measured in aerated toluene and dichloromethane (concentration: 1×10^-5^ M) at room temperature measured at various wavelengths. Excitation wavelength: 375 nm.


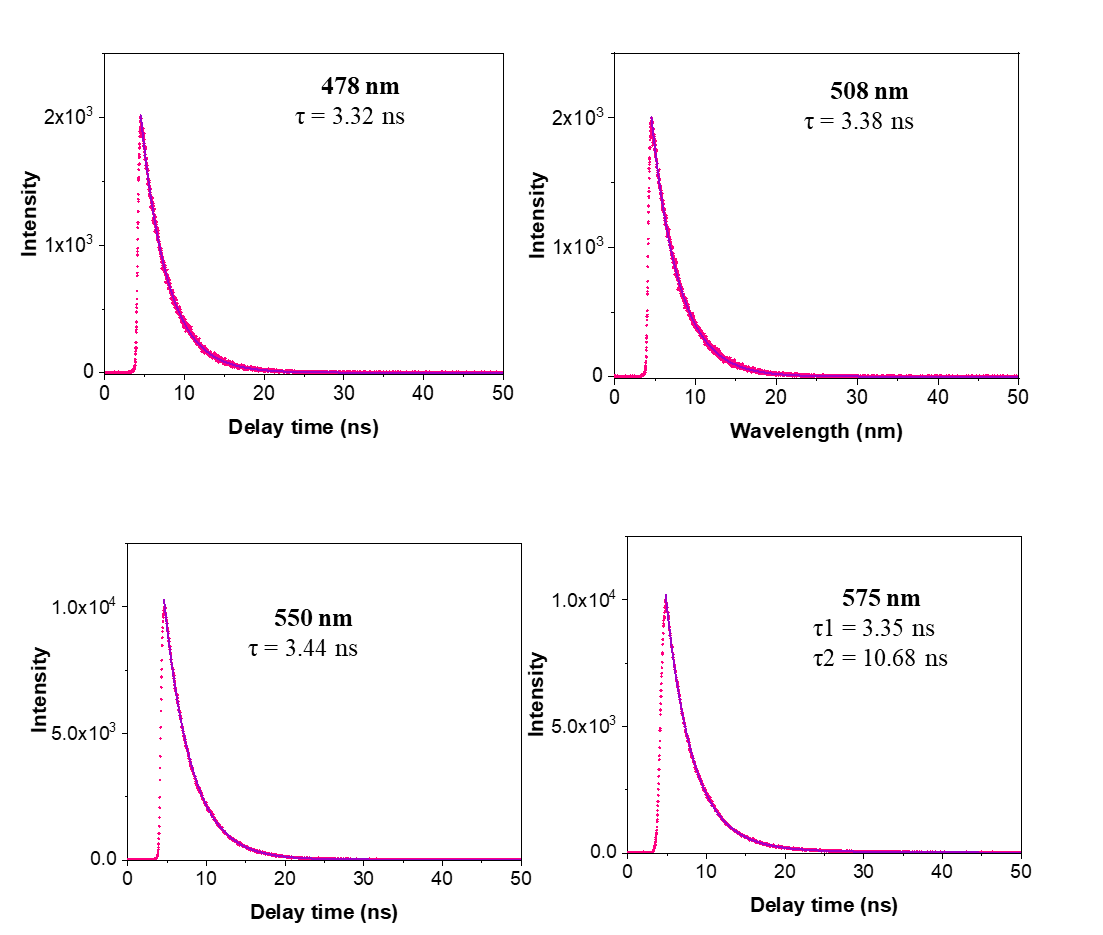


**Figure S2.** Femtosecond fluorescence transients (dots) and fitting lines of **2** measured in aerated toluene and dichloromethane (concentration: 1×10^-5^ M) at room temperature measured at various wavelengths. Excitation wavelength: 375 nm.


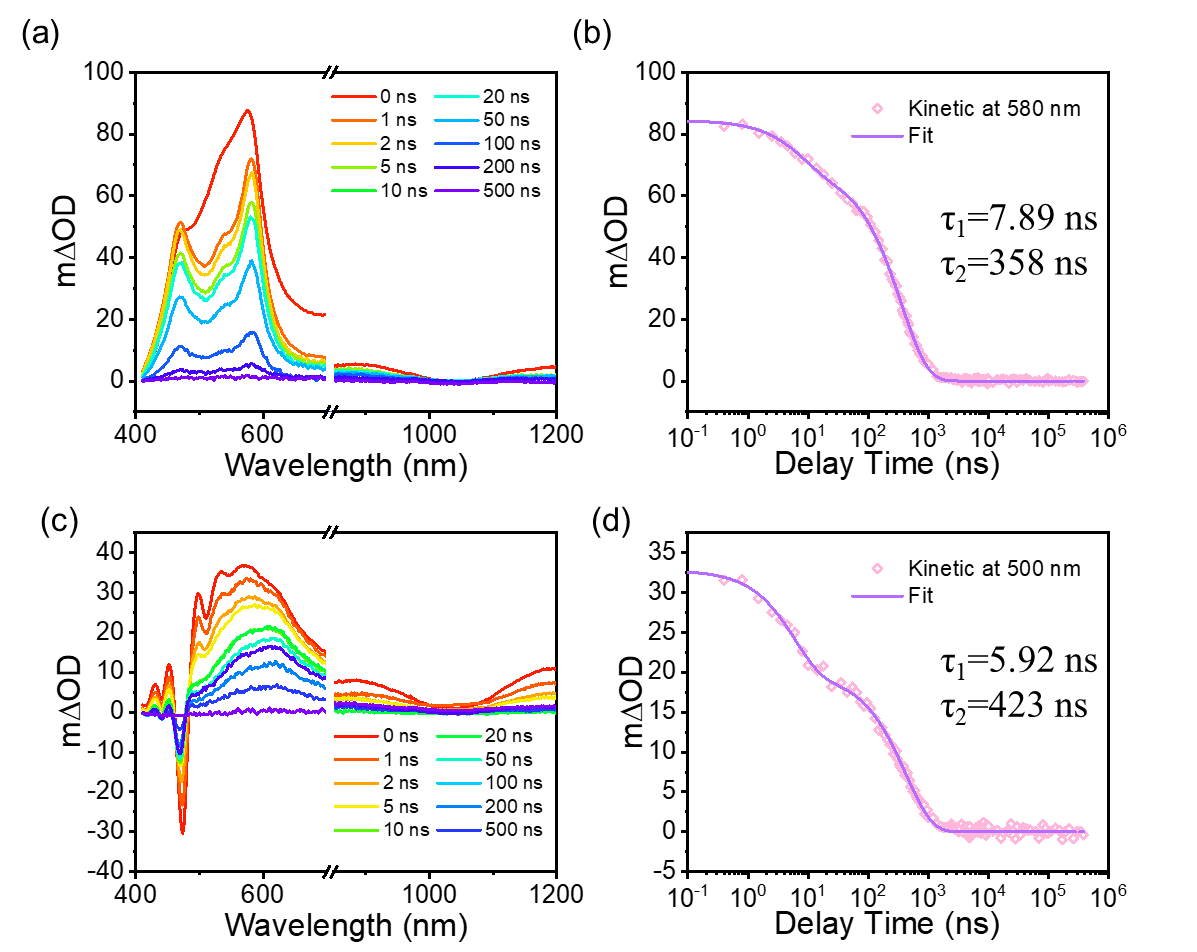


**Figure S3.** (a) Ns-TA spectra of **1a** measured in toluene solution, and (b) Single-wavelength kinetic fitting result at 500 nm. (c) Ns-TA spectra of **2** measured in toluene solution, and (d) Single-wavelength kinetic fitting result at 580 nm.


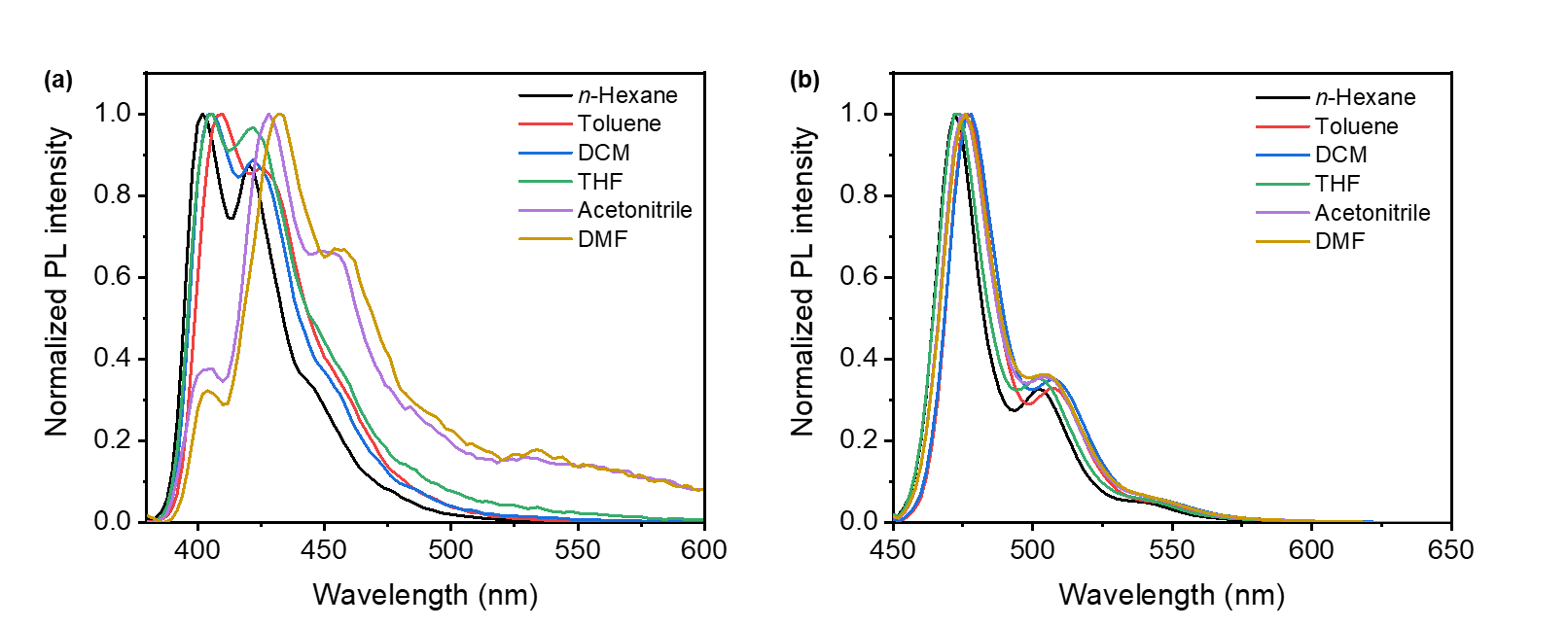


**Figure S4.** Fluorescence spectra of (a) **1a** and (b) **2** measured in different solvents with varying polarity. The concentration was 10 μM.

**Figure S5.** Variation of the UV-*vis*-NIR absorption spectra of **1a** upon chemical titration with NO•SbF_6_ in dry DCM. The concentration is 10 μM.


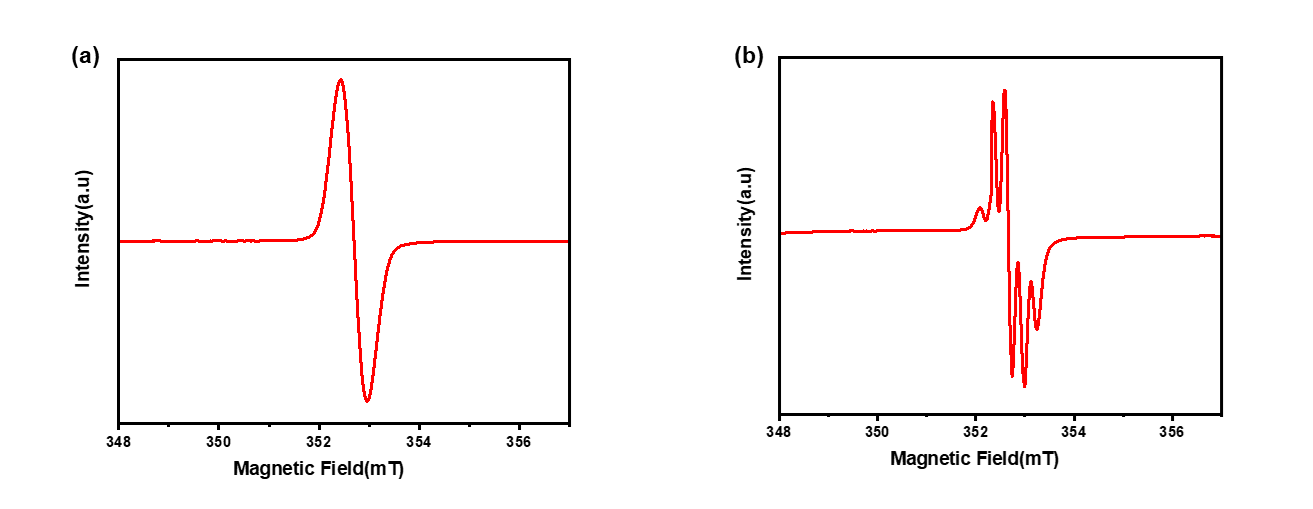


**Figure S6.** EPR spectra of the radical cations of (a) **1a** and (b) **2** in a solution of dichlorometahne at 298 K.

1. **Variable Temperature ^1^H NMR Spectra**

**
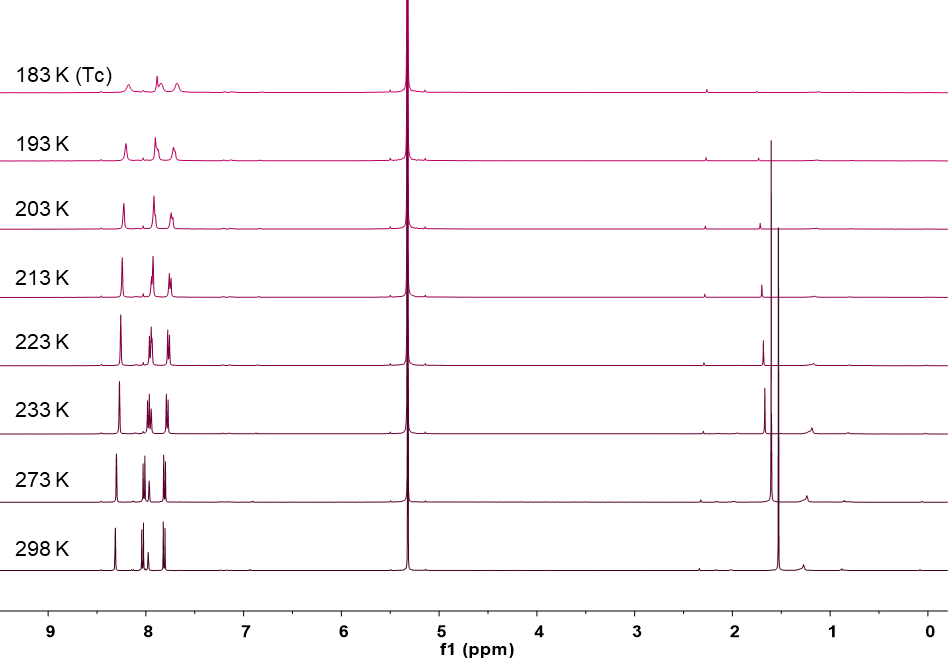
**

**Figure S7.** VT ^1^H NMR spectra (500 MHz) of **1a** measured in CD_2_Cl_2_.


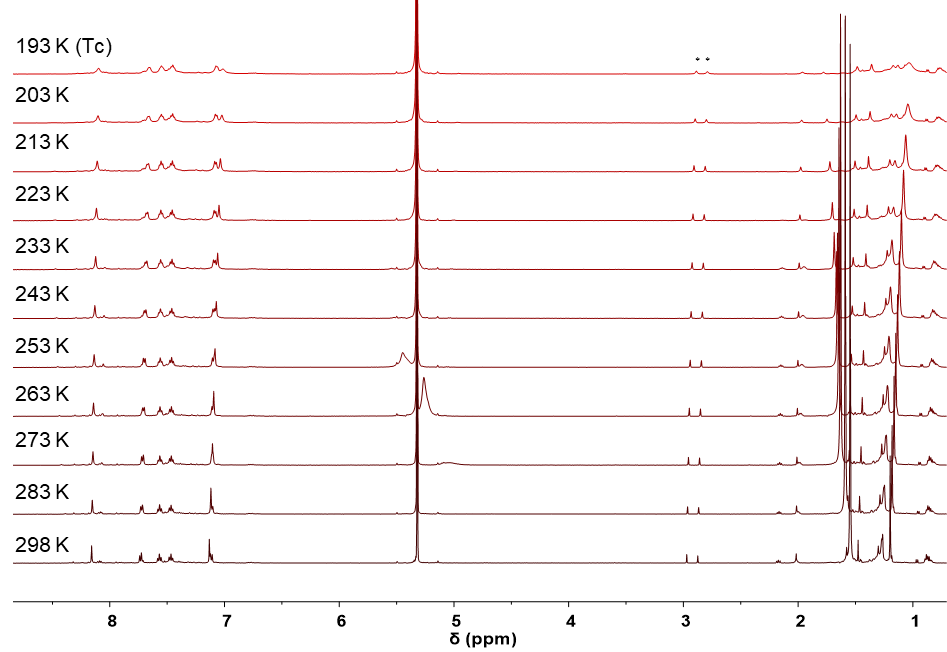


**Figure S8.** VT ^1^H NMR spectra (500 MHz) of **2** measured in CD_2_Cl_2_.

The values of the energy barrier (∆*E*) and interconversion rate (*k*) were determined based on previously reported method.^S4-S5^ As the temperature cooled down from 298 K to 193 K, the proton signals of **2** became broadened (Figure S8), indicating slowing down of the conformational interconversion. Accordingly, the value of the chemical shift difference (Δν), defined as the linewidth (Hz) at half height of peak at 8.17 ppm, was measured to be 15 Hz at the coalescence temperature (*T*_c_).

According to the equation:

*k* = $\frac{\pi\Delta Vo}{\surd2}$ (1),

the interconversion rate *k* was estimated to be 33 s^-1^ at 193 K.

And the corresponding energy barrier was calculated according the equation:

∆*E* = ∆G = R*T*_c_[23 + ln(*T*_c_/∆ν)] (2),

which gave a value of 9.8 kcal mol^-1^ at 193 K.

1. **Chiral HPLC Chromatogram**

**
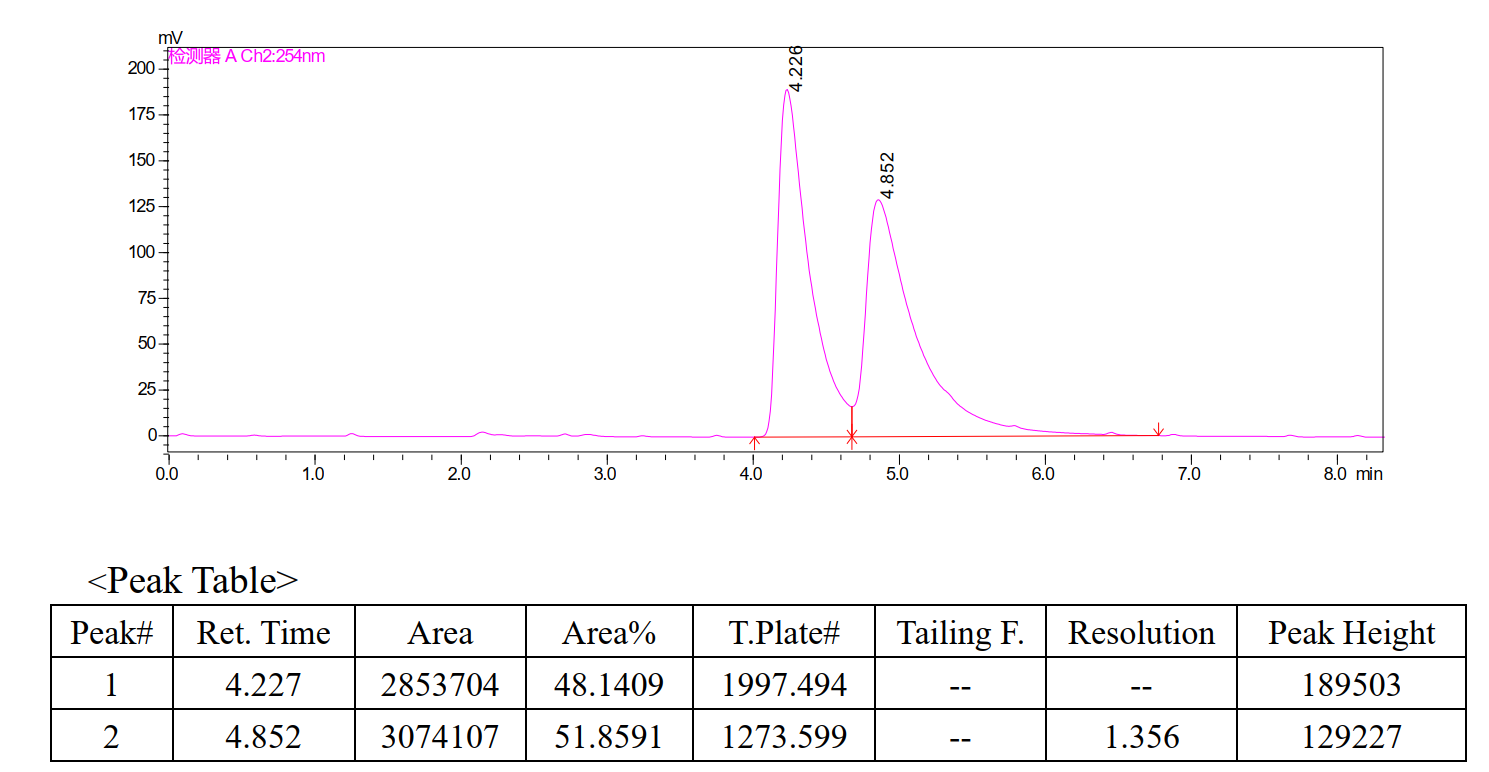
**

**Figure S9.** Resolution of **4a** by chiral HPLC monitored at 254 nm was performed with a CHIRALPAK ID (0.46 cm I.D. × 15 cm L). Injection volume was 5 µL, and a mixture of *n*-hexane/DCM = 77/23 (V/V) was used as the eluent with a flow rate of 1.0 mL/min at 25 ºC.

**
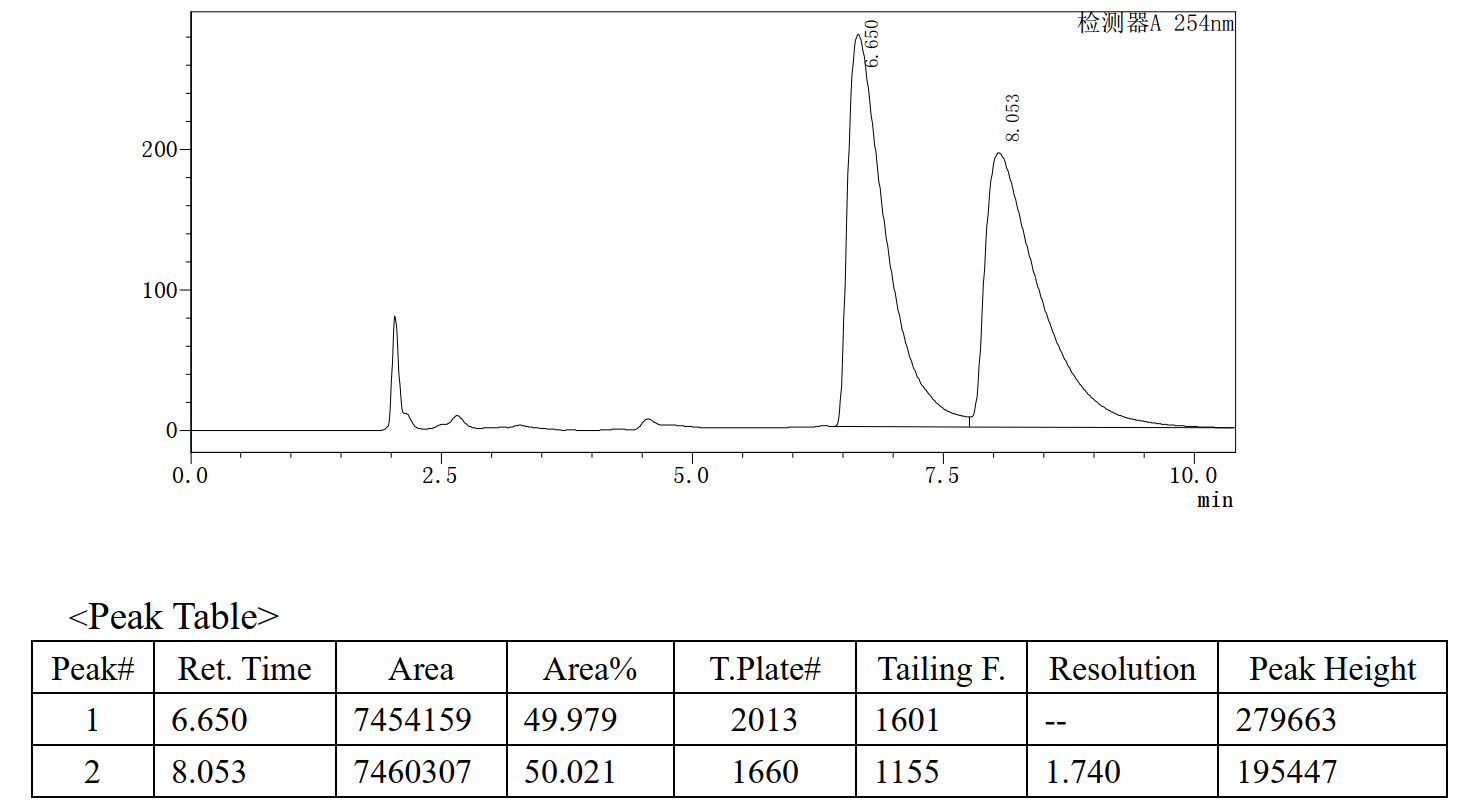
**

**Figure S10.** Resolution of **4b** by chiral HPLC monitored at 254 nm was performed with a CHIRALPAK ID (0.46 cm I.D. × 15 cm L). Injection volume was 1 µL, and a mixture of *n*-hexane/DCM = 85/15 (V/V) was used as the eluent with a flow rate of 1.0 mL/min at 25 ºC.


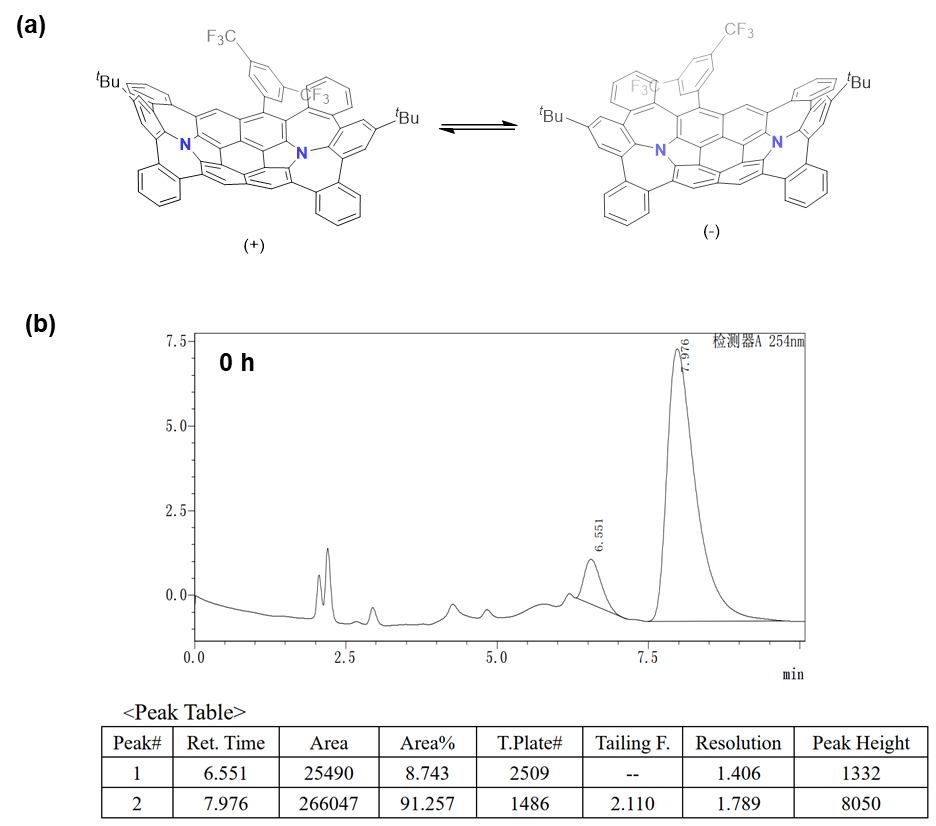


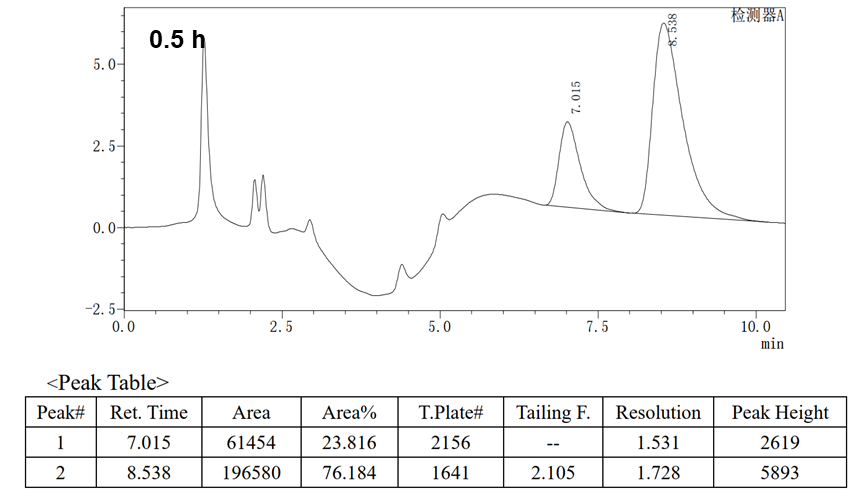


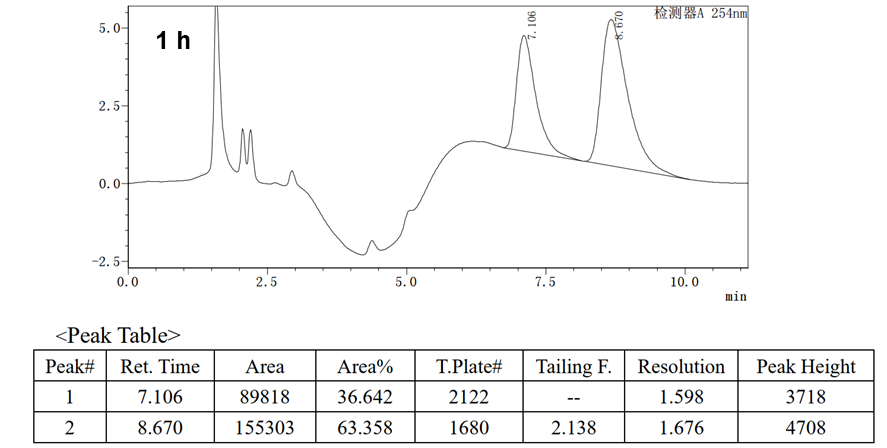


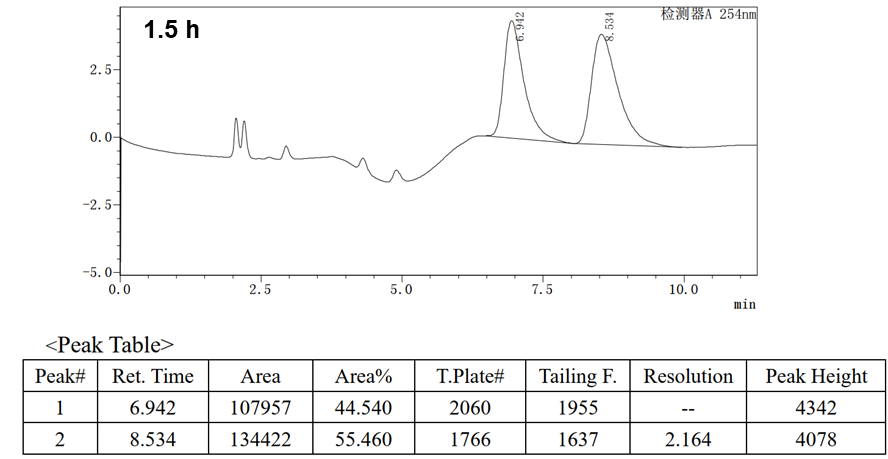


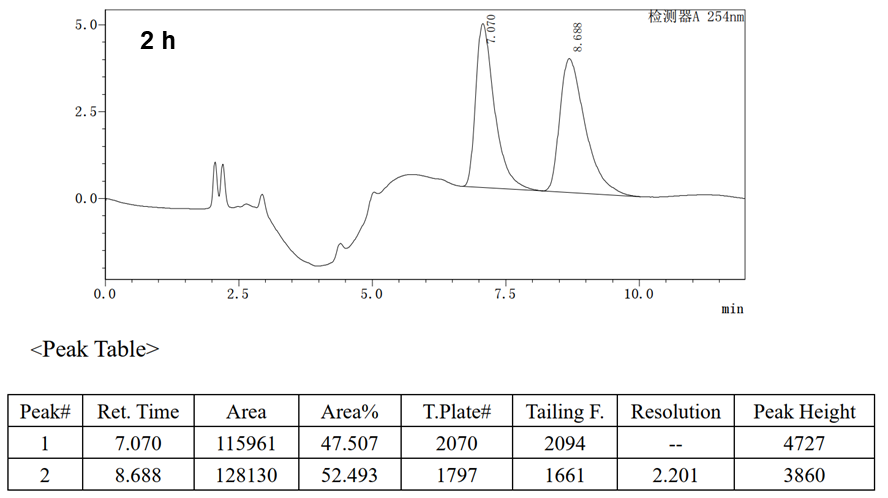


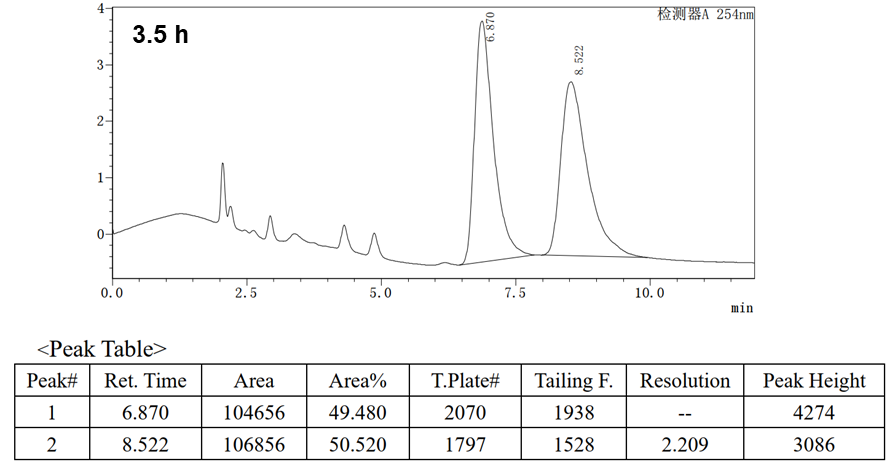


**Figure S11.** (a) Racemization of **4b** at room temperature. (b) Chiral HPLC trace of enantiopure **4b** (the second fraction at *ca.* 7.5 – 9.0 min) monitored at 254 nm with an analytic CHIRALPAK ID (0.46 cm I.D. × 15 cm L) column. Injection volume was 1 µL, and a mixture of *n*-hexane/DCM = 85/15 (V/V) was used as the eluent with a flow rate of 1.0 mL/min at 25 ºC.

The racemization between both enantiomers of **4b** follows a reversible first order reaction, with a rate constant (*k*) of 1.82×10^-4^ s^-1^, which could be determined by fitting the experimental data using the following equation: ln (2*α* - 1) = -2*k*t. The half-life time (τ_1/2_) was estimated to be 63 min according to τ_1/2_ = ln2/*k.* The energy barrier (Δ*G*) was determined by the equation ∆*G*(T) = -RTln (*k*h/κk_B_T). R is the gas constant (R = 8.314 J K^-1^); h is the Planck constant (h = 6.626 × 10^-34^ J s); k_B_ is the Boltzmann constant (k_B_ = 1.38 × 10^-23^ J K^-1^); κ is the transmission coefficient (κ = 0.5), as there is an equal probability of transformation of the transition state to either of the enantiomers.^S6^ As a result, the racemization energy barrier was determined as Δ*G* = 22.4 kcal mol^-1^.

1. **Host-guest Interactions with Fullerenes**


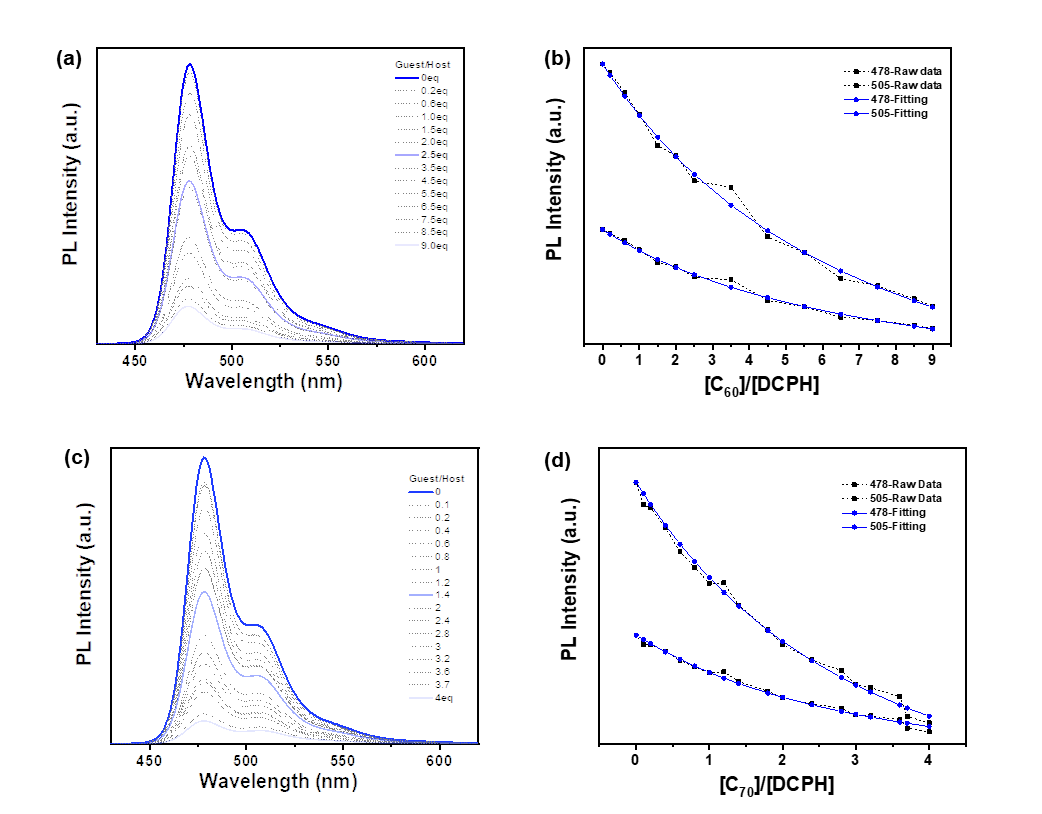


**Figure S12.** Fluorescence titration and fittings of **2** with different ratios of (a, b) C_60_ and (c, d) C_70_ measured in toluene at room temperature. The dashed lines in (b) and (d) serve as guides for the eye. The concentration of **2** was 10 μM.

1. **X-ray Crystal Structures**

Crystallographic data have been deposited with the Cambridge Crystallographic Data Centre as supplementary publication on No. CCDC 2429695 (**Per-4Cl**), 2429696 (**1a**), 2429697 (**2**), and 2429698 (**2@C60)**). These data are provided free of charge by the joint Cambridge Crystallographic Data Centre via https://www.ccdc.cam.ac.uk/structures/. The crystallographic data were summarized below.


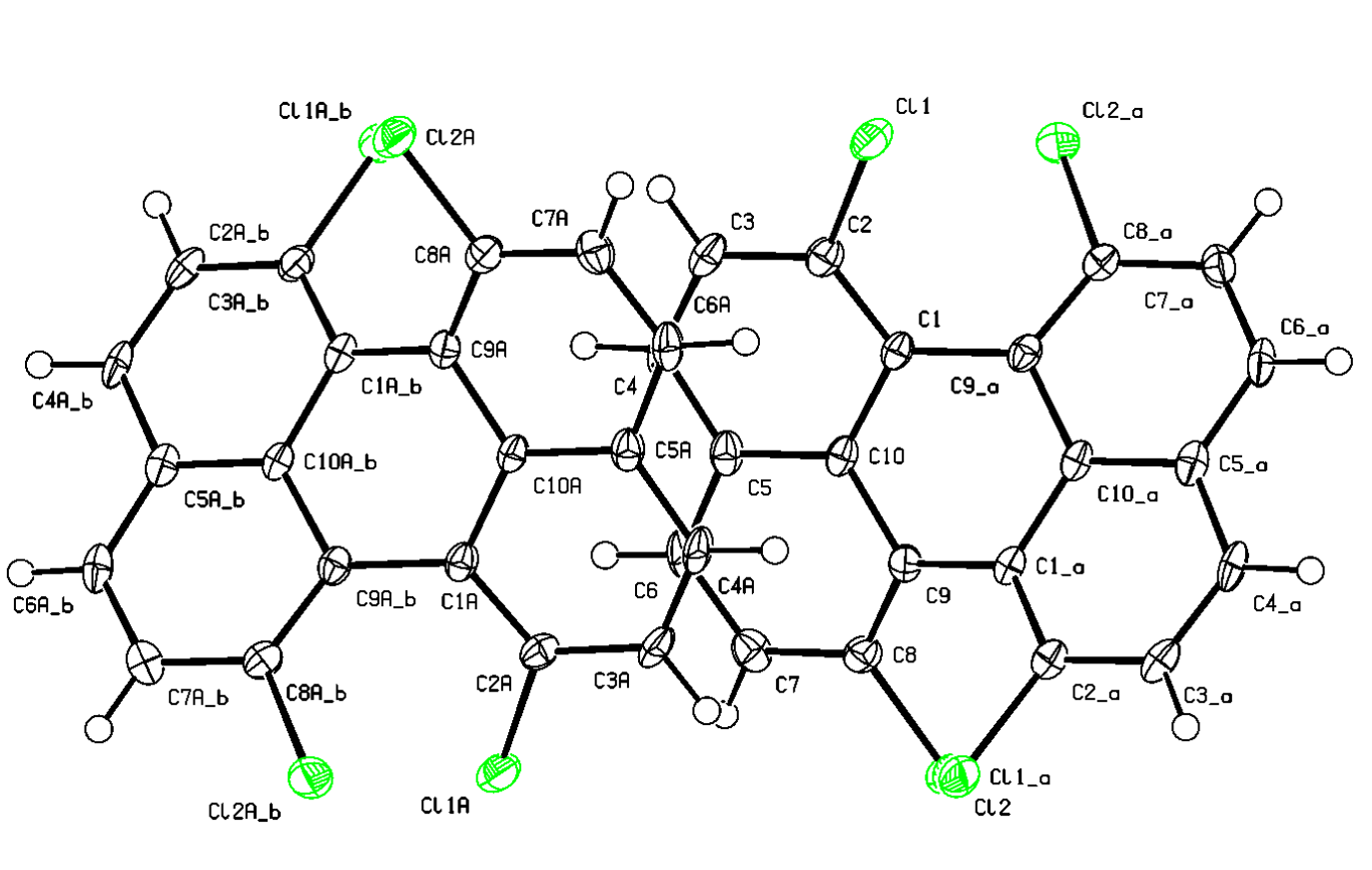


**Figure S13.** Crystal structure of **Per-4Cl** in the unit cell. Ellipsoids are drawn at 50% probability level.

**Table S1.** Crystal data and structure refinement for **Per-4Cl**.

| Empirical formula | C_20_H_8_Cl_4_ |
| --- | --- |
| Formula weight | 390.06 |
| Temperature/K | 170.00 |
| Crystal system | monoclinic |
| Space group | P2/n |
| a/Å | 11.2009(2) |
| b/Å | 7.08310(10) |
| c/Å | 19.8629(4) |
| α/° | 90 |
| β/° | 105.5150(10) |
| γ/° | 90 |
| Volume/Å^3^ | 1518.44(5) |
| Z | 4 |
| ρ_calc_g/cm^3^ | 1.706 |
| μ/mm^‑1^ | 4.688 |
| F(000) | 784.0 |
| Crystal size/mm^3^ | 0.17 × 0.17 × 0.05 |
| Radiation | GaKα (λ = 1.34139) |
| 2Θ range for data collection/° | 7.184 to 109.764 |
| Index ranges | -13 ≤ h ≤ 8, -7 ≤ k ≤ 8, -21 ≤ l ≤ 24 |
| Reflections collected | 17963 |
| Independent reflections | 2887 [R_int_ = 0.0847, R_sigma_ = 0.0565] |
| Data/restraints/parameters | 2887/0/217 |
| Goodness-of-fit on F^2^ | 1.069 |
| Final R indexes [I>=2σ (I)] | R_1_ = 0.0361, wR_2_ = 0.0927 |
| Final R indexes [all data] | R_1_ = 0.0651, wR_2_ = 0.1017 |
| Largest diff. peak/hole / e Å^-3^ | 0.42/-0.42 |


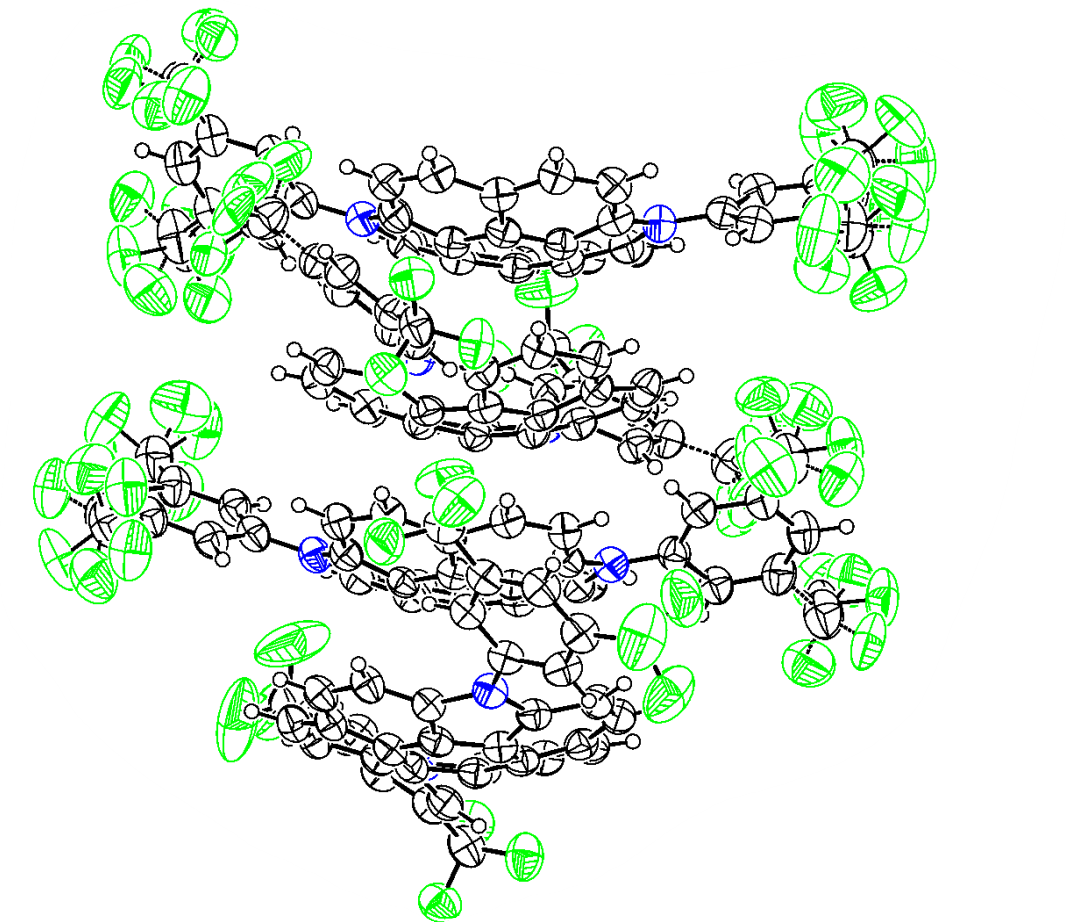


**Figure S14.** Crystal structure of **1a** in the unit cell. Ellipsoids are drawn at 50% probability level.

**Table S2.** Crystal data and structure refinement for **1a**.

| Empirical formula | C_36_H_14_F_12_N_2_ |
| --- | --- |
| Formula weight | 702.49 |
| Temperature/K | 223.15 |
| Crystal system | monoclinic |
| Space group | P2_1_/n |
| a/Å | 14.7794(9) |
| b/Å | 32.726(2) |
| c/Å | 23.5059(16) |
| α/° | 90 |
| β/° | 92.055(5) |
| γ/° | 90 |
| Volume/Å^3^ | 11361.8(12) |
| Z | 16 |
| ρ_calc_g/cm^3^ | 1.643 |
| μ/mm^‑1^ | 1.347 |
| F(000) | 5632.0 |
| Crystal size/mm^3^ | 0.14 × 0.13 × 0.11 |
| Radiation | CuKα (λ = 1.54178) |
| 2Θ range for data collection/° | 4.63 to 137.524 |
| Index ranges | -17 ≤ h ≤ 17, 0 ≤ k ≤ 39, 0 ≤ l ≤ 28 |
| Reflections collected | 20827 |
| Independent reflections | 20827 [R_int_ = ?, R_sigma_ = 0.1234] |
| Data/restraints/parameters | 20827/1302/2166 |
| Goodness-of-fit on F^2^ | 1.091 |
| Final R indexes [I>=2σ (I)] | R_1_ = 0.0885, wR_2_ = 0.2340 |
| Final R indexes [all data] | R_1_ = 0.1476, wR_2_ = 0.2891 |
| Largest diff. peak/hole / e Å^-3^ | 0.40/-0.32 |

**
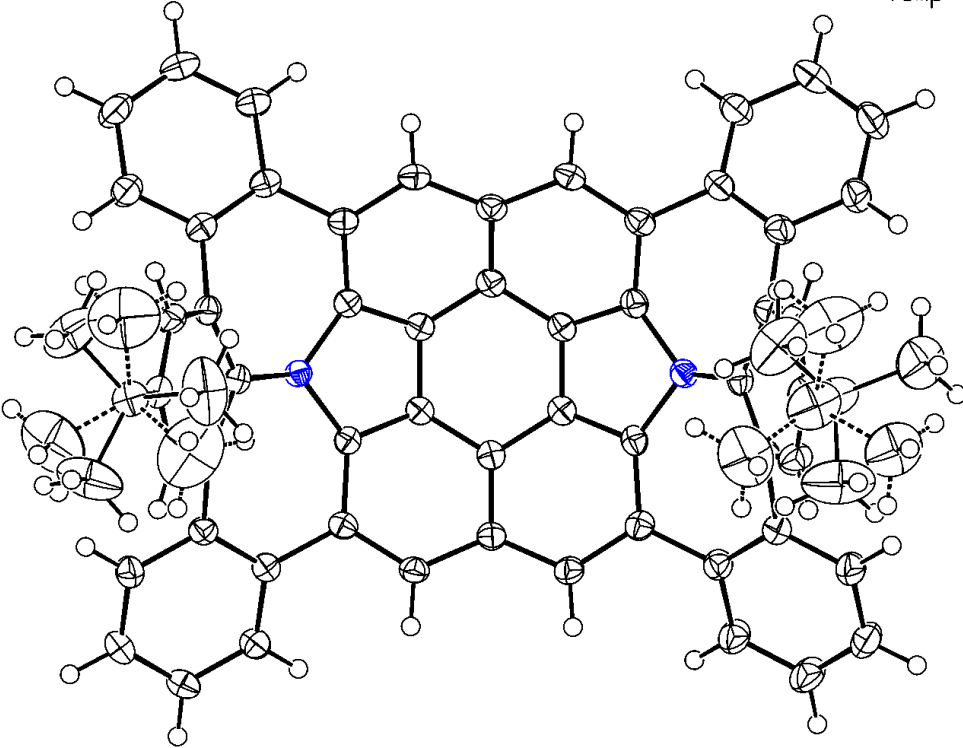
**

**Figure S15.** Crystal structure of **2** in the unit cell. Ellipsoids are drawn at 50% probability level.

**Table S3.** Crystal data and structure refinement for **2.**

| Empirical formula | C_64_H_42_N_2_ |
| --- | --- |
| Formula weight | 838.99 |
| Temperature/K | 173.00 |
| Crystal system | monoclinic |
| Space group | P2_1_/n |
| a/Å | 18.4259(3) |
| b/Å | 12.9254(2) |
| c/Å | 21.0586(3) |
| α/° | 90 |
| β/° | 101.2530(10) |
| γ/° | 90 |
| Volume/Å^3^ | 4918.94(13) |
| Z | 4 |
| ρ_calc_g/cm^3^ | 1.133 |
| μ/mm^‑1^ | 0.498 |
| F(000) | 1760.0 |
| Crystal size/mm^3^ | 0.13 × 0.12 × 0.11 |
| Radiation | CuKα (λ = 1.54178) |
| 2Θ range for data collection/° | 8.41 to 136.582 |
| Index ranges | -19 ≤ h ≤ 22, -15 ≤ k ≤ 14, -25 ≤ l ≤ 25 |
| Reflections collected | 44402 |
| Independent reflections | 8990 [R_int_ = 0.0351, R_sigma_ = 0.0269] |
| Data/restraints/parameters | 8990/163/671 |
| Goodness-of-fit on F^2^ | 1.039 |
| Final R indexes [I>=2σ (I)] | R_1_ = 0.0419, wR_2_ = 0.1063 |
| Final R indexes [all data] | R_1_ = 0.0477, wR_2_ = 0.1099 |
| Largest diff. peak/hole / e Å^-3^ | 0.29/-0.23 |

**Table S4.** Crystal data and structure refinement for **2@C60**.

| Empirical formula | C_262_H_57_Cl_3_N_2_ |
| --- | --- |
| Formula weight | 3338.44 |
| Temperature/K | 193.00 |
| Crystal system | monoclinic |
| Space group | C2/c |
| a/Å | 36.998(2) |
| b/Å | 20.8982(11) |
| c/Å | 19.7775(10) |
| α/° | 90 |
| β/° | 106.048(4) |
| γ/° | 90 |
| Volume/Å^3^ | 14696.0(14) |
| Z | 4 |
| ρ_calc_g/cm^3^ | 1.509 |
| μ/mm^‑1^ | 1.161 |
| F(000) | 6776.0 |
| Crystal size/mm^3^ | 0.14 × 0.13 × 0.11 |
| Radiation | CuKα (λ = 1.54178) |
| 2Θ range for data collection/° | 4.648 to 136.48 |
| Index ranges | -44 ≤ h ≤ 44, -25 ≤ k ≤ 25, -23 ≤ l ≤ 23 |
| Reflections collected | 65253 |
| Independent reflections | 13310 [R_int_ = 0.0995, R_sigma_ = 0.0557] |
| Data/restraints/parameters | 13310/25878/2112 |
| Goodness-of-fit on F^2^ | 1.186 |
| Final R indexes [I>=2σ (I)] | R_1_ = 0.0993, wR_2_ = 0.2704 |
| Final R indexes [all data] | R_1_ = 0.1389, wR_2_ = 0.3170 |
| Largest diff. peak/hole / e Å^-3^ | 0.56/-0.66 |


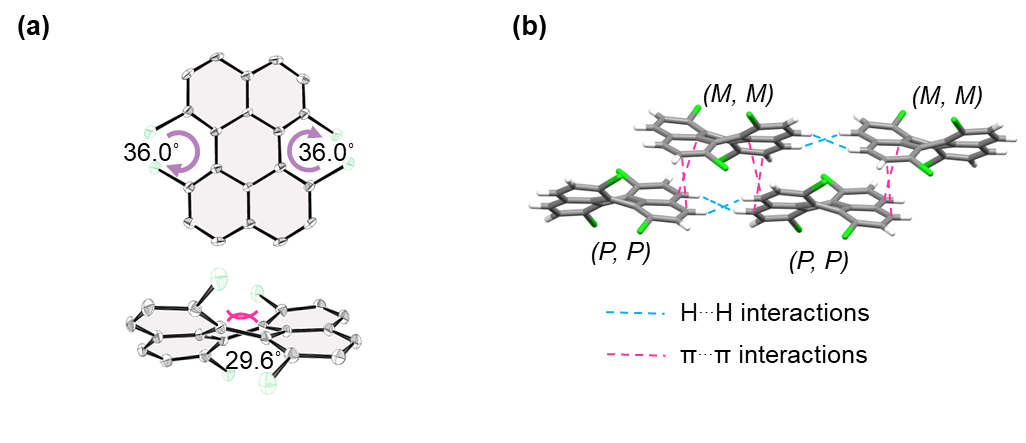


**Figure S16.** (a) molecular structure and (b) intermolecular interactions of **Per-4Cl**


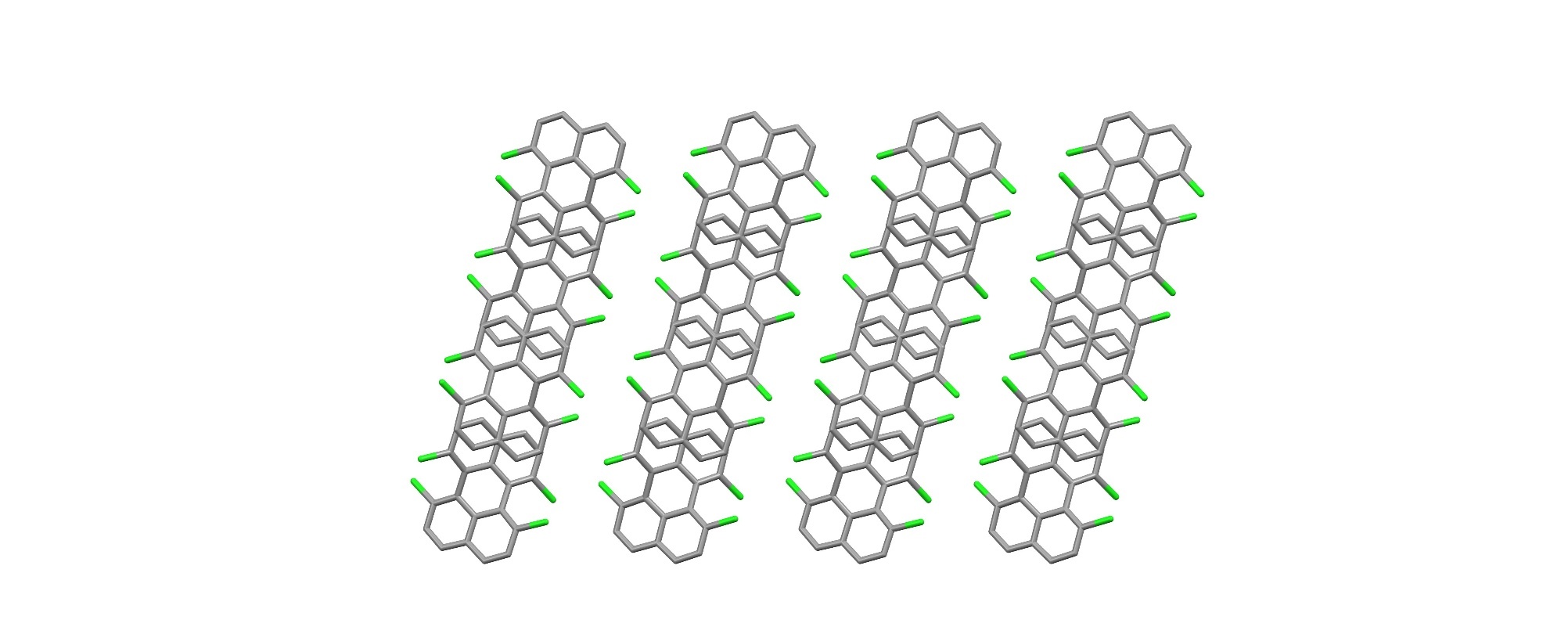


**Figure S17.** Molecular arrangements of **Per-4Cl** along *b* axis. Hydrogen atoms are omitted for clarity.


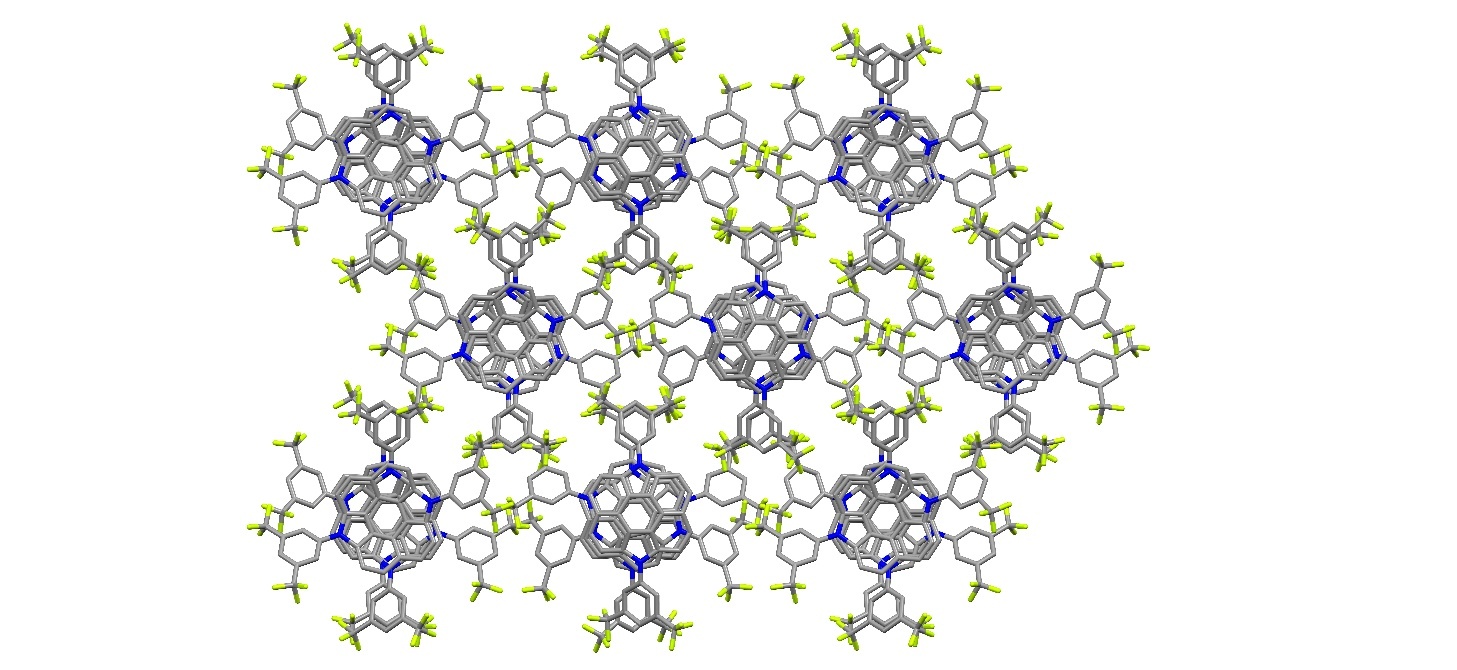


**Figure S18.** Molecular arrangements of **1a** along *b* axis. Hydrogen atoms are omitted for clarity.

**Figure S19.** Molecular arrangements of **2** along *b* axis.


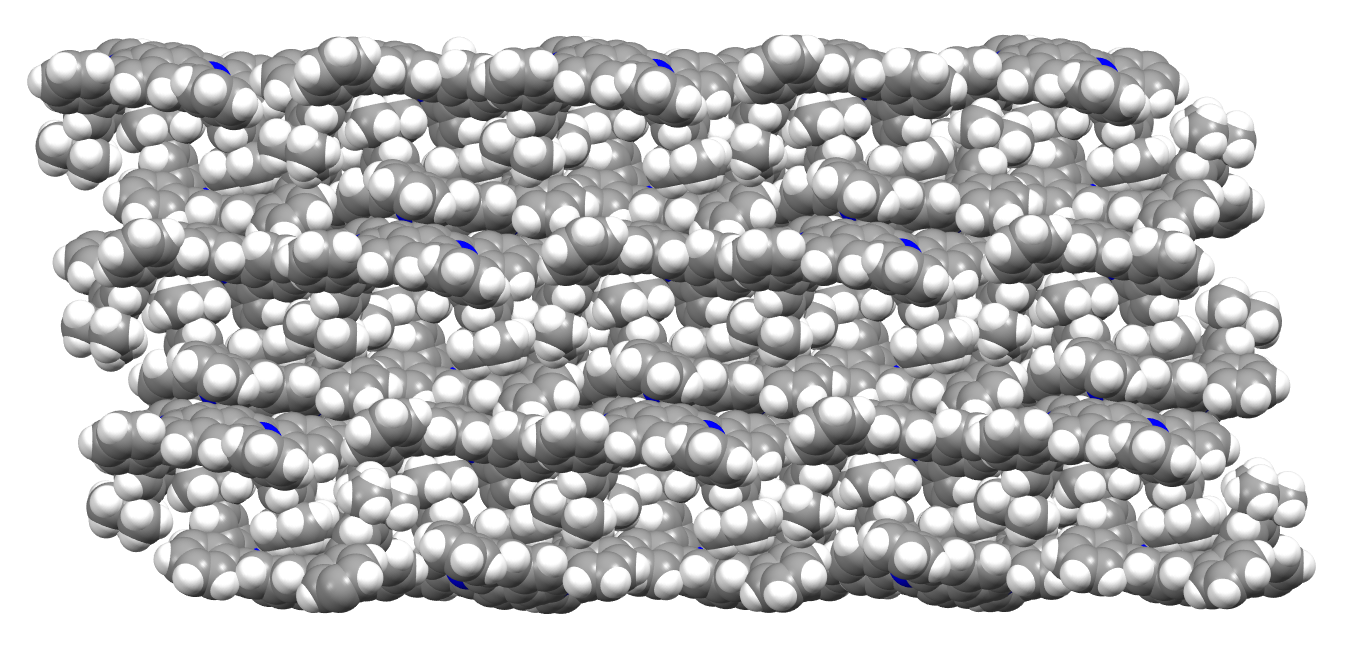

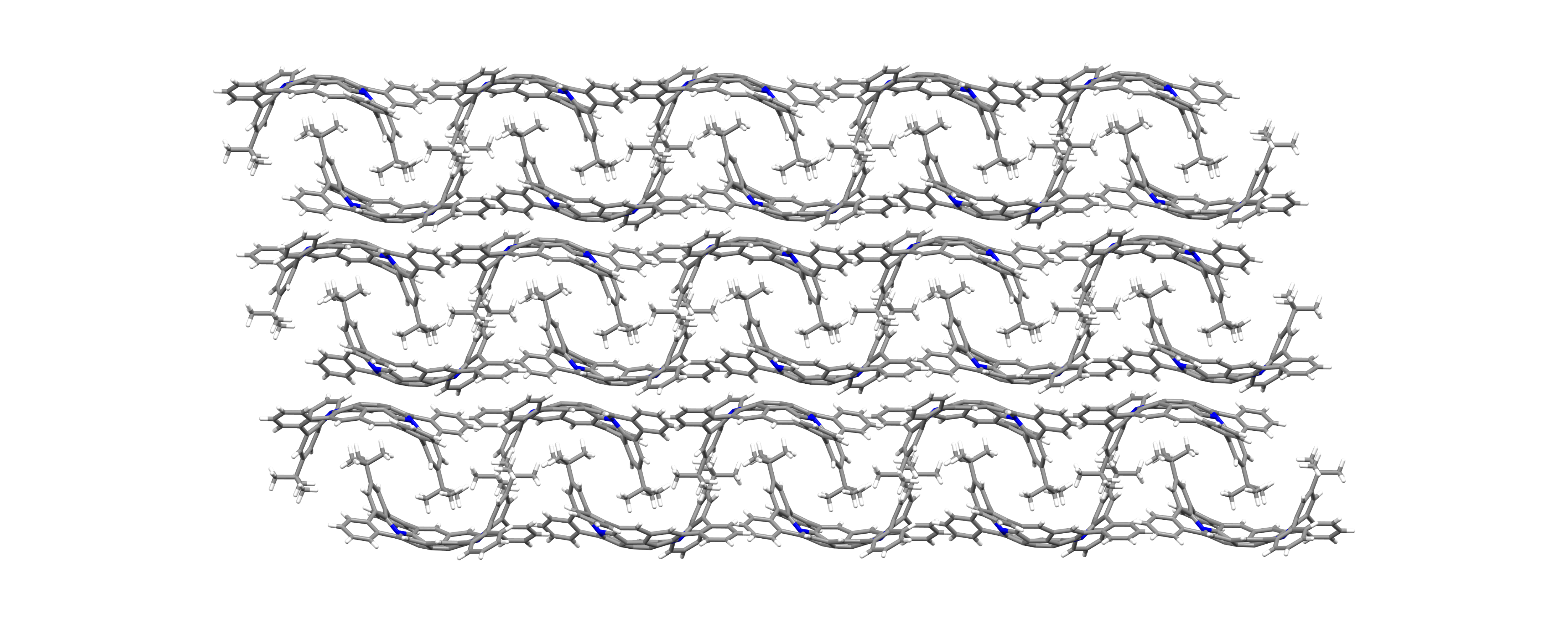


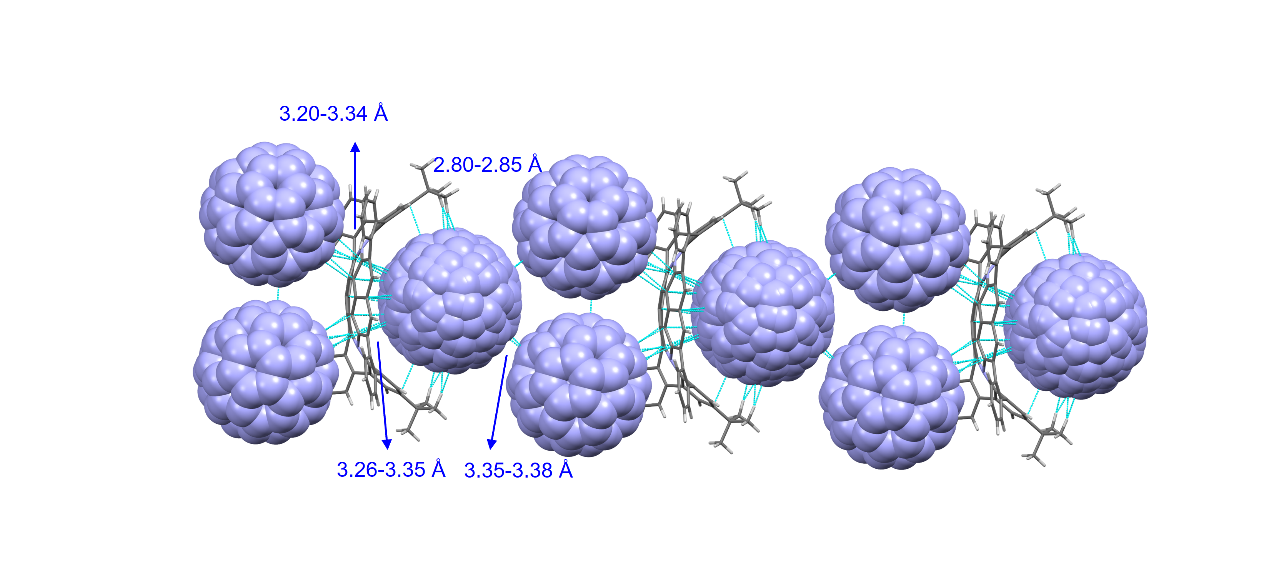


**Figure S20.** Molecular arrangements of **2** and C_60_ molecules in the crystal. Cyan lines represent intermolecular non-covalent interactions.

1. **Theoretical Calculations**

For all titled molecules, DFT and time-dependent DFT (TD-DFT) calculations were performed using the Gaussian 16 software package.^S7^ The geometries were optimized with the B3LYP functional and 6-31G(d) basis set. The excited state structures were calculated by the TD-DFT method with B3LYP/6-31(d) hybrid function. NICS(1)zz values were calculated at the B3LYP/6-311(d,p) level of theory, and analyzed with Multiwfn 3.8.^S8^ Three-dimensional isochemical shielding surface and independent gradient model based on Hirshfeld partition (IGMH) analyses^S9^ were analyzed with Multiwfn 3.8, and rendered with VMD 1.9.3.^S10^ AICD plot was calculated by using the method developed by Herges.^S11^


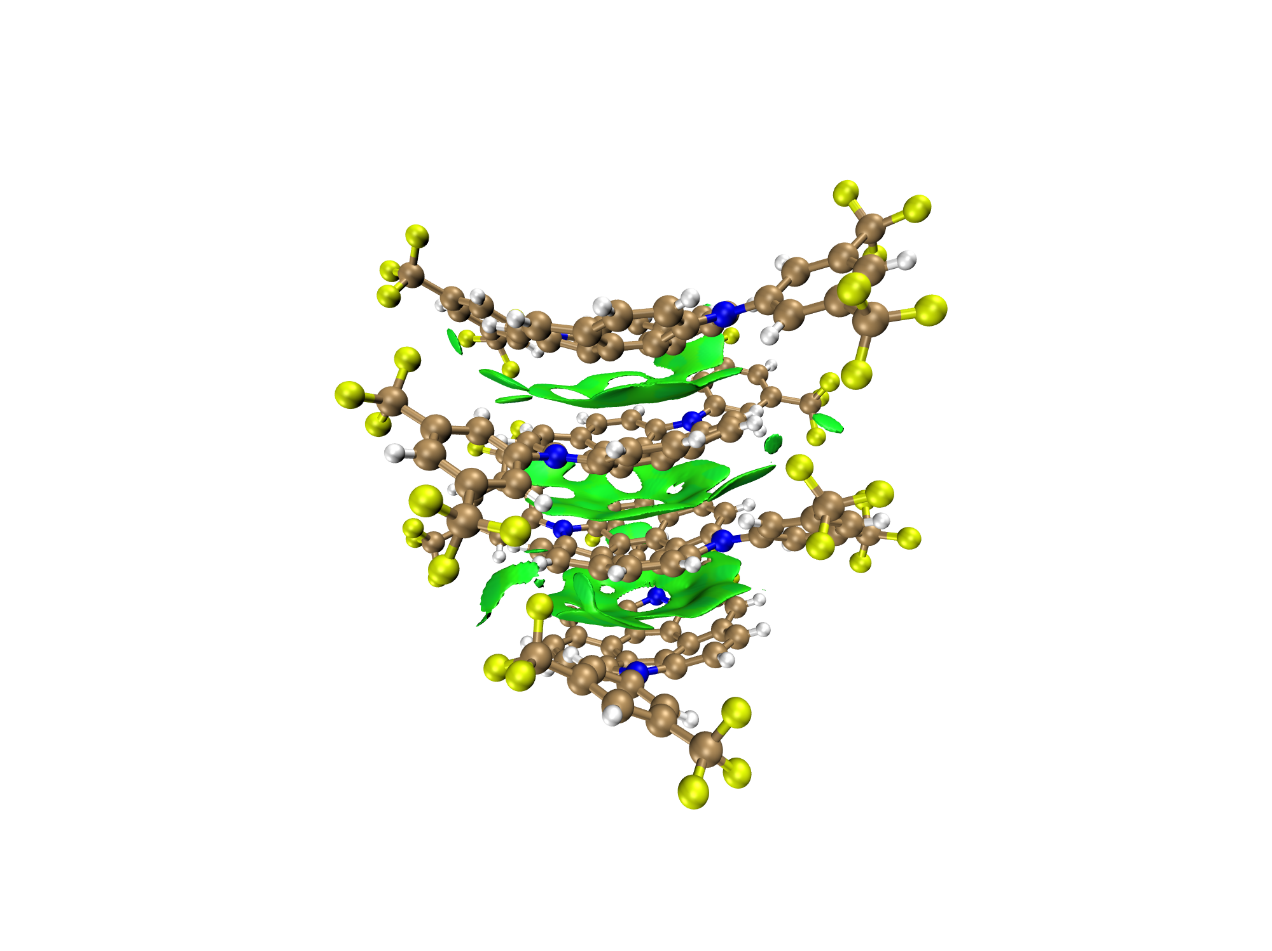


**Figure S21.** Calculated IGMH analyses of **1a** based on the crystal structure. The isovalue is 0.004.

**Table S5.** Calculated HOMA values based on crystal structures and NICS(1)zz values based on optimized structures of **Per-4Cl**, **1a**, **1a^2+^**, **2** and **2^2+^**.

^
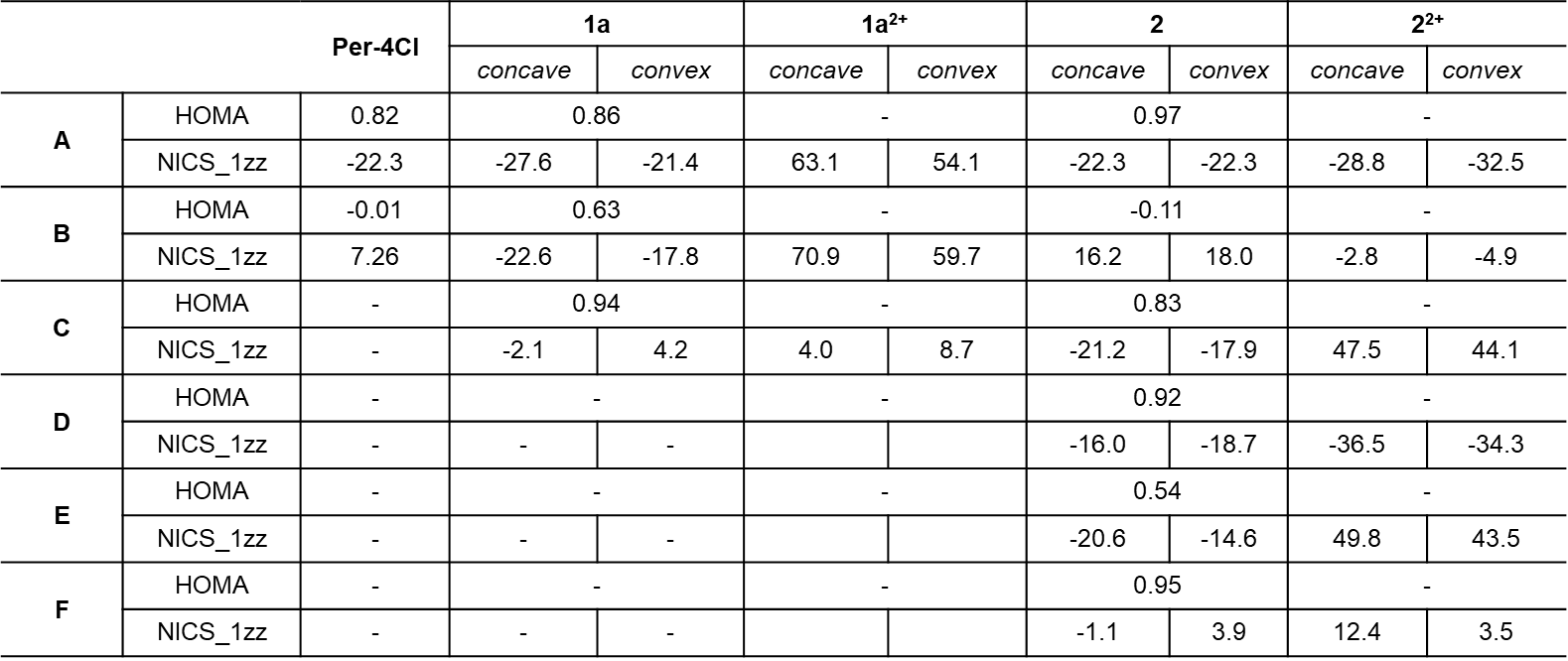
^


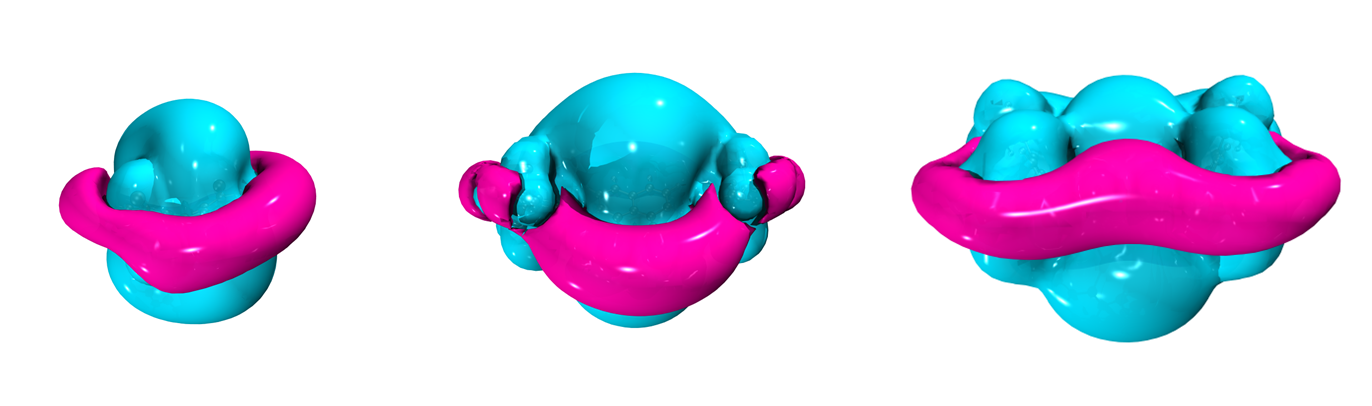


**Figure S22.** Three-dimensional ICSS maps for **Per-4Cl** (left), **1a** (middle) and **2** (right). Blue, isovalue = 0.4; red, isovalue = -0.3.


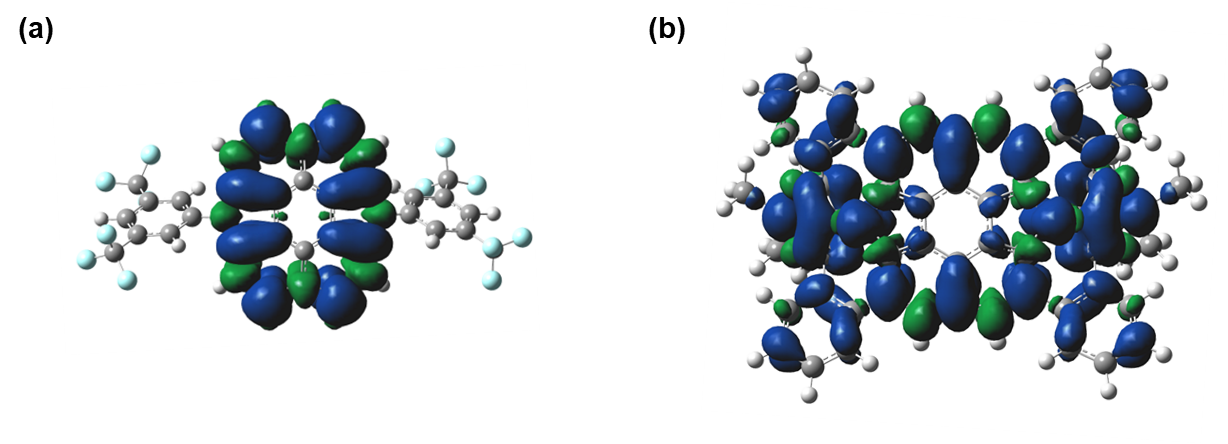


**Figure S23.** Spin density distributions of the radical cations of **1a** and **2** calculated at the uB3LYP/6-31g(d) level of theory.


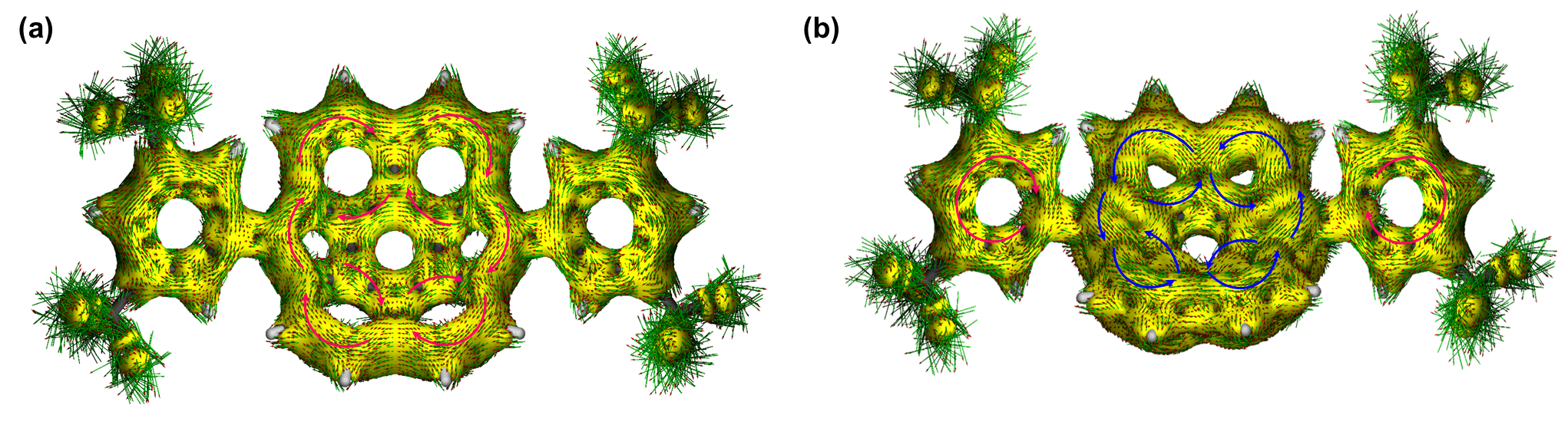


**Figure S24.** Calculated ACID plots of (a) **1a** and (b) **1a^2+^**.


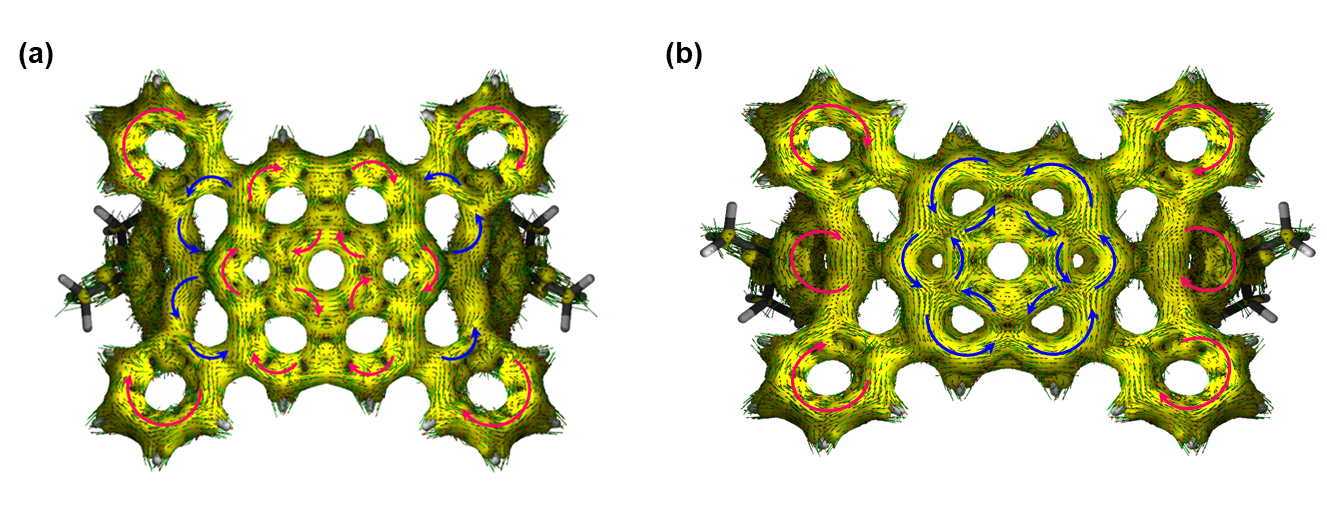


**Figure S25.** Calculated ACID plots of (a) **2** and (b) **2^2+^**.


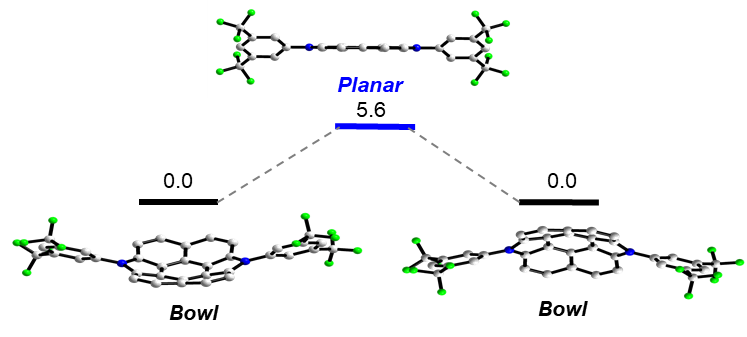


**Figure S26.** Calculated pathway for the bowl-to-bowl inversion of **1a** with relative Gibbs free energy (kcal mol^-1^) as calculated at the B3LYP/6-31G(d) level.


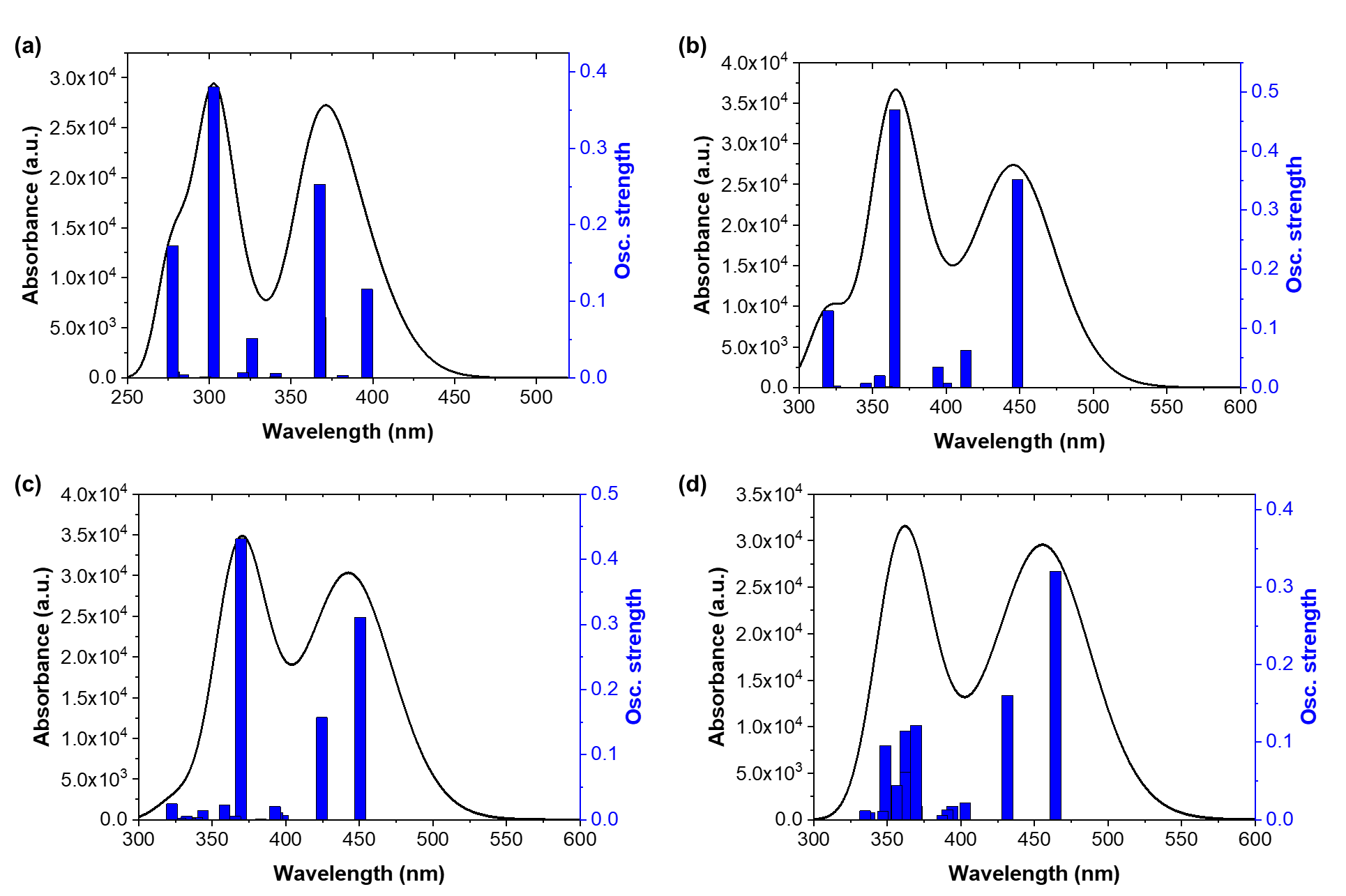


**Figure S27.** Simulated absorptions of (a) **1a**, (b) **2**, (c) **4a** and (d) **4b** calculated at the B3LYP/6-31G(d) level.

**Table S6.** Electronic transitions of **1a** calculated at the TD-B3LYP/6-31g(d) level of theory.

| **No.** | **Wavelength (nm)** | **Osc. Strength** | **Major contribs** | **Minor contribs** |
| --- | --- | --- | --- | --- |
| **1** | 396.4169 | 0.1157 | HOMO->LUMO (94%) | H-1->L+6 (2%) |
| **2** | 382.5584 | 0.0003 | HOMO->L+1 (99%) | |
| **3** | 381.4285 | 0.0032 | HOMO->L+2 (98%) | |
| **4** | 368.1981 | 0.0782 | H-1->LUMO (21%), HOMO->L+3 (74%) | HOMO->L+6 (4%) |
| **5** | 367.565 | 0.2527 | H-1->LUMO (65%), HOMO->L+3 (24%) | HOMO->L+5 (2%), HOMO->L+6 (8%) |
| **6** | 340.7353 | 0.0061 | H-1->L+1 (98%) | |
| **7** | 340.1557 | 0.0015 | H-1->L+2 (98%) | |
| **8** | 326.0317 | 0.0513 | HOMO->L+4 (94%) | H-1->L+6 (3%) |
| **9** | 320.6853 | 0.0066 | H-1->L+3 (97%) | |
| **10** | 314.9343 | 0.0007 | HOMO->L+5 (89%) | HOMO->L+6 (8%) |
| **11** | 302.7011 | 0.38 | H-1->L+4 (63%), HOMO->L+6 (29%) | H-1->LUMO (5%) |
| **12** | 297.7077 | 0.0013 | H-2->LUMO (27%), HOMO->L+7 (67%) | H-1->L+5 (2%) |
| **13** | 284.0527 | 0.0036 | H-2->LUMO (53%), H-1->L+5 (31%), HOMO->L+7 (11%) | H-1->L+6 (3%) |
| **14** | 278.3765 | 0.0074 | H-4->LUMO (14%), H-3->LUMO (48%), H-1->L+7 (28%) | HOMO->L+6 (2%), HOMO->L+8 (3%) |
| **15** | 277.6534 | 0.1727 | H-1->L+4 (30%), HOMO->L+6 (41%) | H-4->L+7 (3%), H-3->LUMO (5%), H-2->L+3 (5%), H-2->L+5 (3%), H-1->LUMO (5%), HOMO->L+5 (4%) |

**Table S7.** Electronic transitions of **2** calculated at the TD-B3LYP/6-31g(d) level of theory.

| **No.** | **Wavelength (nm)** | **Osc. Strength** | **Major contribs** | **Minor contribs** |
| --- | --- | --- | --- | --- |
| **1** | 448.273 | 0.3514 | HOMO->LUMO (89%) | H-1->L+2 (7%) |
| **2** | 413.2916 | 0.0632 | H-1->LUMO (82%), HOMO->L+2 (12%) | HOMO->L+1 (3%) |
| **3** | 399.8946 | 0.0077 | HOMO->L+1 (89%) | HOMO->L+2 (6%) |
| **4** | 394.3616 | 0.0347 | H-1->LUMO (13%), HOMO->L+2 (79%) | H-2->L+3 (3%), HOMO->L+1 (4%) |
| **5** | 389.1993 | 0.0005 | H-1->L+1 (75%), H-1->L+2 (16%) | H-3->LUMO (3%), HOMO->L+3 (4%) |
| **6** | 383.8018 | 0 | HOMO->L+3 (90%) | H-2->L+2 (3%), H-1->L+1 (4%) |
| **7** | 365.0219 | 0.4696 | H-1->L+1 (15%), H-1->L+2 (72%) | HOMO->LUMO (8%) |
| **8** | 359.904 | 0.0011 | H-2->LUMO (22%), H-1->L+3 (74%) | |
| **9** | 354.9175 | 0.0199 | H-2->LUMO (73%), H-1->L+3 (21%) | H-5->LUMO (2%) |
| **10** | 345.4441 | 0.0071 | H-2->L+1 (12%), HOMO->L+4 (66%), HOMO->L+5 (15%) | H-2->L+2 (3%) |
| **11** | 335.9709 | 0.0008 | H-3->LUMO (84%) | HOMO->L+5 (8%) |
| **12** | 335.0901 | 0.0003 | HOMO->L+4 (17%), HOMO->L+5 (65%) | H-3->LUMO (9%) |
| **13** | 328.1979 | 0.0002 | H-1->L+4 (84%) | H-4->LUMO (3%), H-1->L+5 (8%), HOMO->L+6 (2%) |
| **14** | 324.6742 | 0.0022 | H-1->L+5 (79%) | H-1->L+4 (8%), HOMO->L+7 (6%) |
| **15** | 319.7672 | 0.1296 | HOMO->L+6 (82%) | H-4->LUMO (3%), H-2->L+5 (3%), H-1->L+5 (2%) |

**Table S8.** Electronic transitions of **4a** calculated at the TD-B3LYP/6-31g(d) level of theory.

| **No.** | **Wavelength (nm)** | **Osc. Strength** | **Major contribs** | **Minor contribs** |
| --- | --- | --- | --- | --- |
| **1** | 450.6191 | 0.3111 | HOMO->LUMO (87%) | H-1->L+2 (7%) |
| **2** | 424.4551 | 0.1573 | H-1->LUMO (90%) | HOMO->LUMO (2%), HOMO->L+2 (4%) |
| **3** | 397.9181 | 0.0064 | HOMO->L+1 (87%) | H-1->L+1 (2%), HOMO->L+2 (6%) |
| **4** | 394.3741 | 0.0114 | H-1->L+1 (60%), HOMO->L+2 (23%) | H-1->L+2 (4%), HOMO->L+1 (6%) |
| **5** | 392.7003 | 0.0203 | H-1->L+1 (18%), H-1->L+2 (11%), HOMO->L+2 (60%) | H-1->LUMO (3%) |
| **6** | 382.9483 | 0.0009 | HOMO->L+3 (87%) | H-2->L+2 (3%), H-1->L+2 (3%), H-1->L+3 (3%) |
| **7** | 369.603 | 0.4316 | H-1->L+1 (13%), H-1->L+2 (70%) | HOMO->LUMO (8%), HOMO->L+2 (3%) |
| **8** | 365.7002 | 0.0053 | H-1->L+3 (87%) | H-2->LUMO (4%), HOMO->L+3 (3%) |
| **9** | 358.3955 | 0.0229 | H-2->LUMO (90%) | H-1->L+3 (4%) |
| **10** | 343.7965 | 0.0141 | H-2->L+1 (13%), HOMO->L+4 (59%), HOMO->L+5 (19%) | |
| **11** | 339.7735 | 0.0037 | H-3->LUMO (93%) | HOMO->L+3 (2%) |
| **12** | 334.8458 | 0.0023 | HOMO->L+4 (17%), HOMO->L+5 (63%) | H-1->L+4 (9%), H-1->L+7 (4%) |
| **13** | 332.5644 | 0.0053 | H-1->L+4 (72%), H-1->L+5 (11%) | HOMO->L+4 (8%), HOMO->L+5 (4%) |
| **14** | 328.6155 | 0.0021 | H-1->L+4 (11%), H-1->L+5 (75%) | H-4->LUMO (4%), HOMO->L+7 (4%) |
| **15** | 322.5289 | 0.0244 | H-4->LUMO (47%), HOMO->L+7 (33%) | H-2->L+2 (5%), HOMO->L+6 (5%) |

**Table S9.** Electronic transitions of **4b** calculated at the TD-B3LYP/6-31g(d) level of theory.

| **No.** | **Wavelength (nm)** | **Osc. Strength** | **Major contribs** | **Minor contribs** |
| --- | --- | --- | --- | --- |
| **1** | 464.218 | 0.3202 | HOMO->LUMO (92%) | H-1->L+2 (3%) |
| **2** | 431.5316 | 0.1604 | H-1->LUMO (92%) | |
| **3** | 402.8179 | 0.0218 | HOMO->L+1 (87%) | H-1->L+2 (3%), HOMO->L+2 (3%) |
| **4** | 394.0482 | 0.0173 | H-1->L+1 (14%), HOMO->L+2 (74%) | H-1->L+2 (6%) |
| **5** | 390.8311 | 0.013 | H-1->L+1 (64%), HOMO->L+2 (15%) | H-1->LUMO (3%), H-1->L+2 (8%), H-1->L+3 (3%) |
| **6** | 387.2543 | 0.0052 | HOMO->L+3 (90%) | |
| **7** | 369.9559 | 0.0175 | H-2->LUMO (88%) | H-1->L+3 (3%) |
| **8** | 369.5479 | 0.122 | H-1->L+1 (12%), H-1->L+2 (49%), HOMO->L+4 (10%) | H-2->LUMO (4%), H-1->L+3 (8%), H-1->L+4 (4%), HOMO->L+1 (5%) |
| **9** | 362.2702 | 0.1145 | H-1->L+2 (14%), HOMO->L+4 (62%) | H-1->L+3 (8%), HOMO->L+5 (6%) |
| **10** | 362.1114 | 0.0613 | H-1->L+3 (65%), HOMO->L+4 (20%) | H-1->L+2 (3%) |
| **11** | 356.4173 | 0.0443 | H-1->L+4 (88%) | H-1->L+2 (4%) |
| **12** | 348.6596 | 0.0957 | HOMO->L+5 (75%) | H-2->L+2 (3%), H-1->L+2 (2%), H-1->L+3 (4%), HOMO->L+4 (3%), HOMO->L+7 (5%) |
| **13** | 346.9618 | 0.011 | H-3->LUMO (87%) | H-1->L+3 (3%) |
| **14** | 337.7372 | 0.0094 | H-1->L+5 (72%) | H-2->L+1 (5%), HOMO->L+6 (8%), HOMO->L+7 (8%) |
| **15** | 334.9 | 0.0114 | H-2->L+1 (19%), H-1->L+5 (13%), HOMO->L+7 (43%) | H-5->LUMO (3%), H-2->L+2 (3%), HOMO->L+5 (8%) |

1. **^1^H and ^13^C NMR Spectra**

**
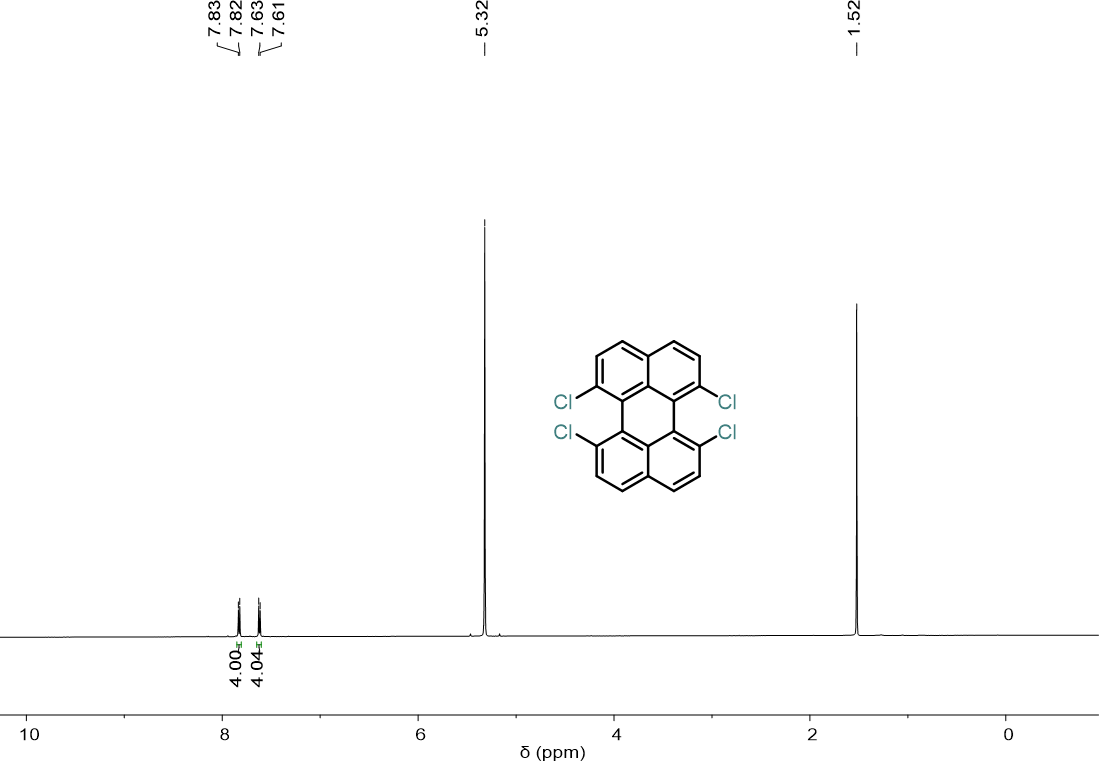
**

**Figure S28.** ^1^H NMR spectrum (600 MHz) of compound **Per-4Cl** in CD_2_Cl_2_ at 298 K.

**
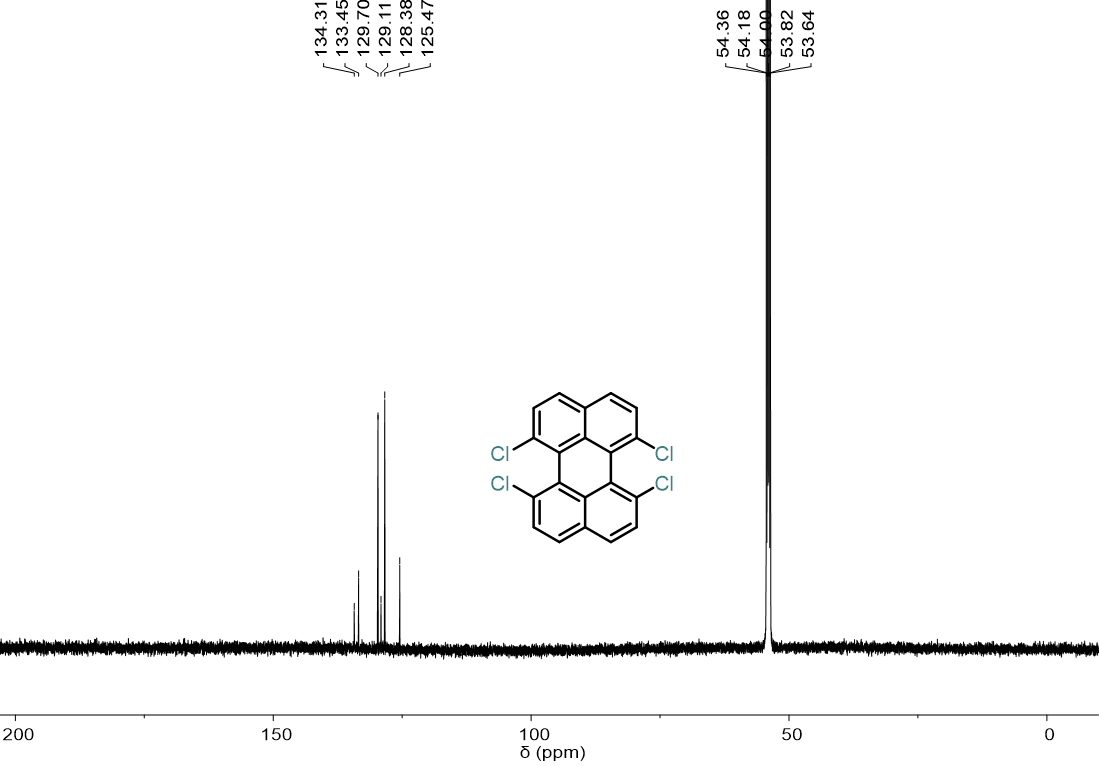
**

**Figure S29.** ^13^C NMR spectrum (150 MHz) of compound **Per-4Cl** in CD_2_Cl_2_ at 298 K.


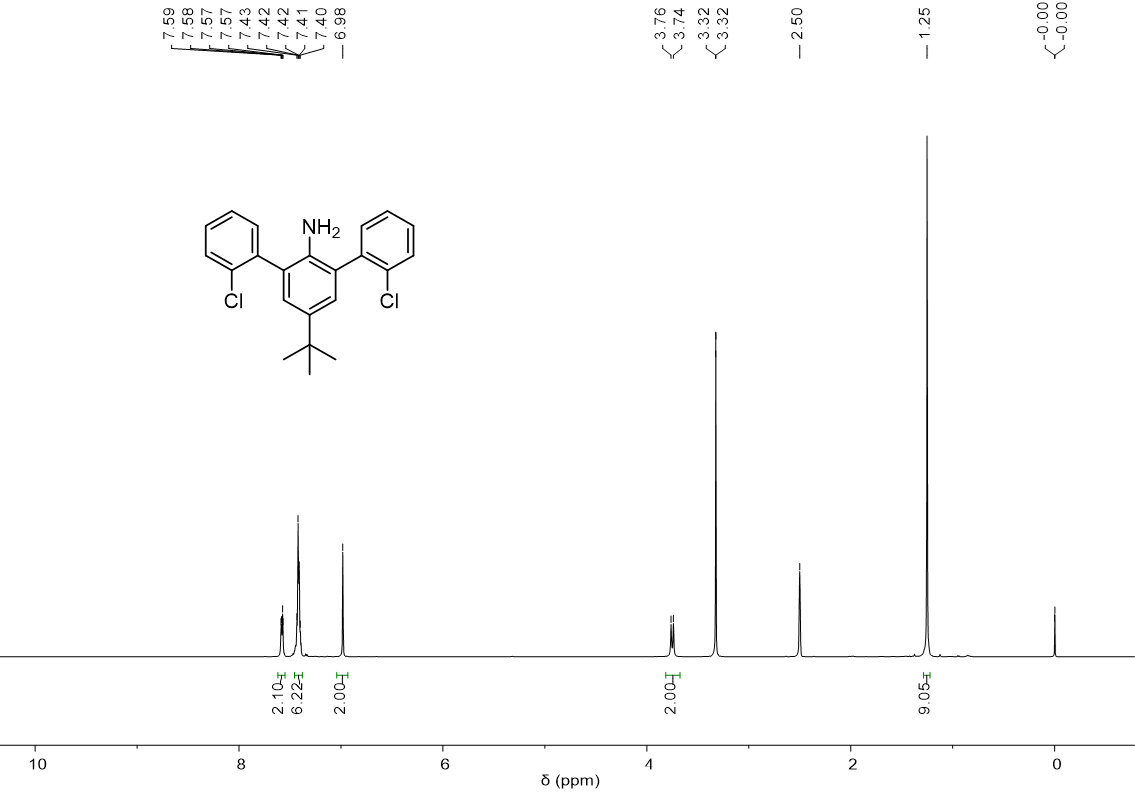


**Figure S30.** ^1^H NMR spectrum (500 MHz) of 5'-(*tert*-butyl)-2,2''-dichloro-[1,1':3',1''-terphenyl]-2'-amine in DMSO-*d6* at 298 K.


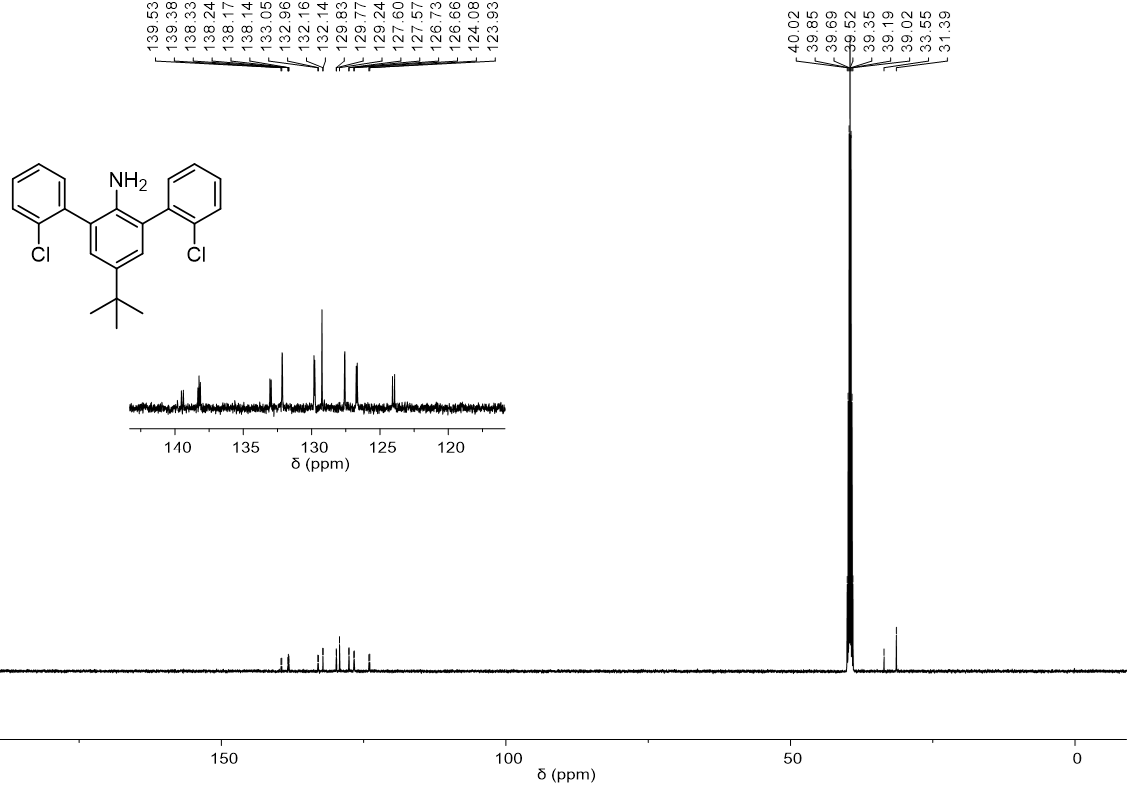


**Figure S31.** ^13^C NMR spectrum (125 MHz) of 5'-(*tert*-butyl)-2,2''-dichloro-[1,1':3',1''-terphenyl]-2'-amine in DMSO-*d6* at 298 K.


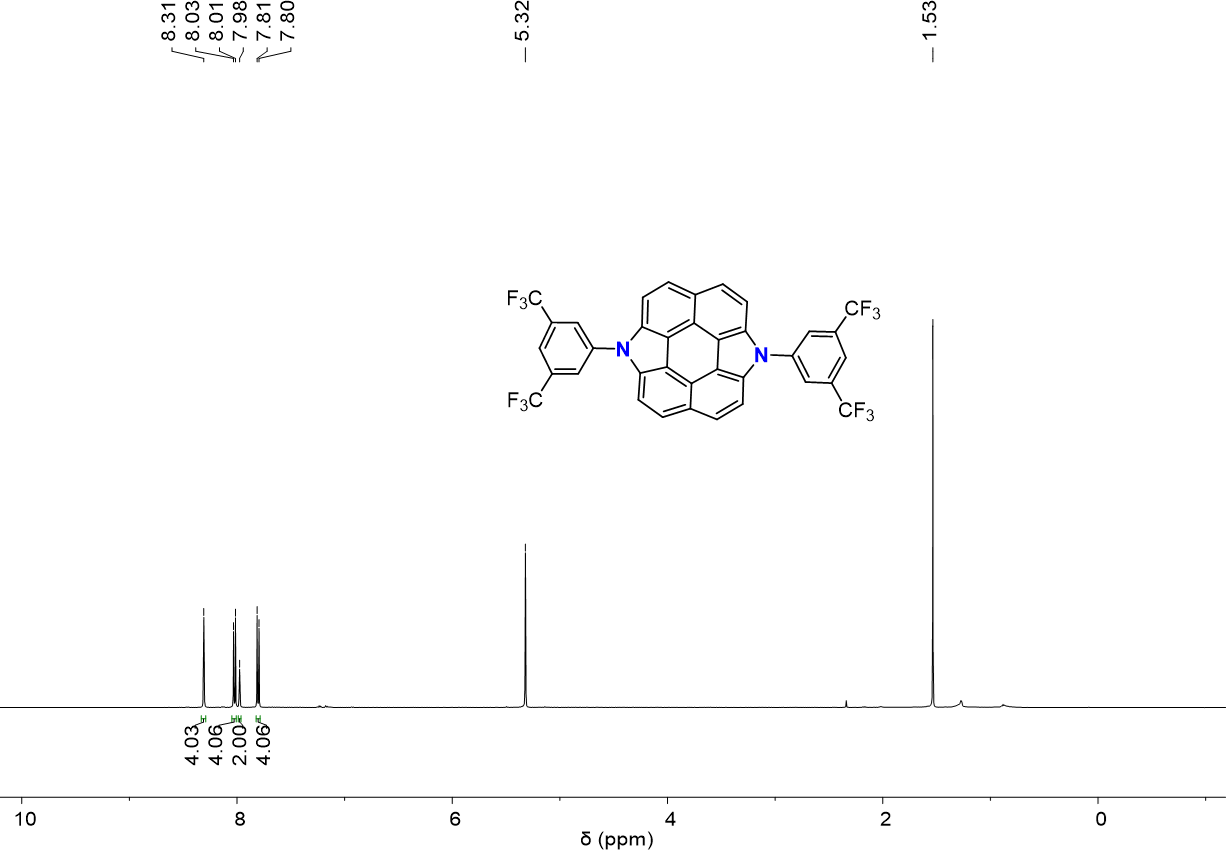


**Figure S32.** ^1^H NMR spectrum (500 MHz) of compound **1a** in CD_2_Cl_2_ at 298 K.


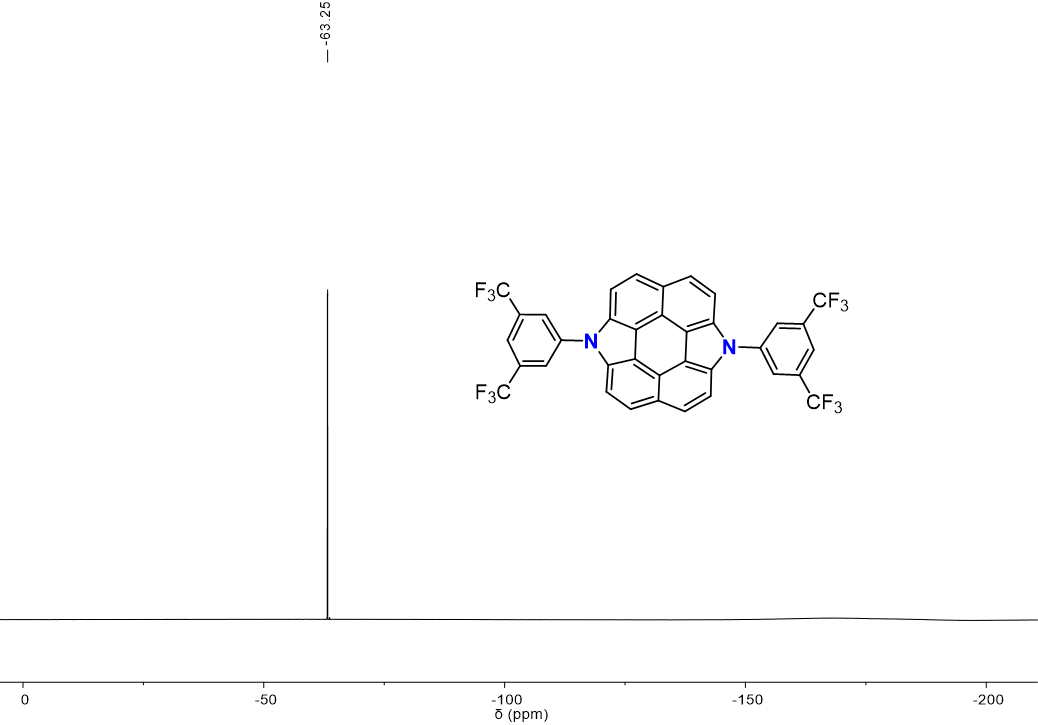


**Figure S33.** ^19^F NMR spectrum (471 MHz) of **1a** in CD_2_Cl_2_ at 298 K.


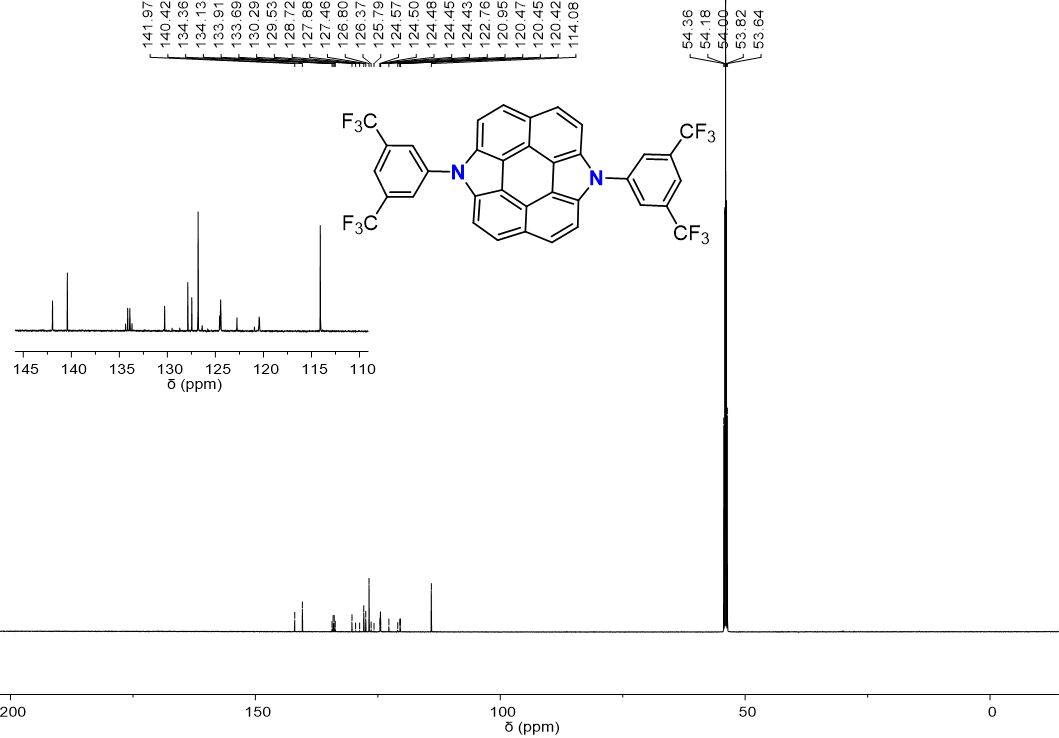


**Figure S34.** ^13^C NMR spectrum (125 MHz) of compound **1a** in CD_2_Cl_2_ at 298 K.


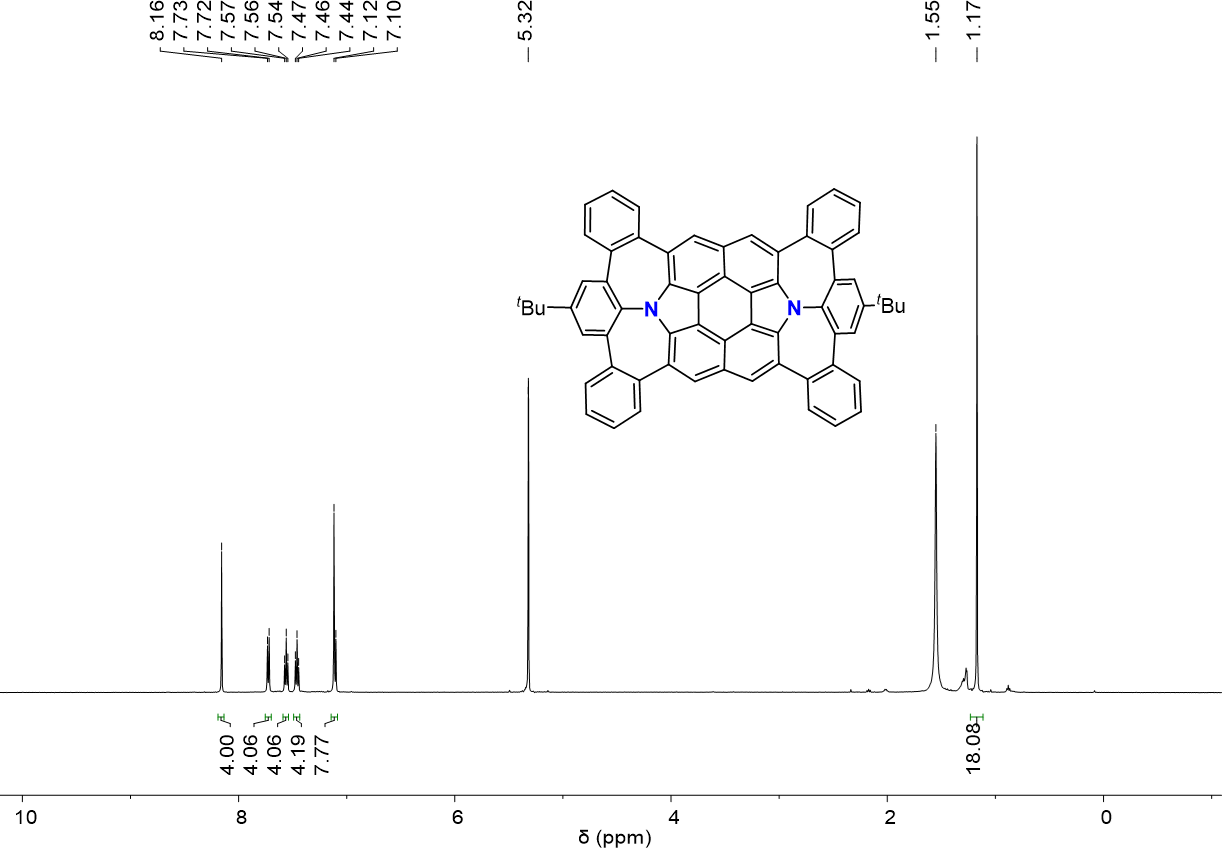


**Figure S35.** ^1^H NMR spectrum (500 MHz) of compound **2** in CD_2_Cl_2_ at 298 K.


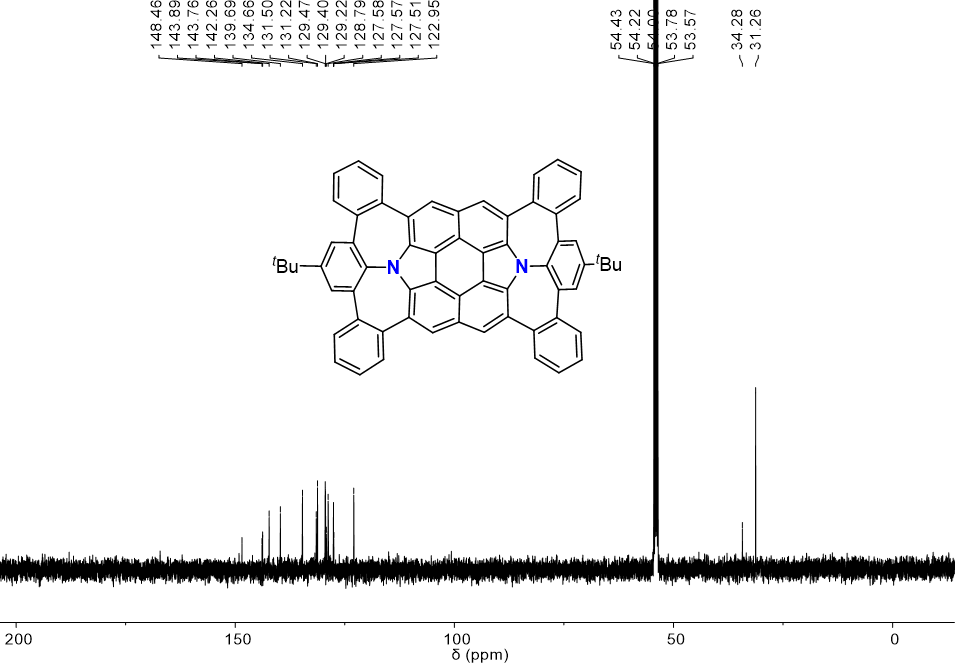


**Figure S36.** ^13^C NMR spectrum (125 MHz) of compound **2** in CD_2_Cl_2_ at 298 K.

**Figure S37.** Partial ^1^H-^1^H 2D COSY NMR spectrum (600 MHz) of **2** measured in CD_2_Cl_2_ at 298 K.

**Figure S38.** ^1^H-^1^H 2D COSY NMR spectrum (600 MHz) of **2** measured in CD_2_Cl_2_ at 298 K.


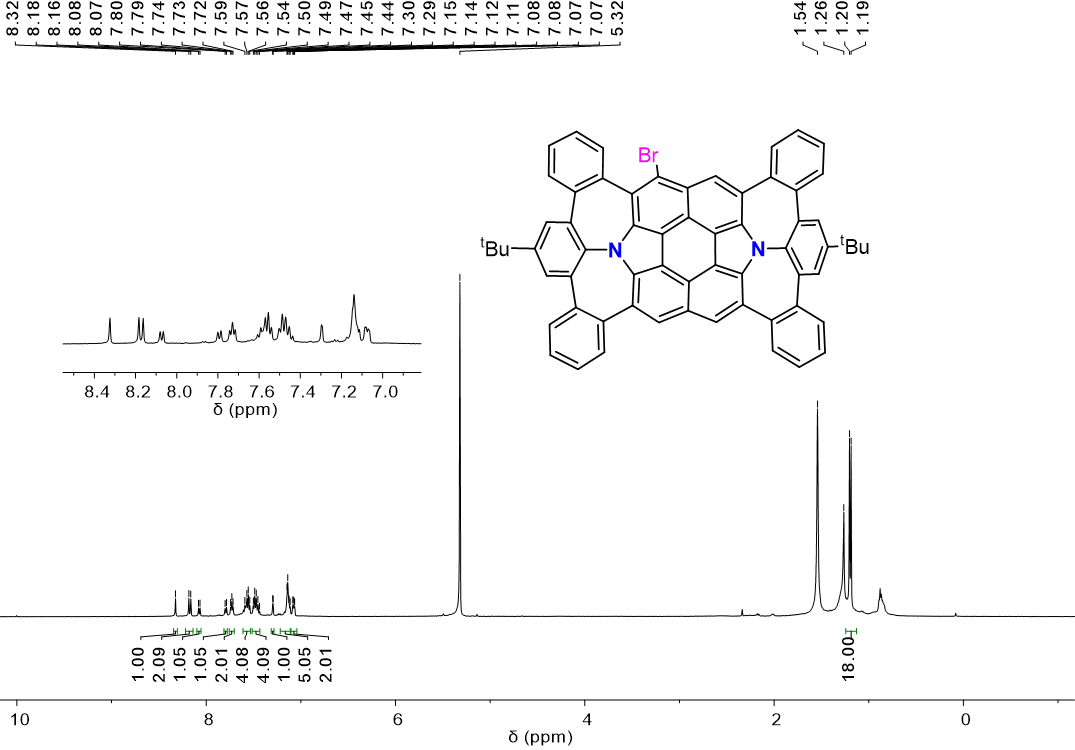


**Figure S39.** ^1^H NMR spectrum (500 MHz) of **3** measured in CD_2_Cl_2_ at 298 K.


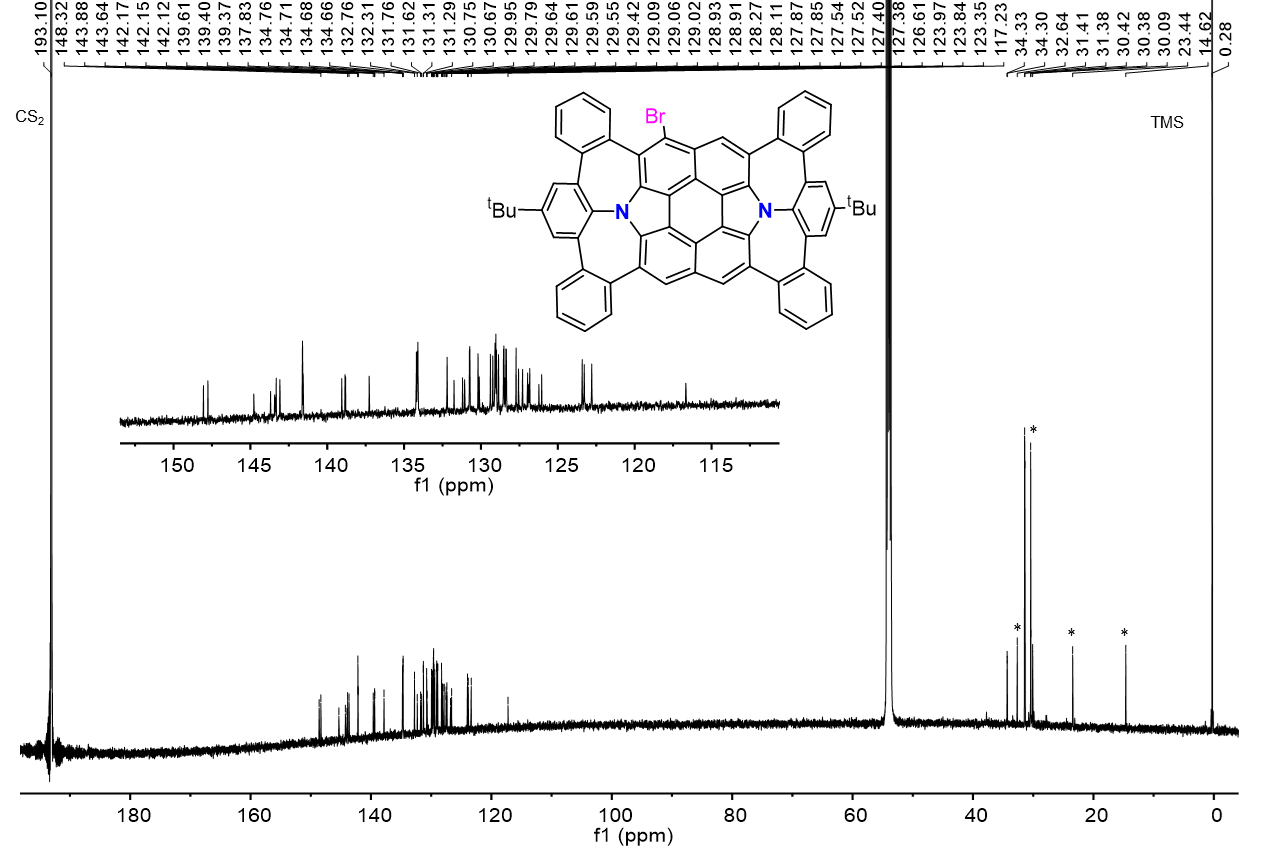


**Figure S40.** ^1^H NMR spectrum (600 MHz) of **3** measured in CD_2_Cl_2_/CS_2_ at 298 K. The asterisks represent the signals form grease.


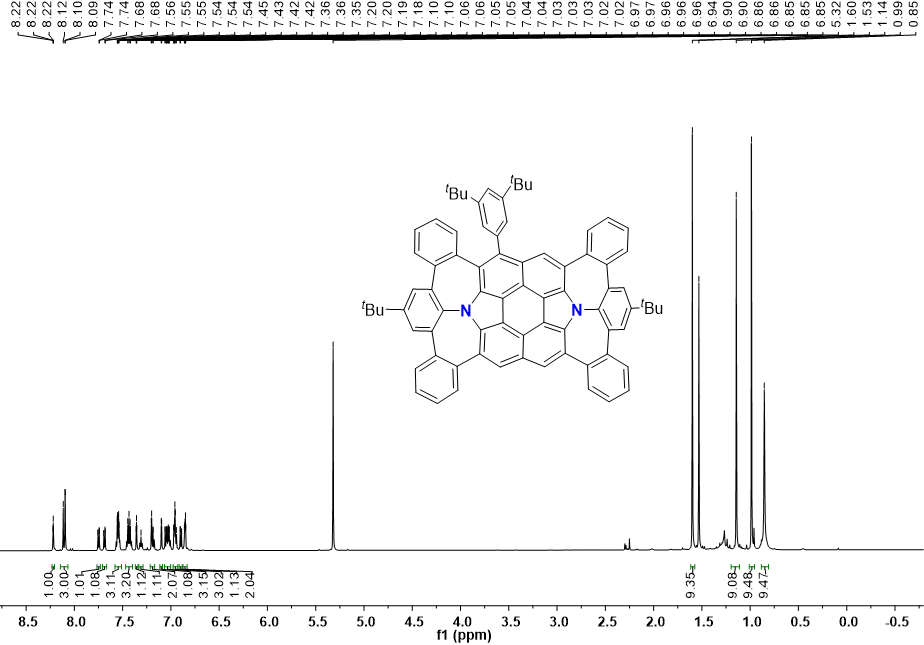


**Figure S41.** ^1^H NMR spectrum (600 MHz) of **4a** in CD_2_Cl_2_ at 298 K.


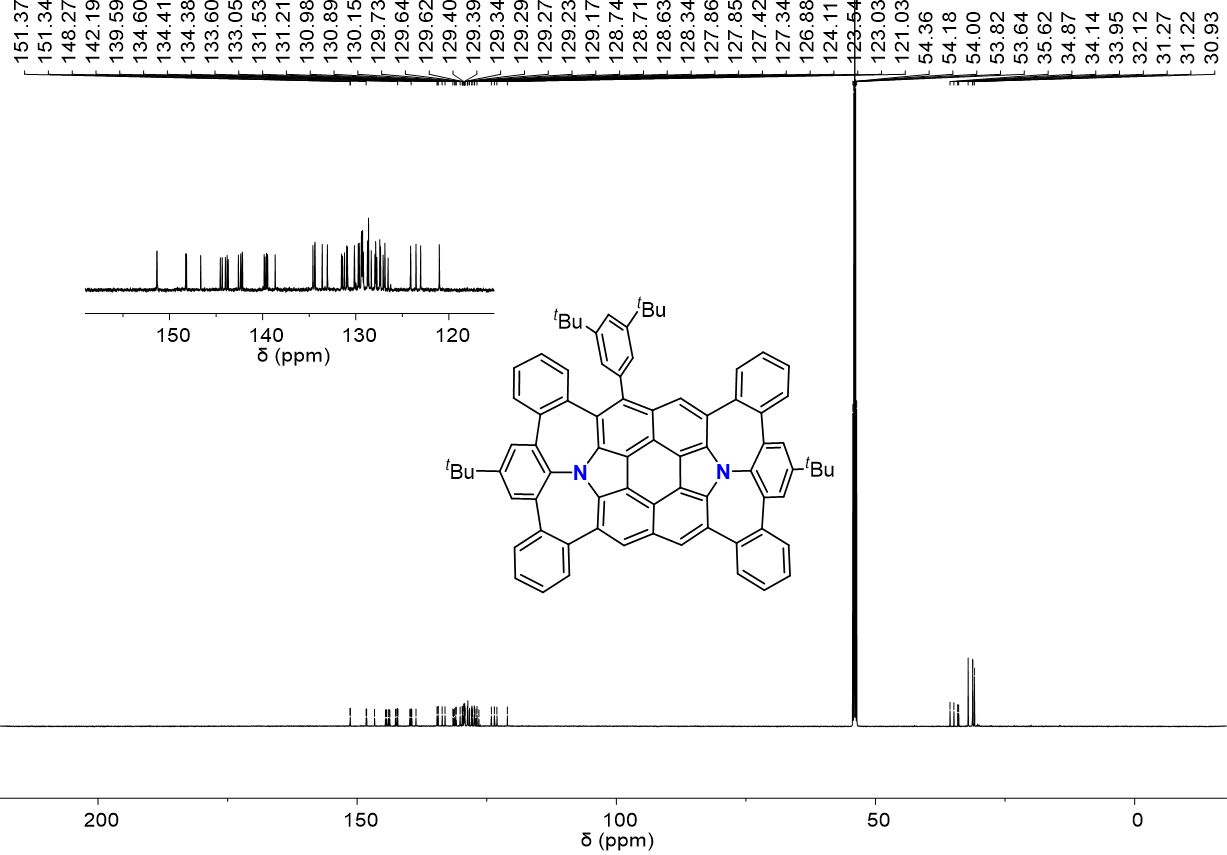


**Figure S42.** ^13^C NMR spectrum (150 MHz) of **4a** in CD_2_Cl_2_ at 298 K.


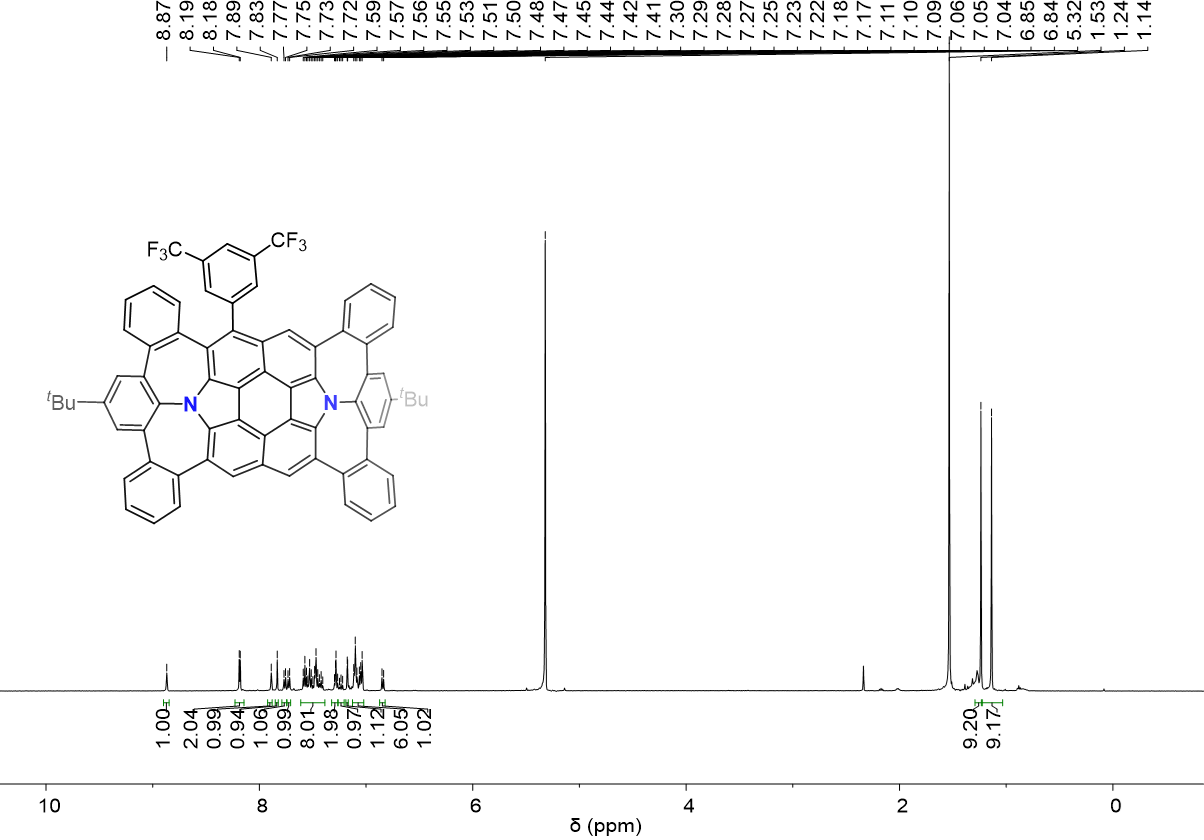


**Figure S43.** ^1^H NMR spectrum (500 MHz) of **4b** in CD_2_Cl_2_ at 298 K.


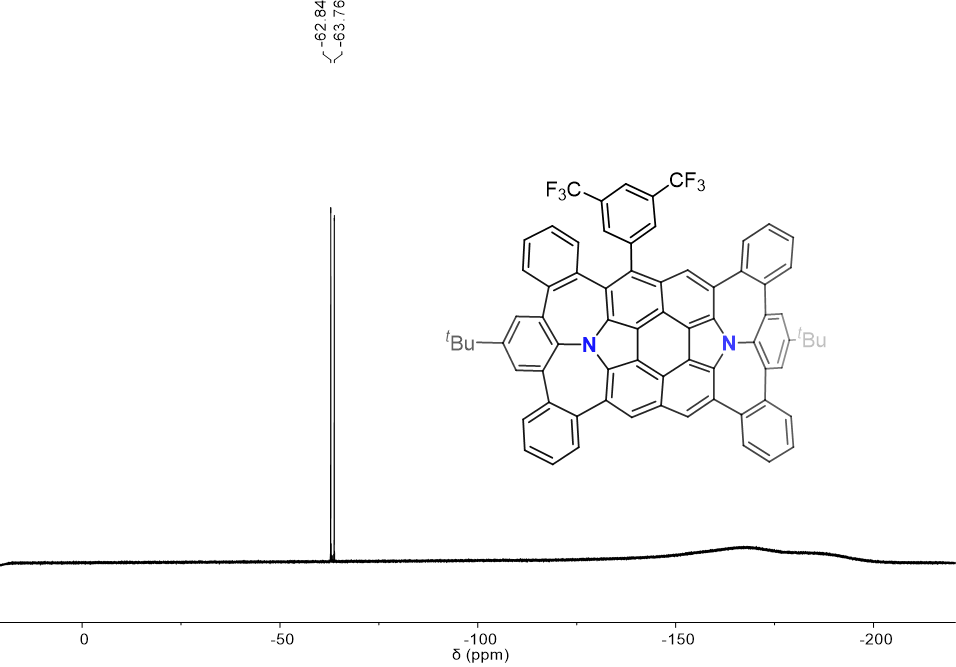


**Figure S44.** ^19^F NMR spectrum (471 MHz) of **4b** in CD_2_Cl_2_ at 298 K.


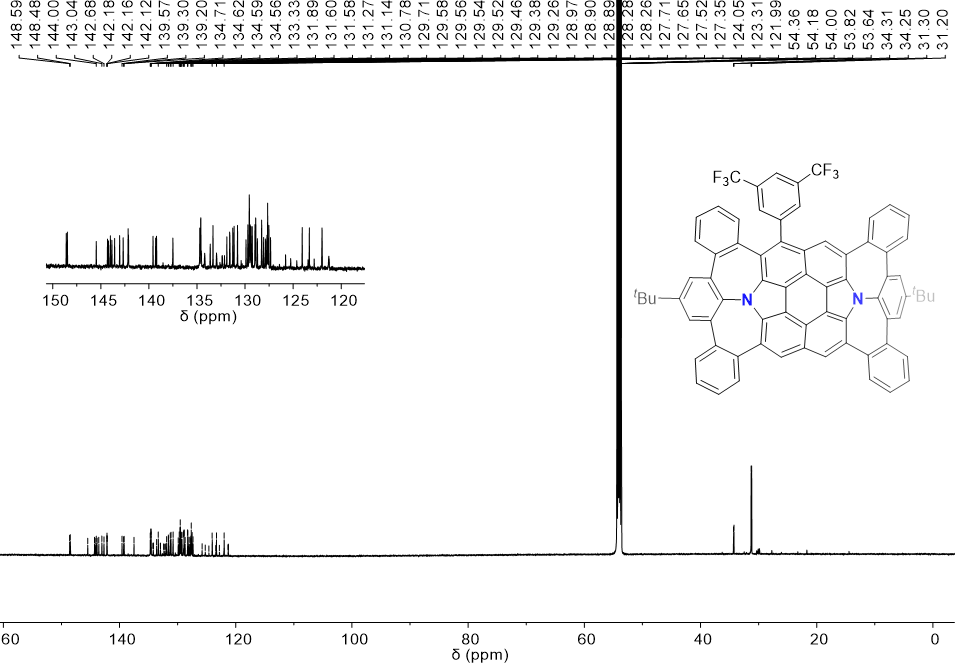


**Figure S45.** ^13^C NMR spectrum (150 MHz) of **4b** in CD_2_Cl_2_ at 298 K.

1. **Mass Spectra**


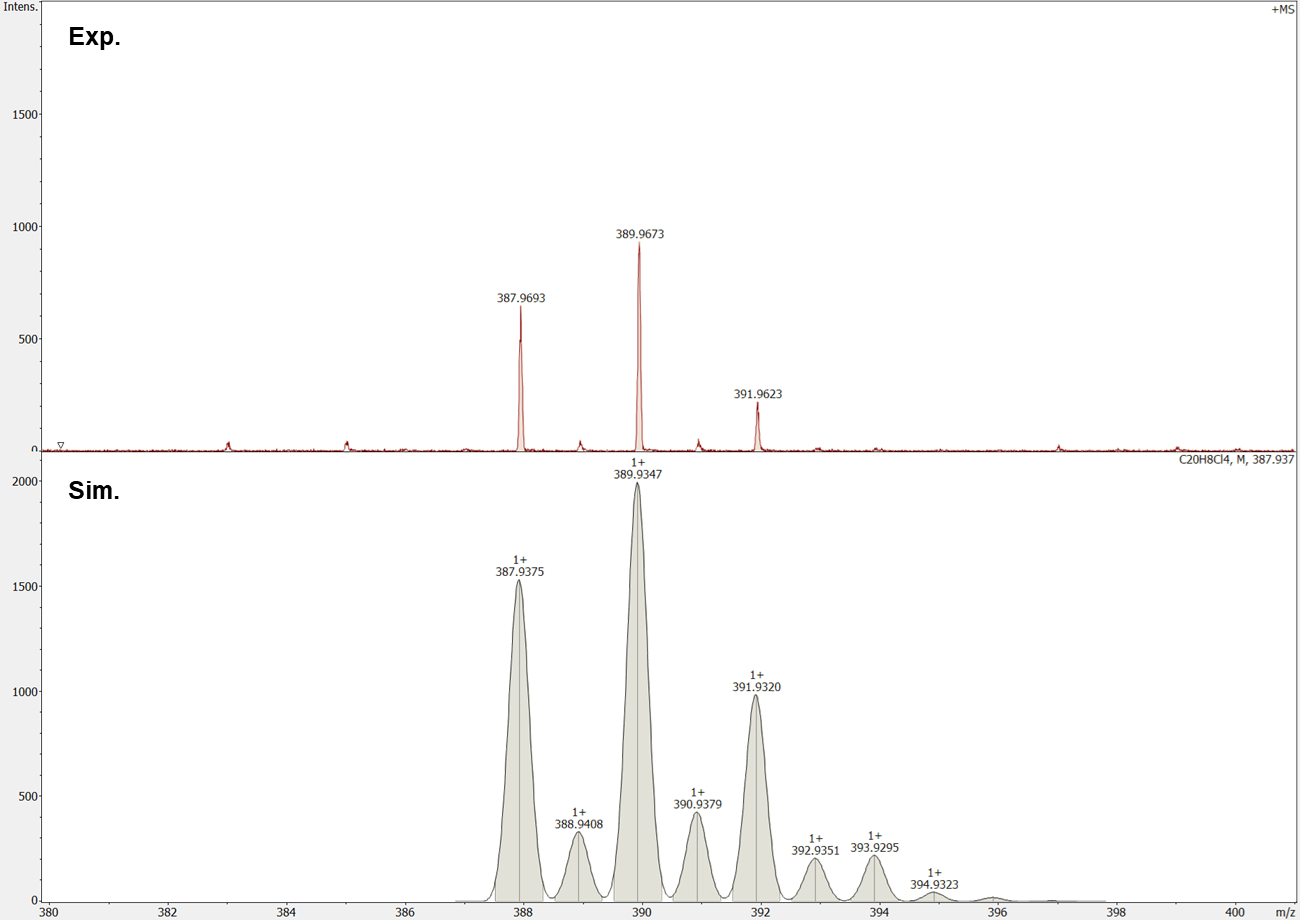


**Figure S46.** Mass spectrum (MALDI-TOF) of **Per-4Cl**.


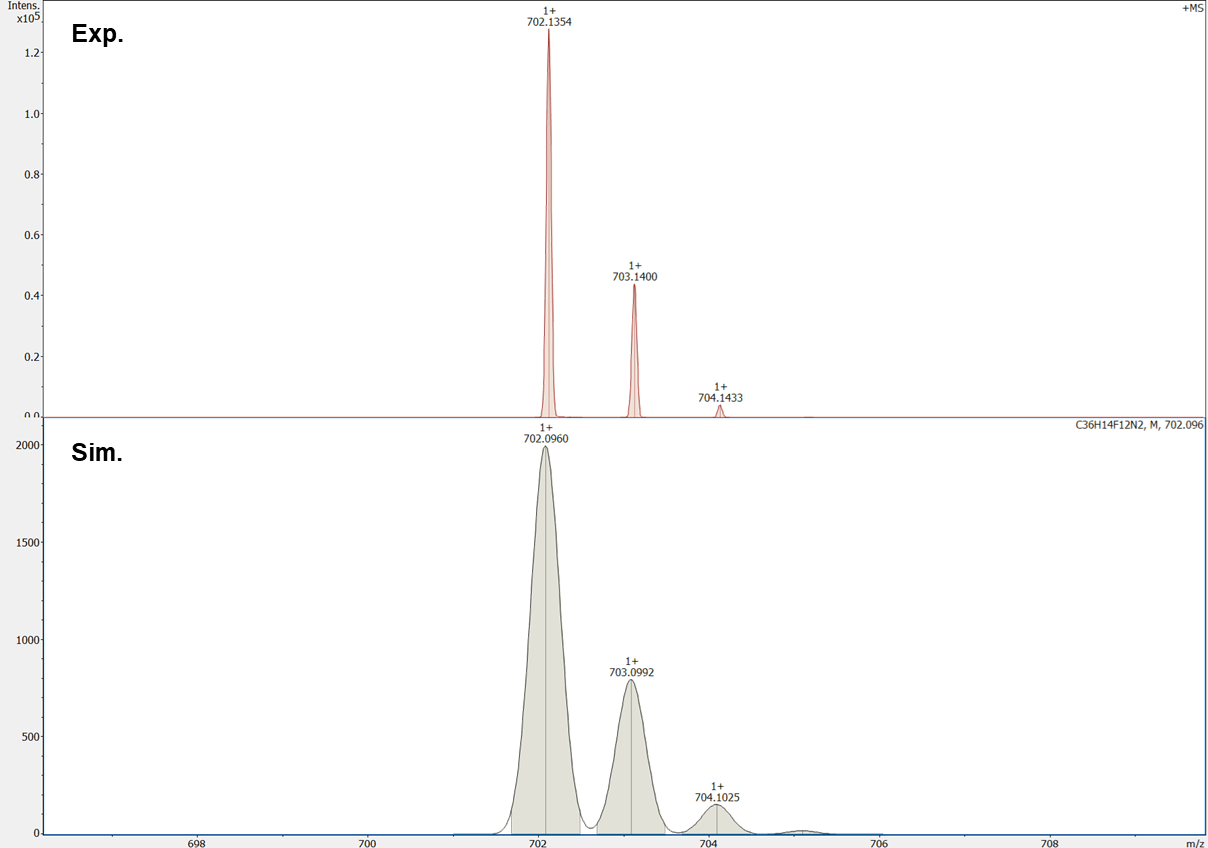


**Figure S47.** Mass spectrum (MALDI-TOF) of **1a**.


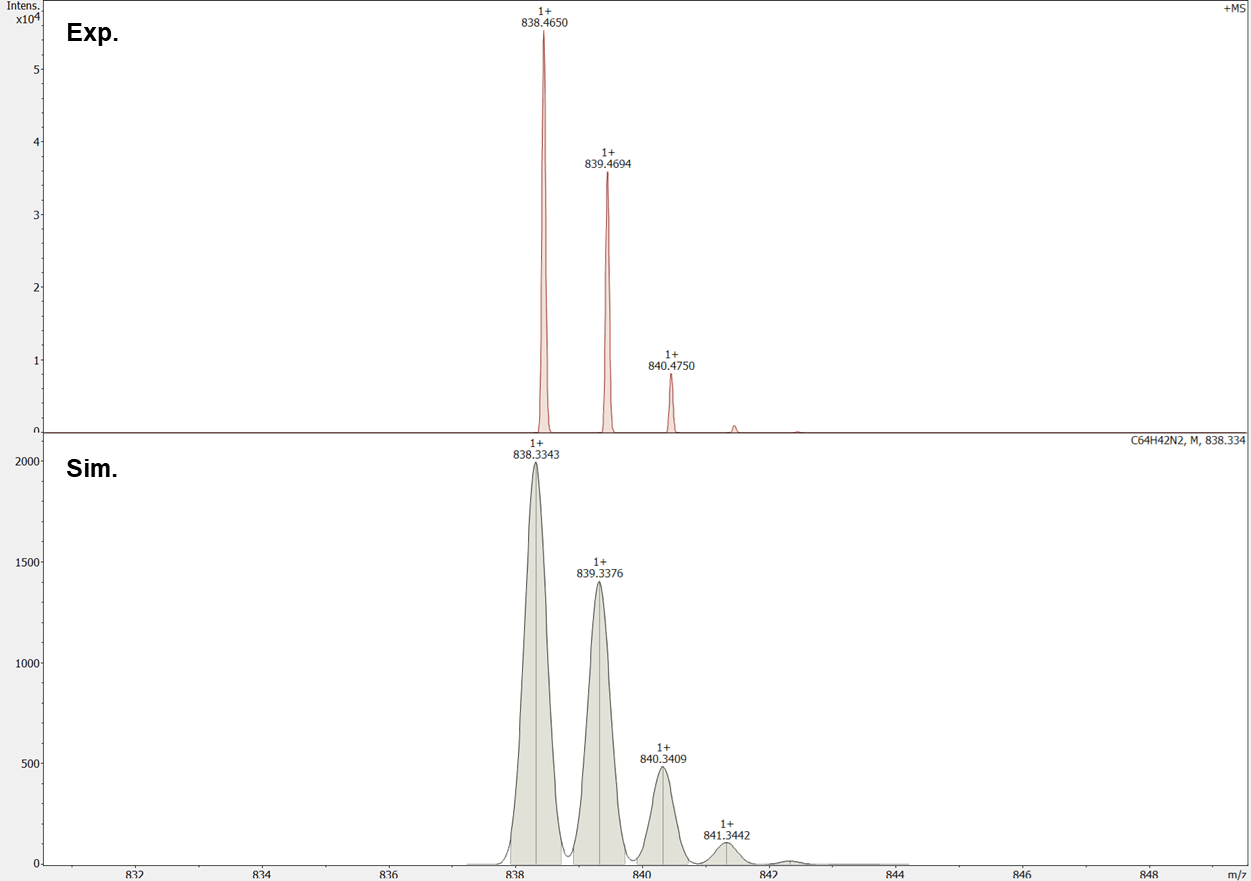


**Figure S48.** Mass spectrum (MALDI-TOF) of **2**.


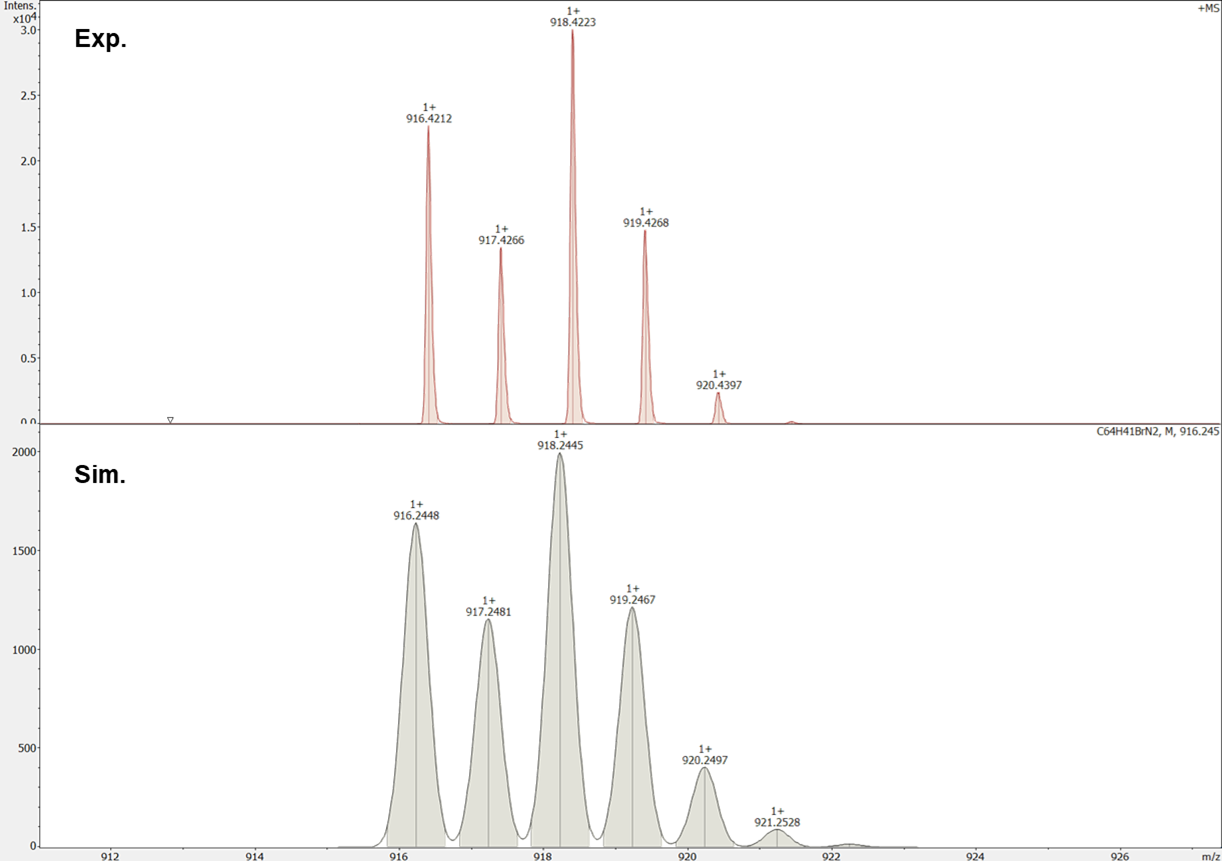


**Figure S49.** Mass spectrum (MALDI-TOF) of **3**.


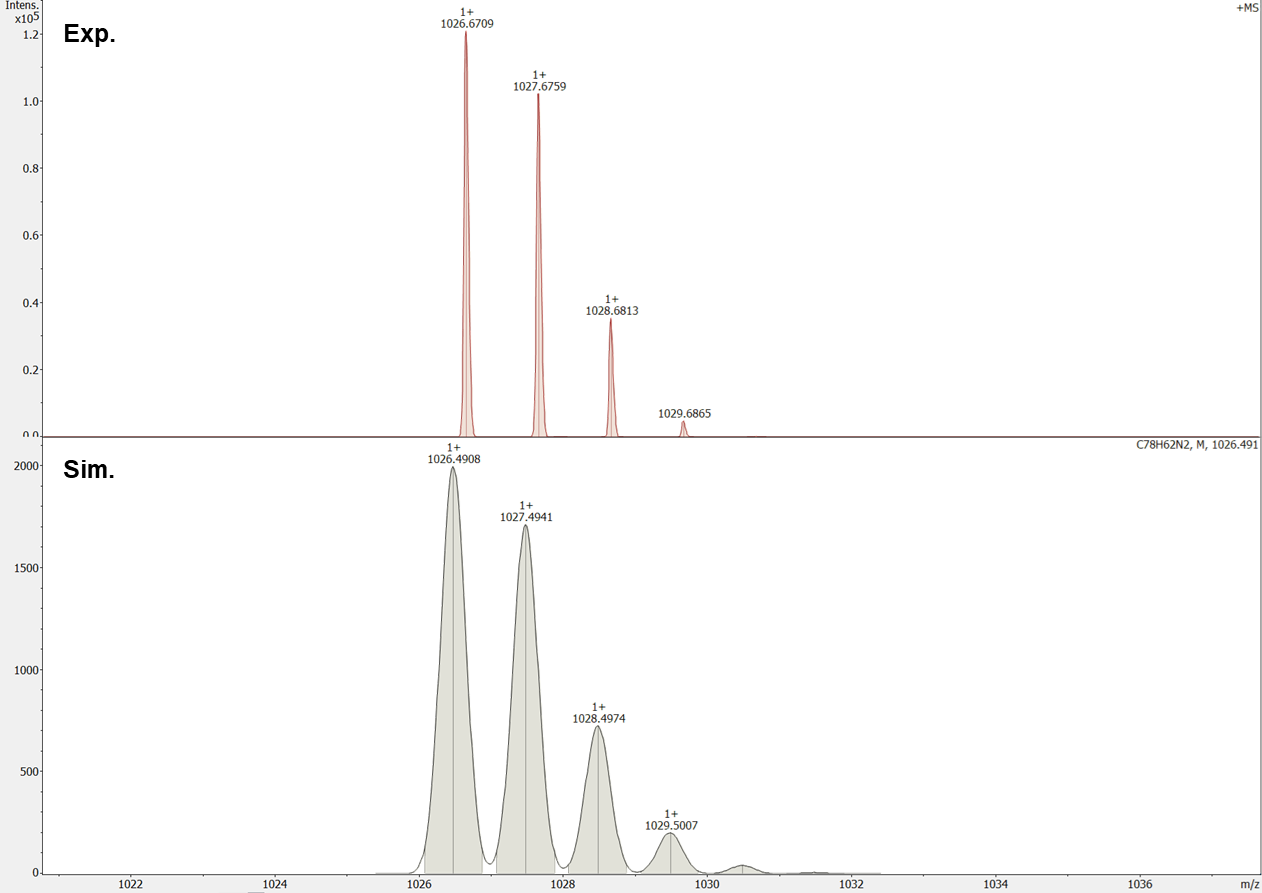


**Figure S50.** Mass spectrum (MALDI-TOF) of **4a**.


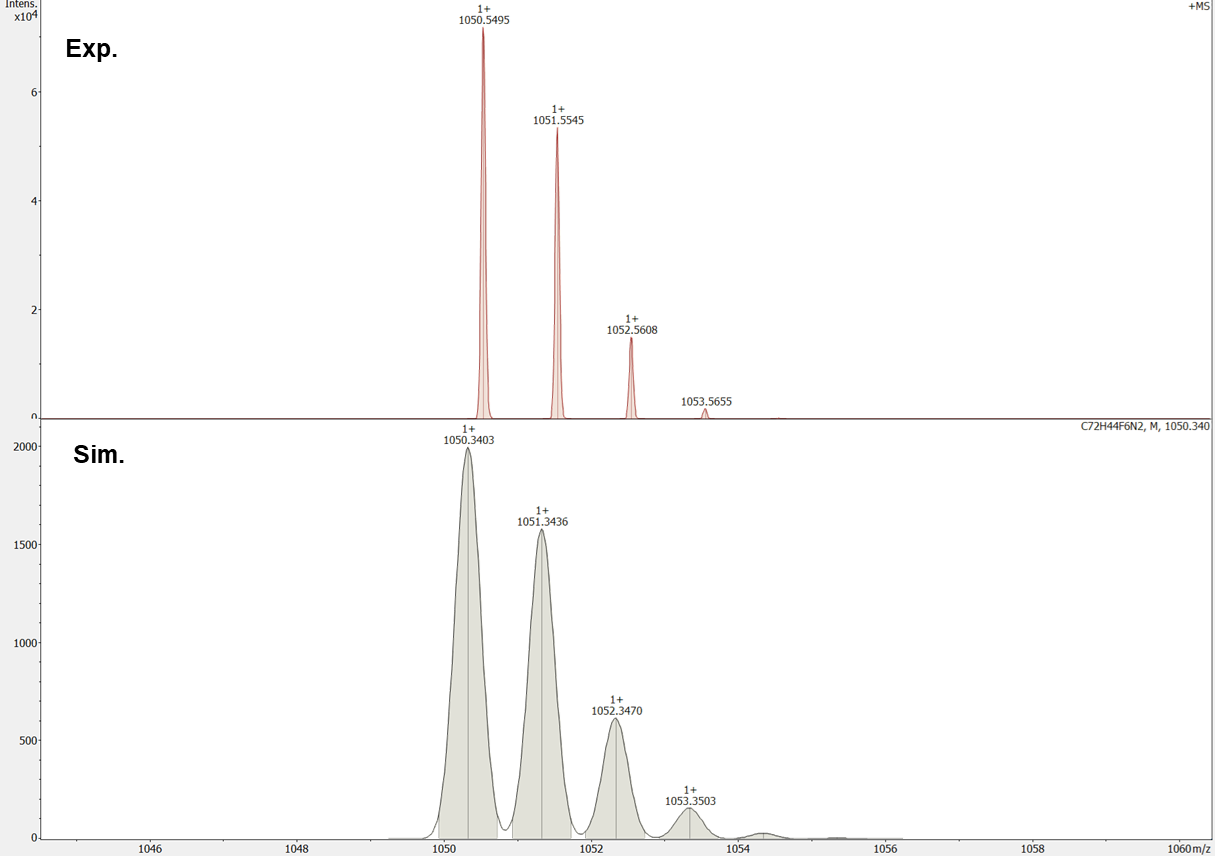


**Figure S51.** Mass spectrum (MALDI-TOF) of **4b**.

1. **References**

[S1] Thordarson, P. Determining association constants from titration experiments in supramolecular chemistry. *Chem. Soc. Rev.* **2011**, *40*, 1305-1323

[S2] Brynn Hibbert, D.; Thordarson, P. The death of the Job plot, transparency, open science and online tools, uncertainty estimation methods and other developments in supramolecular chemistry data analysis. *Chem. Commun.* **2016**, *52*, 12792-12805

[S3] Zagranyarski, Y.; Chen, L.; Jänsch, D.; Gessner, T.; Li, C.; Müllen, K. Toward Perylene Dyes by the Hundsdiecker Reaction. *Org. Lett.* **2014**, *16*, 2814-2817

[S4] Kost, D., Carlson, E. H., Raban, M. The validity of approximate equations for kc in dynamic nuclear magnetic resonance. *J. Chem. Soc. D*, **1971**, *13*, 656-657.

[S5] Wang, Y., Stretton, A. D., McConnell, M. C., Wood, P. A., Parsons, S., Henry, J. B., Mount, A. R., Galow, T. H. 1, 4, 5, 8, 9, 12-hexamethyltriphenylene. A molecule with a flipping twist. *J. Am. Chem. Soc.*, **2007**, *129*, 13193-13200.

[S6] Ravat, P. Carbo[n]helicenes Restricted to Enantiomerize: An Insight into the Design Process of Configurationally Stable Functional Chiral PAHs. *Chem. Eur. J.* **2021**, *27*, 3957-3967

[S7] Gaussian 16, Revision C.01, M. J. Frisch, G. W. Trucks, H. B. Schlegel, G. E. Scuseria, M. A. Robb, J. R. Cheeseman, G. Scalmani, V. Barone, G. A. Petersson, H. Nakatsuji, X. Li, M. Caricato, A. V. Marenich, J. Bloino, B. G. Janesko, R. Gomperts, B. Mennucci, H. P. Hratchian, J. V. Ortiz, A. F. Izmaylov, J. L. Sonnenberg, D. Williams-Young, F. Ding, F. Lipparini, F. Egidi, J. Goings, B. Peng, A. Petrone, T. Henderson, D. Ranasinghe, V. G. Zakrzewski, J. Gao, N. Rega, G. Zheng, W. Liang, M. Hada, M. Ehara, K. Toyota, R. Fukuda, J. Hasegawa, M. Ishida, T. Nakajima, Y. Honda, O. Kitao, H. Nakai, T. Vreven, K. Throssell, J. A. Montgomery, Jr., J. E. Peralta, F. Ogliaro, M. J. Bearpark, J. J. Heyd, E. N. Brothers, K. N. Kudin, V. N. Staroverov, T. A. Keith, R. Kobayashi, J. Normand, K. Raghavachari, A. P. Rendell, J. C. Burant, S. S. Iyengar, J. Tomasi, M. Cossi, J. M. Millam, M. Klene, C. Adamo, R. Cammi, J. W. Ochterski, R. L. Martin, K. Morokuma, O. Farkas, J. B. Foresman, and D. J. Fox, Gaussian, Inc., Wallingford CT, 2016.

[S8] Lu, T.; Chen, F. Multiwfn: A multifunctional wavefunction analyzer, *J. Comput. Chem.* **2012**, *33*, 580-592.

[S9] Tian Lu, Qinxue Chen, Independent gradient model based on Hirshfeld partition: A new method for visual study of interactions in chemical systems, *J. Comput. Chem.*,**2022**, *43*, 539-555

[S10] Humphrey, W.; Dalke, A.; Schulten, K., VMD - Visual Molecular Dynamics, *J. Molec. Graphics*, **1996**, *14*, 33-38

[S11] Geuenich, D.; Hess, K.; Köhler, F.; Herges, R. Anisotropy of the Induced Current Density (ACID), a General Method to Quantify and Visualize Electronic Delocalization. *Chem. Rev.* **2005**, *105*, 3758-3772.
